# Analysis of the Changes in Physicochemical Properties and Microbial Communities During Fermentation of Sweet Fermented Rice

**DOI:** 10.3390/foods14071121

**Published:** 2025-03-24

**Authors:** Jiaqiong Wan, Ping Tian, Xiaozhen Liu, Hanyao Zhang

**Affiliations:** Key Laboratory for Forest Resources Conservation and Utilization in the Southwest Mountains of China, Ministry of Education, Southwest Forestry University, Kunming 650224, China; wjqlzp522624@swfu.edu.cn (J.W.); yiwangbaoyq@swfu.edu.cn (P.T.); 15198729095@swfu.edu.cn (X.L.)

**Keywords:** sweet wine, high-throughput sequencing, physicochemical properties, microbial communities and differences, dominant species

## Abstract

As a traditional rice wine, sweet fermented rice (SFR) is widely loved because of its unique flavor and high nutritional value. However, the physicochemical properties, microbial community composition, and metabolic pathway changes during the fermentation process of sweet wine have not been evaluated, and these changes can lead to unstable SFR quality. In this study, we used high-throughput sequencing technology to analyze and elucidate the dynamic changes in the microbial community, metabolic pathways, and carbohydrate enzyme functions in traditional SFR fermentation broth. The results revealed that *Rhizopus* abundance = 160,943.659 and *Wickerhamomyces* abundance = 241,660.954 were the predominant fungal genera in the fermentation process from the beginning (A0) to the end (A43) of SFR fermentation. The results of the diversity analysis revealed that the structure and composition of the microbial communities first increased but then decreased. Metabolic pathway analysis showed that energy production and conversion, carbohydrate transport, and amino acid transport were the most active metabolic pathways in fermentation. Moreover, the three primary functions of glycosyltransferases (GTs), glycoside hydrolases (GHs), and carbohydrate-binding modules (CBMs) in carbohydrate enzyme analysis were involved in the whole fermentation process. This study only provides some insight into the dynamic changes in the microbial population of SFR single samples prepared under fixed conditions. It provides a reference for optimizing the physicochemical properties of SFR fermentation broth, controlling the microbial community structure, optimizing fermentation conditions, and improving product quality.

## 1. Introduction

Sweet fermented rice (SFR) is a traditional food made from glutinous rice as a raw material, which is cooked and then mixed with sweet wine koji for fermentation [1,2]. Fermented glutinous rice refers mainly to the solid part of liquefied glutinous rice, whereas the liquid part is sweet rice wine. Sweet wine is rich in carbohydrates, proteins, vitamins, minerals, and other nutrients; has beneficial effects on qi (enhancing human function and resistance); promotes body fluid and blood circulation; and is a popular nutritional and health food [3,4]. SFR has a low alcohol content of 0.5% to 14% (*v*/*v*), unlike other wines brewed with rice or millet, which have a higher alcohol content of 14% to 20% (*v*/*v*) [5,6]. Studies have confirmed that glutinous rice is a food composed of various macromolecules with edible value and some healthcare value [7]. Sweet wine is an essential part of traditional Chinese food culture. Rice wine also has biological activities, such as its antioxidant [8] and anti-inflammatory properties [9] and ability to reduce doxorubicin-induced cardiotoxicity [10].

In addition, SFR fermentation also requires the addition of a specific starter, also known as koji. Distiller koji may have originated in China more than 5000 years ago, and a record of its use was found in “Qi Min Yao Shu” in the 6th century (544 AD) [11]. Sake brews are rich in microflora, and during simultaneous saccharification/liquefaction and alcoholic fermentation, the brews provide microflora that converts rice starch into sake brew. This process, in which microorganisms are grown and cultured on a large scale to undergo chemical and physiological changes to produce and accumulate large quantities of metabolites, is called fermentation. It has been reported that various bacteria produce hydrolytic enzymes, glucoamylases, proteases, esterases, etc., to degrade raw substrates during fermentation, all of which may lead to the accumulation of aroma-related compounds or secondary metabolites and intermediates [12,13]. However, other researchers have shown that fungi are more crucial than bacteria in this process, as they determine the productivity, quality, and sensory characteristics of liquefied fermented rice [14]. Among fungi, filamentous fungi, molds, and yeasts play crucial roles by secreting many hydrolytic enzymes and producing beneficial metabolites [15]. The primary enzymes (including amylase, glucoamylase, protease, lipase, and xylanase) hydrolyze macromolecules such as starch, proteins, and lipids into dextrin, maltose, glucose, small peptides, and fatty acids. *Rhizoctonia*, yeasts, and other fungi subsequently use these substances for growth [16]. For example, Rhizopus can fully convert starch in rice into fermentable sugars [17]. However, yeasts use fermentable sugars to produce alcohol and carbon dioxide, determining the fermentation rate of SFR and promoting its taste and quality. The glycolytic metabolism of yeast can lead to ethanol production, while other yeasts can affect sensory characteristics such as taste, aroma, and mouthfeel [18]. The output of the SFR is also influenced by the environment in different regions. The raw materials and additives used in sweet rice wine also lead to differences in SFR starters, which may endow SFR with different sensory characteristics, flavors, and other characteristics [19].

SFR fermentation production is a complex process that takes place in an open environment, where a variety of microorganisms are constantly mixed in from the fermenter, the raw materials, and the environment. The complex microbial activities during the SFR fermentation produce enzymes, alcohols, and other molecules while promoting the release of phenolic compounds, which create the unique taste, flavor, and nutritional properties of the sake [20]. For example, in wine production, it is crucial to use fermentation agents to increase the content of free-form phenolic compounds (aglycones) [21]. SFR fermentation follows traditional methods, i.e., an uncontrolled fermentation process, which results in inconsistent tastes. According to existing reports, most studies compare only the differences in nutritional composition, antioxidant capacity, quality, taste, and flavor of sweet wine from different sources through sensory evaluation [22,23]. Lu et al. prepared cinnamon sweet wine from mandarins and glutinous rice by fermentation with the mere addition of *Rhizobium* but also ignored the effect of other microorganisms entering the fermentation process from the external environment [24]. However, few studies have evaluated the evolution of physicochemical properties and microbiota during traditional SFR fermentation, and the interactions between the microbiota and physicochemical properties have not yet been elucidated to explain this, for which it is first necessary to understand the dynamic changes in the composition of microbial communities present in SFR. Indeed, extensive research has been conducted to elucidate the composition and dynamics of the microbial communities associated with Chinese rice wine [25,26]. Most of these studies have been based on traditional molecular methods, such as culture-dependent methods and culture-independent polymerase chain reaction (PCR)–denaturing gradient gel electrophoresis techniques [27,28,29]. However, these methods have certain limitations in comprehensively reflecting the actual microbial diversity in a sample because of their low flux and low sensitivity. It is difficult for all of the above techniques to differentiate species with a population density of less than 10^3^ CFU/g or two orders of magnitude lower than the most abundant member of these communities [30,31]. High-throughput sequencing technology can perform detailed and comprehensive analyses of the transcriptome and genome data of a species. Therefore, this technology is called deep sequencing or next-generation sequencing (NGS). This technique has quantitative capabilities and can determine the abundance of species components in a sample. In addition, this technique is simple to perform, has a low cost, and has good result feasibility [32], which makes it faster and better than internal transcribed spacer identification (ITS PCR) and fluorescence ITS PCR capillary electrophoresis. This technique has been widely used to analyze the dynamics of microbial communities in various fermented foods and vegetables, such as Sichuan pickles [33], soy sauce [34], kiwi fruit [35], grape juice [36], and rice wine [37]. Metagenomic sequencing provides a theoretical basis for analyzing the relationships between microbial populations and specific flavors in these fermented foods.

In this study, we utilized high-throughput metagenomic rDNA sequencing (i.e., 16S rRNA and ITS gene sequencing). (1) Changes in pH, alcohol, total acid and total sugar contents, and microbial community composition of the SFR were analyzed. (2) Changes in and differences in microbial diversity during SFR fermentation were compared, and the relationships between the microbial community composition and pH, alcohol content, total acid content, and microbial community composition were compared. (3) Tracking studies of the metabolic pathways of SFR and the functional levels of carbohydrate enzymes were conducted. In this study, by analyzing the changes in the physicochemical properties of SFR and comparing the changes in microbial diversity during the fermentation of SFR, it is possible to hypothesize two strains that can be used for fermentation. Analyzing metabolic functions can predict the accumulation of aroma-related compounds or secondary metabolites and intermediates. In summary, this study only provides some assistance for the dynamic changes in the microbial population in SFR single samples prepared under fixed conditions. This study provides a basis for optimizing the physicochemical properties of SFR fermentation broth, controlling the microbial community structure, optimizing fermentation conditions, and improving product quality, which is highly important.

## 2. Materials and Methods

### 2.1. Materials and Reagents

Materials: glutinous rice (sold in Jianshui, China). Angel koji: a mixture containing root mold and yeast (Angel Yeast Co., Ltd., Yichang, China).

The reagent utilized was the True Lib DNA Library Rapid Prep Kit for Illumina (Yikesai Biotechnology Co., Ltd.,Suzhou, China.). Novozymes DNA clean beads (Nanjing Novozymes Biotechnology Co., Ltd., Nanjing, China.) and an Ex Kubitds DNA HS analysis kit (Suzhou, China, ExCell Bio, NGS00-3012) were used. The following DNA extraction kits were used: Power Oil ^®^ The DNA Isolation Kit, 2 × Tsingke Master Mix (green), Tsingke Agarose, and DL2000 Marker (Qingke Biotechnology Co., Ltd., Beijing, China). The PCR product gel recovery (magnetic bead method) kit, TBE, and EB were used. The BDT-original solution and buffer (for BDT dilution) were obtained (Qingke Biotechnology Co., Ltd., Beijing, China).

### 2.2. Instruments and Equipment

The following equipment was used: an Illumina platform NovaSeq 6000 (Illumina, Santa Clara, CA, USA), a Qubit analyzer (Santa Clara, CA, USA), and an Invitrogen instrument (2321603299).

The following instruments were used: an ELISA reader (Gene Company Limited, Synergy HTX, Hong Kong, China), an Eppendorf Legend Micro 21 high-speed centrifuge (Hamburg, Germany), a four-dimensional rotator (Qilinbeier Instrument Manufacturing Co., Ltd., Haimen, China), an oscillator (SI, G 560 E), a Vortex-Genie 2 vortex oscillator (Beijing Zhong xi Yuanda Technology Co., Ltd., Beijing, China), a condenser tube (Hongbin Food Co., Ltd., Jianshui, China), and a precision alcohol meter with a division value of 0.1% vol, etc. (Kemei Instrument Co., Ltd., Nantong, China).

### 2.3. Methods

#### 2.3.1. Sample Preparation and Collection

The steps for making sweet wine were as follows:

In the first step of the production process, the rice was soaked and washed. After 1000 g of white glutinous rice was washed three times, it was added to cold water containing 2.5 to 3 times the mass of glutinous rice, which was soaked for 12 h until it was crushed by hand. At this point, the glutinous rice was dripping to 1.5 to 2 times its original size. The soaked glutinous rice was rinsed with tap water and drained; in the second step, the glutinous rice was steamed. A layer of gauze was placed on the steaming tray, the glutinous rice was spread evenly on the gauze, and the cotton was cooked in the induction cooker for 60 min until “cooked but not mushy, transparent but not rotten, loose and easy to disperse, and uniform”. The cooked glutinous rice swelled to about 3–3.5 times its original size. In the third step, the rice was poured to cool. The steamed glutinous rice was rinsed with cold boiled water, and 30% of the dry mass of the glutinous rice was sprinkled to cool it to approximately 30 °C while the rice grains were loosened. The cooling speed was as fast as possible. In step 4, the yeast was mixed together. According to previous studies, Angel wine yeast contains both root mold and yeast, which are good fermentation agents. As a fermentation agent for SFR, it is the optimal choice [38]. In total, 4% of the dry mass of glutinous rice was added to the amount of wine and mixed evenly into the cooled rice; then, the mixture of glutinous rice was weighed to obtain about 3500 g, and then about 280 g of mixed glutinous rice was placed into a glass jar with a capacity of 380 g and divided into an average of 12 cans of packaging and three bottles as replicates for each time slot.

**Figure 1:**
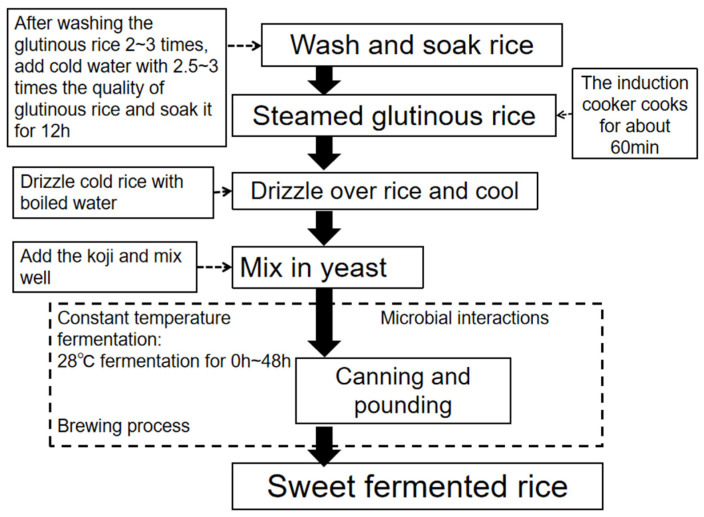
Flowchart of making liquefied wine.

In step 5, a small round concave nest was pounded into the middle of the rice mixed with the starter, which was then covered and sealed in a constant temperature incubator at 28 °C fermentation for 48 h. Please see the production flow chart (Figure 1).

After production, the samples were numbered and prepared to be sensorily evaluated by specialized tasters inside a winery, as detailed in Souza-Coutinho et al. [39]. Samples were taken at 0, 24, 36, and 43 h of fermentation based on the scoring results, and three different samples from each period were placed on a sterile bench to mix the sweet wine grains with a sterile spoon. After mixing the samples, 100 g were scooped into sterile and enzyme-free centrifuge tubes to be capped. An additional 50 g was collected for the determination of pH, total sugars, total acids, and alcohol [40]. After mixing, samples were taken from the bottom of the container at different stages of the vinification process, and 0.5 L of samples were taken at each stage of the vinification process for the experiment, as described by Liu et al. [41]. Also, 500 mL of fermentation mash was collected for physicochemical property analysis. It was then transported back to the laboratory in a cryopreservation box and stored at −80 °C in a refrigerator for backup. They were named A0, A24, A36, and A43.

#### 2.3.2. Evaluation of Flavor and Determination of Physicochemical Properties

For the flavor evaluation, a certain number of samples were removed and numbered. Seven tasters with a good sense of smell and taste were invited to the winery to taste and score these flavors. Here, we required the tasters to be in good health, free from bad habits such as smoking and alcoholism, have robust discrimination and sensitivity to all senses, and not to have consumed spicy and other stimulating foods within 24 h [42]. Tasters are required to follow ISO 4121-2003 “Sensory Analysis—Guidelines for the Use of Quantitative Response Scales” for standardized evaluation [23]. With reference to the usage guidelines, we created a relative reference standard applicable to this product, i.e., to evaluate the appearance, aroma, and taste of the product with scores of 10 and 30, respectively. The scoring reference standard is shown in Table 1.

This study used direct titration to determine the total sugar content in sweet wine, the total acid determination method to determine the pH, total acid content, and total sugar content in sweet wine, and the alcohol content measurement method to determine the alcohol content in wine. Specifically, the total sugar content was determined via the GB 5009.7-2016 National Food Safety Standard Direct Titration Method. The principle is that under heating conditions, methylene blue is used as an indicator, and the sample solution is titrated. The reducing sugar in the sample solution reacts with potassium sodium copper tartrate to produce a red cuprous oxide precipitate. This precipitate forms a soluble colorless complex with potassium ferrocyanide. After all the divalent copper is reduced, a slight excess of reducing sugar reduces methylene blue, and the solution changes from blue to colorless, which is the endpoint of titration. The reduced sugar content can be calculated based on the consumption of the sample solution. The total acid content was determined according to the GB 12456-2021 National Food Safety Standard. The principle is to use an alkaline solution to titrate the acid in the test solution according to the principle of acid–base neutralization. Phenolphthalein is used as an indicator to determine the titration endpoint and the total acid content in the food is calculated based on the consumption of an alkaline solution. The alcohol content was determined via the alcohol meter method in GB 5009.225-2023 National Food Safety Standard. The principle is to remove non-volatile substances from the sample by distillation. The alcohol volume fraction was measured using an alcohol meter, and temperature correction was performed by querying the alcohol meter temperature and 20 °C ethanol concentration (alcohol content) conversion table to obtain the ethanol concentration (alcohol content) of the sample at 20 °C.

#### 2.3.3. High-Throughput Sequencing and Sequence Processing Analysis

Total DNA was extracted from each microbial sample via the CTAB method [43]. High-throughput sequencing was performed by Tsingke Biotech Co., Ltd. (Kunming, China). Metagenomic sequencing was utilized, in which sample DNA fragments were sequenced using the Illumina HiSeq X platform in the sequences ITS5 (GGAAGTAAAAGTCGTAACAAGG) and ITS2 (GCTGCGTTCTTCATCGATGC) [44]. The sequences with an average read length of 150 bases and a Q value of 30 were obtained. The sequencing data were analyzed via the Genes Cloud platform (www.genescloud, accessed on 24 October 2023).

DNA fragments were subjected to paired-end sequencing on the Illumina platform. The sequences were denoised and clustered via Vsearch (v2.13.4-linux-x86_64) and cutadapt (v2.3) [45]. After sequence primer fragments were removed via the qitime cutadapt trim-pair, sequences that did not match the primers were discarded.

The Vsearch module was used to assemble, deduplicate, and decode the sequences. The enriched chimeras were filtered via the UNITE database (Release 8.0, https://UNITE.ut.ee, accessed on 25 October 2023) to obtain high-quality chimeras. QIIME2 (classification-learning algorithm) https://github.com (accessed on 29 October 2023). The feature reclassifier was used to annotate the feature series of each operational taxonomic unit (otu) in the naive Bayes classifier [46]. The QIIME2 qiime feature-table rare function was used to set the leveling depth to 95% of the minimum sample sequence size to obtain the final result. The obtained raw data have been uploaded to the BioProject of NCBI with the login number PRJNA:1159902 (URL https://dataview.ncbi.nlm.nih.gov/object/PRJNA1159902?reviewer=dp3jgfslj4n0buqmptq19297h1, accessed on 12 September 2024).

#### 2.3.4. Species Composition Assessment

The taxonomic composition of the samples was analyzed via Krona software (https://github.com/marbl/Krona/wiki, version 2.8.1, accessed on 11 December 2023) [47]. The RGGplot2 package (version 2.2.1) was used to draw a circular ladder tree plot, and the abundance of each group was added to the plot in the form of a pie chart [48]. To further compare the differences in species composition among the samples and show the distribution trend of species abundance in each sample, a species composition heatmap was created via R language (version 4.4.3) and the pheatmap software package (version 1.0.12).

#### 2.3.5. Alpha Diversity and Beta Diversity Assessment

Alpha diversity refers to the diversity within a sample. The commonly used α diversity indices include the Chao1 estimator, Good’s coverage, observed species, Pielou’s evenness, the Shannon index, the Simpson index, etc. [49,50]. The Shannon index combines abundance and evenness to give more weight to rare species. The Simpson index combines abundance and evenness but focuses more on common species.

Alpha diversity analysis was performed via QIIME2 (version 2024.10), R language (version 4.4.3), and the RGGplot2 software package (version 2.2.1). Using the unleveled OTU table, the “qiime diversity alpha-rarefaction” command was used, the minimum leveling depth was set to ten, the minimum sequencing depth was 95% of the sample sequence, and each depth value was adjusted ten times to calculate alpha diversity. The Chi Plot online drawing tool was used to draw the difference map between the groups of diversity index.

Beta diversity refers to the difference between samples or groups and is usually used to analyze whether the difference in microbial composition between two groups is significant. The commonly used beta diversity indices include the Jaccard, Bray‐Curtis, unweighted Uni Frac, and weighted Uni Frac indices [49,51]. The Jaccard index compares the similarities and differences between finite sample sets. The Bray‐Curtis difference is a measurement used to analyze the differences in species composition between regions. Unweighted Uni Frac can detect differences among samples, whereas weighted Uni Frac can further quantitatively detect differences between different lineages.

Principal coordinate analysis (PCoA) and nonmetric multidimensional scaling (NMDS) methods were used to analyze the beta diversity of the samples [52,53]. By default, the UPGMA algorithm was used for cluster analysis [54] on the Bray‐Curtis distance matrix. The ggtree of the R language was used to analyze the relationships between different samples for visualization.

#### 2.3.6. Analysis at the Functional Level

DIAMOND software (version 5.1) was used to compare the nonredundant genes with each functional database, the annotations with e < 1 ×10^−5^ were selected, and the protein with the highest sequence density was screened to obtain functional annotation information. For the alignment result of each sequence, the alignment result with the highest score (one HSP > 60 bits) was selected for subsequent analysis [55]. Based on the comparison results, the relative abundance of different functional levels was calculated (the relative abundance of each functional level is equal to the sum of the relative abundances of the genes annotated at that functional level [55,56,57,58]). From the functional annotation results and gene abundance starting from the table, the gene number table for each sample at each classification level was obtained. For a certain function, the number of genes in a sample fit for the number of genes with an abundance other than 0 among the genes was annotated as this function. Starting from the abundance table at the classification level, we performed statistics on the number of annotated genes, an overview of the relative abundance, a display of the relative abundance, a functional abundance-based analysis of differences within the Anosim group, a comparative analysis of metabolic pathways, Metastat, and LEfSe analysis of the functional differences between the groups. The classification information in each database, combined with the abundance table of genes in each sample, could be used to obtain the relative abundance information of each database at different levels. The relative abundance table of the first level (level A) of each database was used to draw a statistical map at the first level corresponding to each sample. Using R software (version 4.4.3), a histogram was drawn for the functional composition of each sample. Using PICRUSt2 software (https://github.com/picrust/PICRUSt2/wiki, accessed on 1 November 2023), the abundance values of metabolic pathways were obtained, and the generated data were stored in the KEGG pathway database (https://www.genome.jp/kegg/pathway.html, accessed on 1 November 2023), evolutionary genealogy of genes: Non-supervised Orthologous Groups (egg NOG) database and carbohydrate-active enzymes database (CAZy) and compared. The metabolic pathways and the six functions of carbohydrate enzymes were analyzed [55]. Using the R language and the Meta Genomeseq software package (version 3.20), the fit feature model function was used to analyze the distribution of each pathway/group with a zero lognormal model. The data selected in each abundance table were plotted and analyzed for the metabolic pathways and the six functions of carbohydrate enzymes in the species.

## 3. Results

### 3.1. Evaluation of Flavor and Analysis of Physicochemical Properties

**Figure 2:**
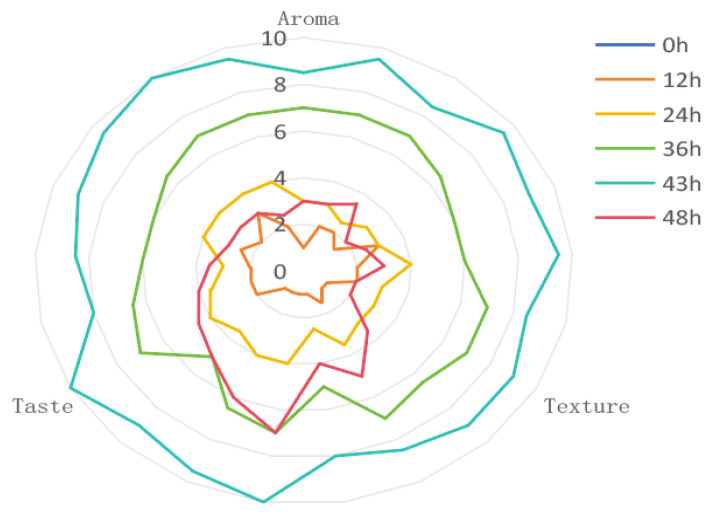
Radar chart of flavor scores of sweet wine brews.

Evaluation is necessary for a fermented beverage flavor. For example, in wines [59], white wines [39], and rice wines [60], studies have shown that the use of sensory evaluation is an indispensable part of the analysis of the quality of various fermented beverages. Xiao et al. [61] also carried out sensory evaluations and instrumental analyses of white wines, and they investigated the correlation between sensory attributes and volatile compounds in cherry wines. The present study was based on the fact that we invited seven professional tasters with good olfactory and gustatory senses in the winery to taste and score these flavors. The results shown in Figure 2 indicate that the SFR was too acidic and old at the end of fermentation up to 48 h, resulting in a low score and poor flavor of the cuvée. The best flavor time was 43 h, which was used only to determine the end of fermentation and was evaluated by looking, smelling, and tasting for aroma, organization, and mouthfeel. This indicates that at this time, the dominant species in microbial activity in SFR is significantly active, and consequently, the microbial community gradually decreases in variety.

The samples for this experiment were homemade under fixed equipment conditions, and according to the previous scoring results, we tasted the SFR at the end of fermentation, after 48 h. Its acidity was too high and not fresh, resulting in poor taste and flavor of the sweet wine brew. Therefore, samples were taken when the SFR was fermented at 0 h, 24 h, 36 h, and 43 h and tested for pH, total acid content (g/(100 g SFR)), total sugar content (°Brix), and alcohol content (% *v*/*v*). Before fermentation (A0), the pH was 6.19, and total acid, total sugar, and alcohol contents were not detected, indicating that fermentation had not yet started and that the microbial activities had not changed. During fermentation (A24 and A36), the pH of A24 was 3.86, the total acid content was 0.17, the total sugar content was 10, and the alcohol content was not detected; the pH of A36 was 3.89, the total acid content was 0.24, the total sugar content was 21, and the alcohol content was not detected. When fermentation ended (A43), the pH was 3.75, the total acid content was 0.34, the total sugar content was 33, and the alcohol content was 3.5% *v*/*v*., indicating that the fermentation process had entered the alcoholic fermentation stage, and that fermentation had ended (Table 2).

### 3.2. Species Composition Analysis

This study used Krona analysis to examine the dynamic changes in the fungal and bacterial communities in SFR starter cultures during different periods. From inside to outside, the Krona (https://github.com/marbl/Krona/wiki, accessed on 11 December 2023) circles represent the seven classification levels, including kingdom, phylum, class, order, family, genus, and species. The sector size reflects the relative abundance of different taxa and has specific values. Different colors represent various taxa. Using A0 as a control (CK), high-throughput sequencing analysis was performed on the fermentation samples, including 1199 species under 334 genera for A0, 1557 species under 481 genera for A24, 1349 species under 438 genera for A36, and 1254 species under 409 genera for A43.

The analysis results for the abundance of the dominant bacterial genera in each sample revealed that *Pseudomonas* and *Enterobacter* were the main genera among bacteria in sample A0, accounting for 38% and 10%, respectively; *Rhizopus* was the main genus among fungi, and *Wickerhamomyces* accounted for 1%. In sample A24, the dominant bacterial genera remained unchanged and accounted for 20% of the total. Among fungi, the dominant genera increased to 73%, with *Rhizopus* and *Wickerhamomyces* accounting for 38% and 27%, respectively. Moreover, there was an increase in other fungi, such as *Parasitella* and *Cyberlindnera*. The dominant bacterial genera and species in sample A36 remained unchanged; the dominant fungal genus, *Rhizopus*, increased to 56%, and the number was more prominent with the relative number of *Wickerhamomyces* decreasing to 15%, accompanied by *Parasitella* and *Mucor*. In sample A43, the dominant fungal genus was *Wickerhamomyces*, which was the dominant genus and accounted for 44%, while *Rhizopus* decreased to 26%; some genera also increased, such as *Candida* and *Cyberlindnera*. Bacteria accounted for 13%, mainly *Pseudomonas*, accompanied by *Acinetobacter*, *Pantoea*, *Staphylococcus*, and *Enterobacter.* The Krona plot of species abundance information analysis results at different stages (A0, A24, A36, A43) for each sample is presented in https://github.com/marbl/Krona/wiki.

**Figure 3:**
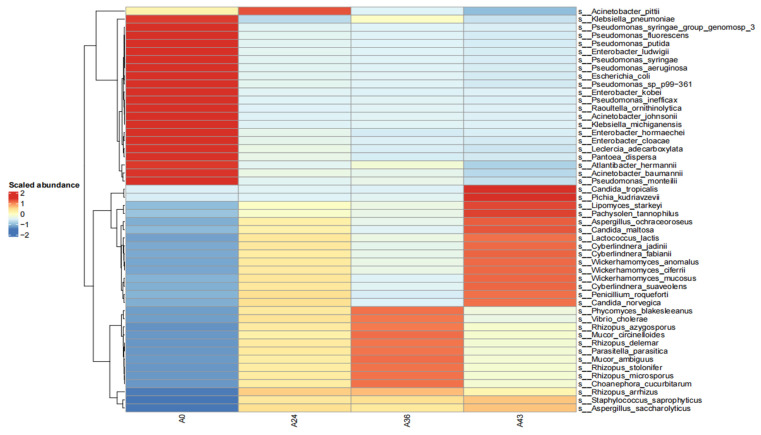
Heat map of the horizontal distribution of microorganisms in each sample. In the figure, the color represents the species abundance; the vertical clustering indicates the similarity of the abundance of different species among the samples, and the closer the distance between two species, the shorter the branch length, which represents that the abundance of these two species is more similar among the samples.

To observe species with high abundance, we first drew a heatmap of the abundance data of the top 50 microorganisms with the highest average abundance, which clearly reflected the correlation between bacterial communities in the samples and also clearly displayed the distribution trend of bacterial abundance in each sample, as shown in Figure 3. The relationship between each sample and strain can be seen from the heatmap, with *Wickerhamomyces anomalus* and *Rhizopus delemar* strains being the dominant strains and the variety of strains gradually decreasing. This finding is consistent with the results of the previous species composition analysis.

**Figure 4:**
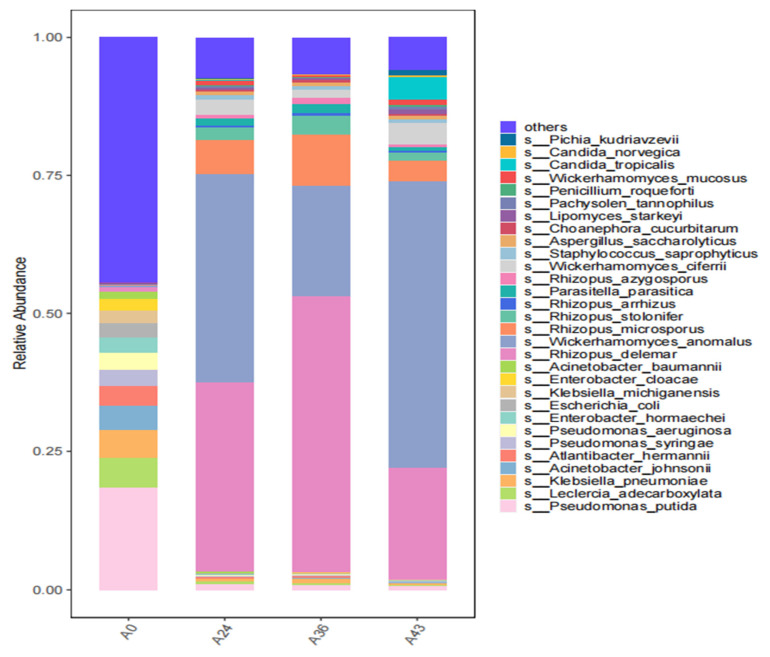
Histogram of species composition in each species landscape. The horizontal axis represents the name of each group in the grouping scheme, and the vertical axis represents the relative abundance of each taxon at a specific taxonomic level.

The top 30 species in terms of richness for each sample were plotted in a histogram presented in Figure 4. The dominant bacteria before fermentation (A0) were *Pseudomonas putida* (abundance value = 28,677.292) and *Leclercia adecarboxylata* (abundance value = 8331.069); at this time, the bacteria were dominant, and fermentation did not start. During fermentation (A24), the number of bacteria decreased, and fungal species, including *W. anomalus* (abundance value = 153,214.117), *R. delemar* (abundance value = 139,764.789), and *Rhizopus microsporus* (abundance value = 25,201.117), were dominant. At this time, the contents of total acids and sugars, which correspond to physicochemical properties, increased. Furthermore, (A36) *Rhizopus* accounted for the largest proportion, with *R. delemar* (abundance value = 209,279.219) as the dominant species, followed by *W. anomalus* (abundance value = 84,353.034) as the dominant yeast species and then *R. microsporus* (abundance value= 38,729.333) and *Rhizopus tolonifer* (abundance value = 14,349.716). As fermentation continued, at the later fermentation stage, (A43) yeast species became dominant, especially *W. anomalus* (abundance value = 241,660.954) and *R. delemar* (abundance value = 94,697.099) as the dominant fungi, followed by *Candida tropicalis (abundance value =* 18,970.787) and *Wickerhamomyces ciferrii* (abundance value = 18,219.521). At this stage, the alcohol content was 3.5% (*v*/*v*), and the total sugar content reached a high level of 33 (°Brix), confirming that *W. anomalus* and *R. delemar* were the main species responsible for successful SFR fermentation and that *C. tropicalis*, *W. ciferrii*, and *R.microsporus* were the following main species involved in fermentation. The whole fermentation process is a chain process accompanied by saccharification and complete fermentation so that the total sugar, total acid, and alcohol contents in SFR are in harmony with each other, providing the best taste and flavor.

### 3.3. Alpha Diversity and Beta Diversity Analysis

**Figure 5:**
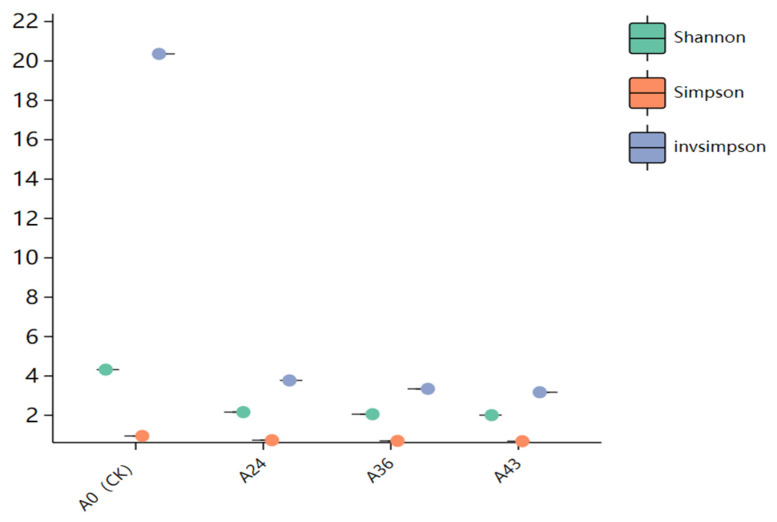
α diversity indices of the samples.

The Shannon, Simpson, and invsimpson indices were calculated for α diversity index analysis to characterize the α diversity of the microbiota in each starting sample. The Shannon index, Simpson index, and invsimpson index reflect the diversity of microbial communities, with higher scores indicating higher diversity and lower scores indicating lower diversity. During SFR fermentation, sample A0 presented the highest species diversity, and A43 presented the lowest (Figure 5). Among the four fermentation broth samples, A0 (CK), A24, A36, and A43, the Shannon index was highest at A0 and gradually decreased over time. The Simpson and invsimpson indices also showed the same trend as the Shannon index did, indicating that species diversity gradually declined with the continuation of natural fermentation.

**Figure 6:**
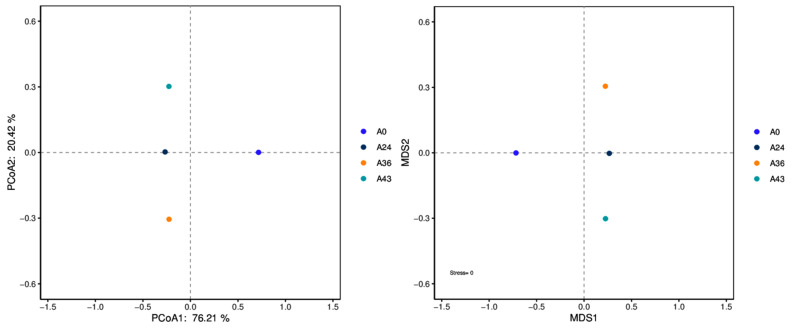
Diagrams of PCoA and NMDS.

The results of the β diversity index analysis are shown in Figure 6. When A0 was used as the sample control (CK), the microbial community composition of the A0 sample was very different from that of the A24, A36, and A43 samples, and the microbial community structure was quite different. The microbial community structure in the CK samples was rich, and the microbial community structure was significantly different from that in the A24, A36, and A43 samples. The identical distances between A24, A36, and A43 indicate that the microbial community structures are similar.

**Figure 7:**
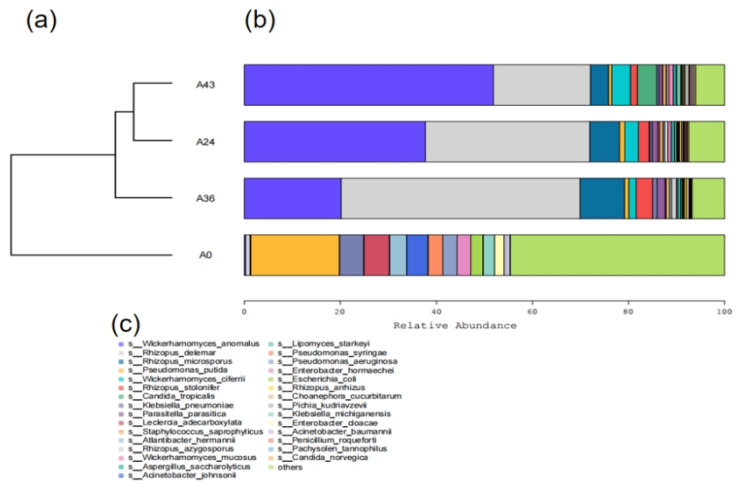
Cluster analysis of species richness in each sample (**a**), stacked map of species composition (**b**), and annotations indicated by each color in the stacked map of species composition (**c**).

Hierarchical clustering tree analysis was performed based on the similarity between the samples Figure 7. Figure 7a shows a hierarchical clustering tree diagram in which samples are clustered based on their similarity to each other. The shorter the branch lengths between samples, the higher the similarity. Figure 7b is a stacked histogram showing the 30 most abundant species. In Figure 7c, each color in the stacked plot of species composition indicates annotation. The species composition distance of the A24 and A43 samples was the closest, indicating that the species composition between the two samples was the most similar. Notably, the microbial community structure exhibited variability and similarity in SFR at different time points. According to hierarchical clustering tree analysis corresponding to the stacked histograms of the 30 most abundant species, *R. delemar* accounted for a large proportion in samples A24 and A36, whereas *W. anomalus* accounted for the highest proportion in the A43 sample. There was a significant difference between A24 and A36.

On the other hand, the species composition of the A0 sample was the farthest from that of the other samples, indicating that the species composition of A0 was different from that of the other samples. The difference between them was significant. This result was consistent with the alpha diversity analysis, PCoA, and NMDS results.

### 3.4. Functional Level Analysis

**Figure 8:**
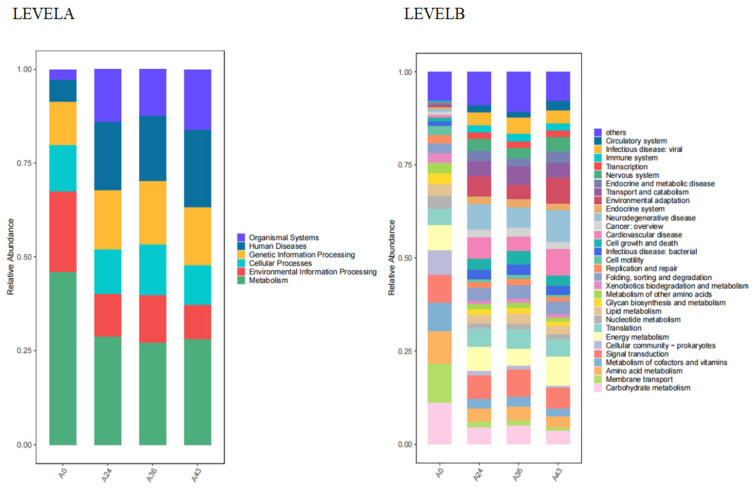
KEGG comparison of the primary (LEVELA) and secondary metabolic pathways (LEVELB) of each sample.

The functional metabolic ability of the microbial community was inferred based on the composition of the 16S rRNA gene metagenomic data for different fermentation agents [62]. In the first-level KEGG metabolic pathways, the functional genes enriched with SFR fermentation agents were associated with cellular processes, environmental information processing, genetic information processing, human diseases, metabolism, and organism systems. The abundances of the four samples were compared, and the results are shown in Figure 8 (LEVELA). The vertical axis represents the average value of the functional pathway abundance in the selected samples, and the horizontal axis represents the sample names for each period. Different colors represent different metabolic pathways. Metabolism-related pathways were significantly enriched in most samples, especially A0. While the abundance of genes related to organism systems in the A0 sample was relatively low, metabolic pathways decreased with increasing fermentation time and then stabilized, while the metabolic pathways of the organisms in A24, A36, and A43 were higher than those in the A0 fermentation samples.

**Figure A1:**
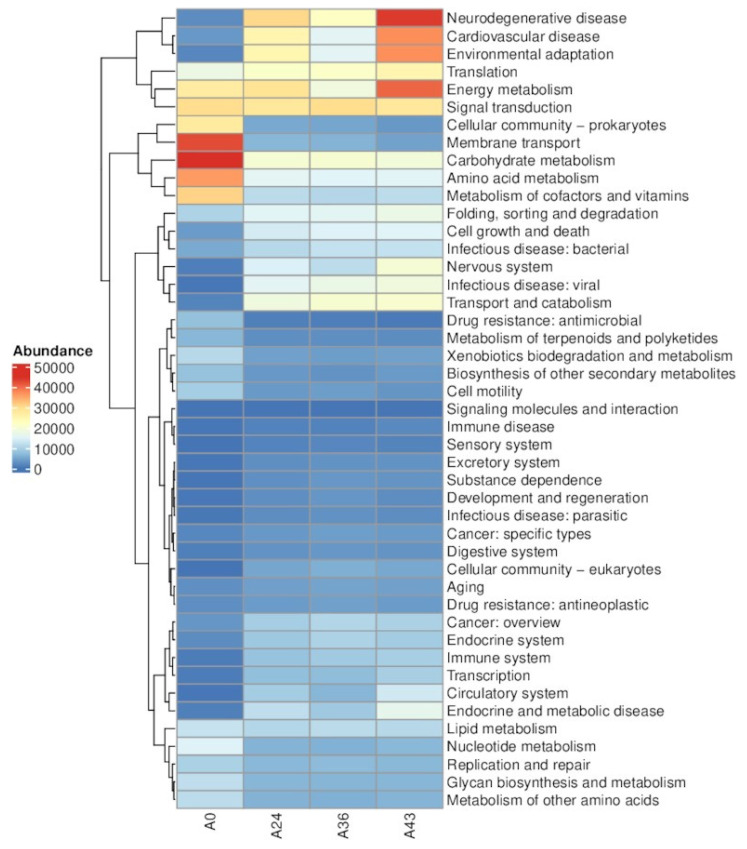
Heat map of the distribution of metabolic pathways in each sample (Level B).

To further explore the causes of these functional changes, we evaluated the secondary pathways involved in the metabolism of SFR. There were 46 secondary metabolic pathways involved, and the corresponding abundance values for each secondary pathway are shown in Table A1 in Appendix A. The results of the analysis of secondary metabolic pathways associated with increased expression levels are shown in Figure 8 (LEVELB). Among the level 2 KEGG pathway categories, the most abundant metabolic ability was energy generation and conversion, followed by inorganic ion transport and metabolism, carbohydrate transport, amino acid transport and metabolism, nucleotide transport and metabolism, and lipid transport and metabolism. Inferred carbohydrate transport, amino acid transport, and metabolism were prominent in the A0 sample; energy generation and conversion metabolic pathways were outstanding in the A24 sample; posttranslational modification, protein turnover, and partner metabolic pathways were prominent in the A36 sample; and chromatin structure and dynamics were prominent in sample A43. A heatmap analysis was subsequently performed on the secondary metabolic pathways with relatively high expression levels (Appendix B, Figure A1). The results were consistent with the abundance map.

**Figure 9:**
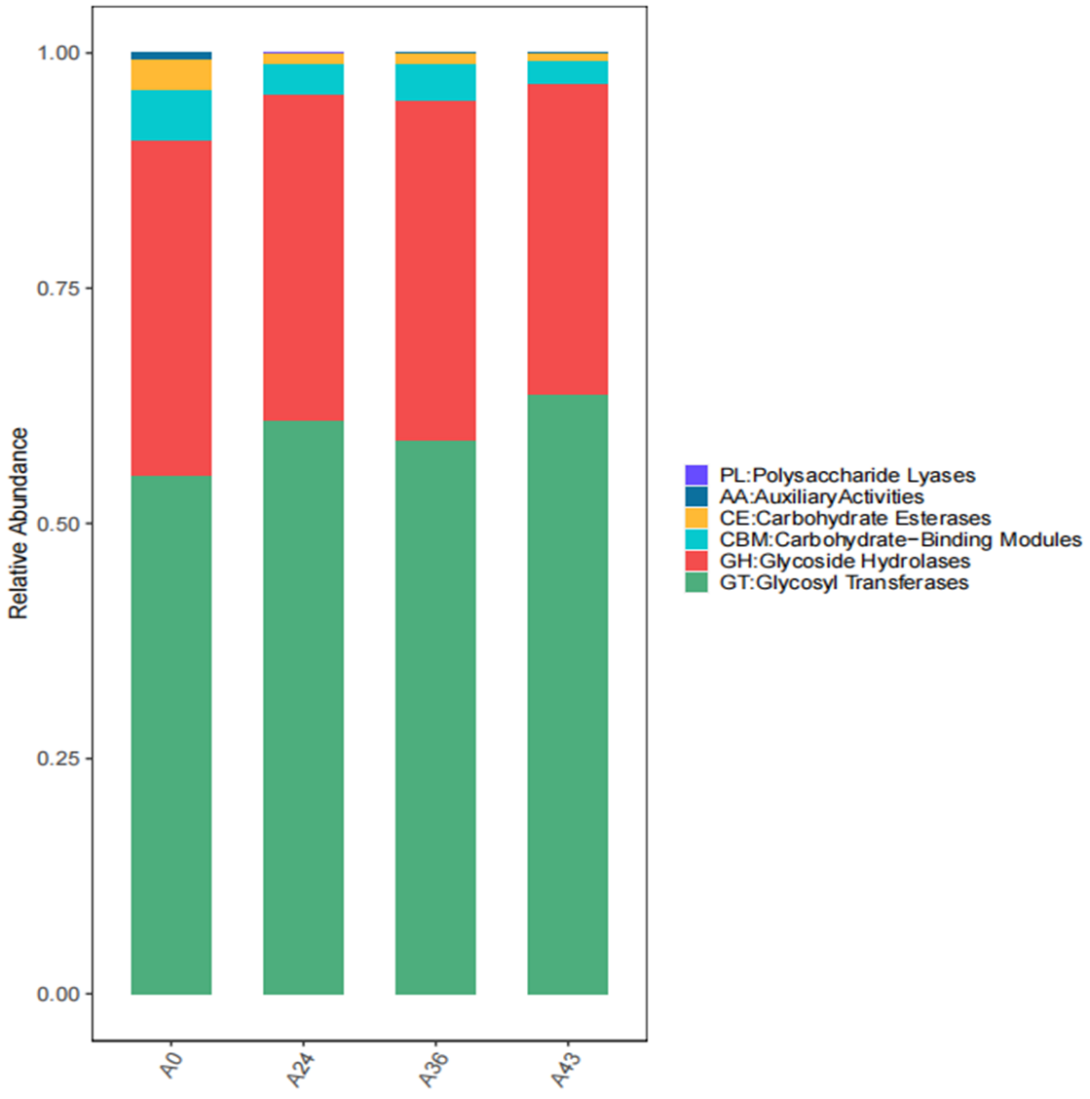
Abundance values of six functions of CAZy carbohydrases.

The functions of six carbohydrate enzymes in the carbohydrate-active enzyme database (CAZy), including glycoside hydrolases (GHs), glycosyltransferases (GTs), polysaccharide lysis (PLs), carbohydrate lipases (CEs), carbohydrate-binding modules (CBMs), and accessory module enzymes (AAs), were analyzed in each sample. The plot of functional abundance obtained above is shown in Figure 9. The three functions of GTs, GHs, and CBMs were used throughout the fermentation process and played dominant roles.

**Figure 10:**
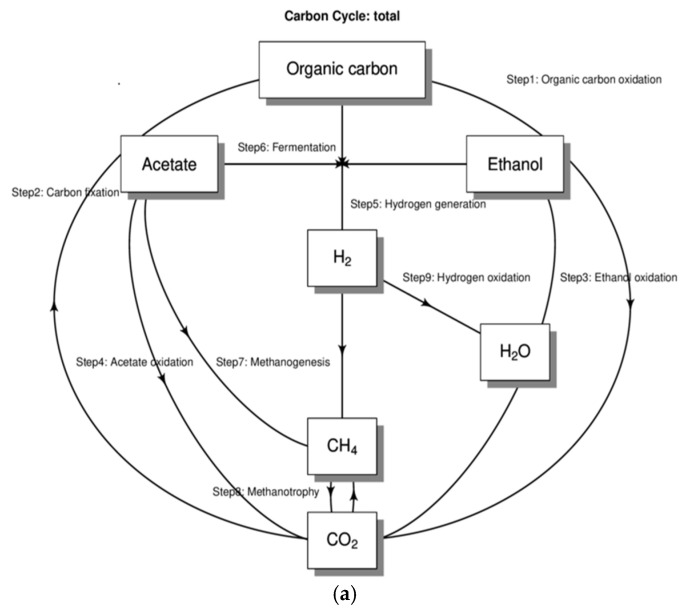

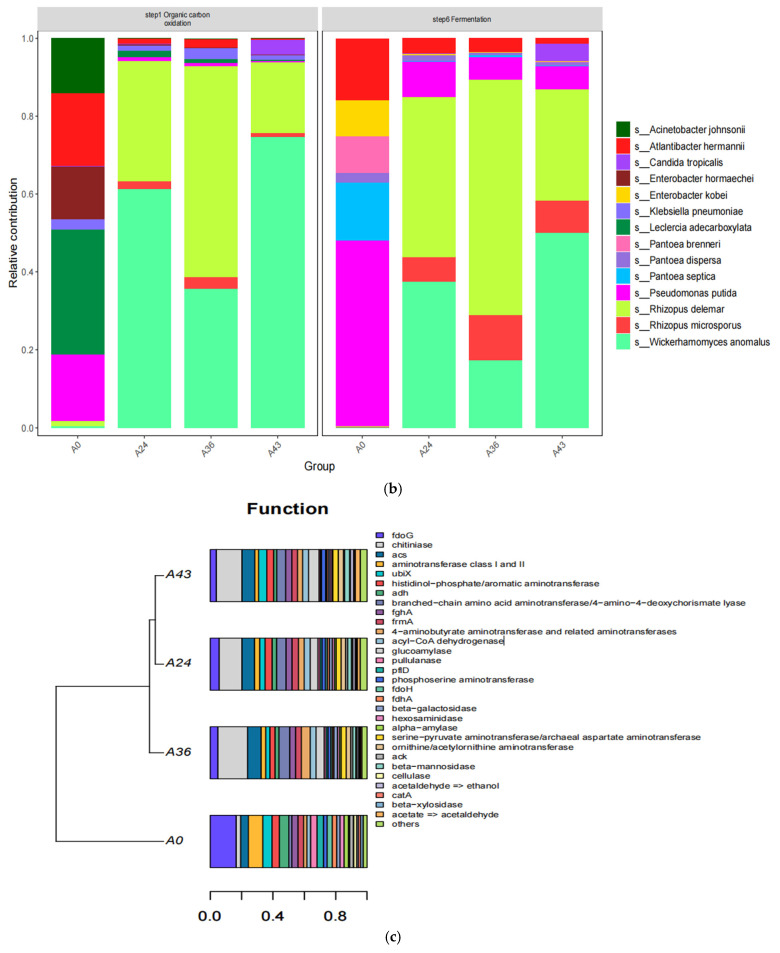
(**a**) Schematic diagram of the carbon cycle pathway. (**b**) Contribution map of species to genes. (**c**) Bray cluster analysis and stack plot of functional genes.

The above information was extracted from the functional annotation results in the database. Based on the TPM (transcript per mill) abundance calculation method, we used TPM as an indicator to calculate the relative abundance of genes or transcripts, which facilitated the comparison of the expression levels of different genes. The calculation formula was the expression value of each gene divided by the gene length and then multiplied by 10^6^, and functional composition analysis, difference analysis, and correlation analysis were performed. The carbon cycle pathway was analyzed through biogeochemical cycles (Figure 10a). There were a total of nine steps. The major species composition of function-specific genes in the four periods was then analyzed. The major species composition of specific functional genes was then analyzed for four periods. Significant microbiota contributions occurred mainly in step 1, the oxidation of organic carbon, and step 6. The most significant species contributing to functional genes in A0 was *P. Septica*. The most significant contributors to functional genes from A24 to A43 were *R. delemar*, *R. microsporus*, *P. putida*, and *W. anomalus* during the fermentation process (Figure 10b), in agreement with the results of Krona’s plot (Figure 10b).

Based on the relative abundance information of functional genes in all samples (the top 30 functional genes in terms of overall abundance were selected here), we calculated the Bray distance between the samples and then performed hierarchical clustering based on the Bray distance. In this way, we could determine similarity. Moreover, for ease of understanding, the gene classification heap-cum-diagram and the clustering results of the Bray distance are shown together. The results (Figure 10c) showed clustering of A24 and A43, indicating that the functional genes during these two periods were chitinase, acetyl-CoA synthase (ACS), and aminotransferase class I and class II genes. The carbohydrate-degrading enzyme FdoG accounted for a large proportion of the A0 genes, and the number of ACS genes was greater in A36. The separate clustering indicated that the functional genes in these two periods differed from those in the other two periods.

## 4. Discussion

Flavor evaluation is necessary to analyze a fermented beverage, which not only helps producers reduce consumption by the time it takes to produce the optimal taste but also provides insights into consumer preferences for rice wines. For example, Lee et al. [63] used sensory data to understand consumer preferences for rice wine. However, other studies investigated the relationship between sensory attributes and volatile compounds with the acceptance of rice wine (Yakju) among young consumers [60], as well as using sensory evaluations and chemical analyses to characterize the sensory attributes of Korean rice wine (maguro) [64]. Similarly, some researchers have studied the fermentation process of black glutinous rice liqueur through flavor evaluation. These studies have shown to be an indispensable part of the use of sensory evaluation to analyze the quality of various fermented beverages [23]. In this study, based on the observation, evaluation, and monitoring of fermentation, the SFR was too acidic and old at the end of the fermentation up to 48 h, which led to the poor flavor of the brews, and the best flavor period was 43 h according to the scoring radar chart, which was only used to determine the end of the fermentation in this study. According to the analysis of physical and chemical properties, with increasing fermentation time, the pH value in the fermentation environment gradually decreased, the total acid content was not detected until the final measurement of 0.34 g/ (100 g SFR), the total sugar content was not detected until the final measurement of 33 (°Brix), and the alcohol content was not detected until the final measurement of 3.5% (*v*/*v*), showing an increasing trend. This is because as fermentation progresses, the mold and yeast contained in koji undergo glycolysis, fermenting the sugar contained in sweet wine and producing alcohol [65]. Its acidity is mainly due to organic acids produced by various microorganisms involved in metabolism. The actions of different enzymes, which can present a refreshing and pleasant acidity, can promote the plumpness and balance of sweet winemaking, thus enhancing the overall taste and flavor [66]. During fermentation, some low-molecular-weight sugars are consumed by microorganisms, which increases the organic acid content [67]. In this study, the detection time before fermentation (A0) was 0 h, there were almost no fermentable sugars in the raw materials, total acid and total sugar contents were not detected, and the degree of alcohol was also not detected. At 24 h of fermentation (A24), total acid and total sugar contents gradually increased with increasing fermentation time, which may be related to the gradual decomposition of other sugars. This finding also indicates that microorganisms became active and consumed sugars to generate energy. The gradual increase in total acid content (from 0.17 to 0.34) also confirmed the increase in microbial activity, especially the activity of acid-producing fungi such as *W. anomalus*, *R. delemar*, and *R. microsporus* strengthening. These microorganisms can produce acid through sugar metabolism during fermentation. The continuous increase in acidity was consistent with previous study results [68]. As the main fungal genus involved in the entire fermentation process, *R. delemar* and *R.microsporus* could completely convert the starch in glutinous rice into fermentable sugars, indicating that the existence of *Rhizopus* was inseparable from the increase in sugar content. At the end of fermentation (A43), the alcohol content was 3.5% (*v*/*v*), indicating that the fermentation process began to enter the alcoholic fermentation stage. The alcohol level was not detected until the end of fermentation, possibly because the activity of yeast and mold was low at 0 h, 24 h, and 36 h of fermentation. The production of alcohol marks the beginning of the conversion of sugars into alcohol and carbon dioxide. This stage is a typical feature of traditional alcohol fermentation [69,70]. The detection of alcohol content at the end of fermentation may be related to the activity of yeast and root mold during the fermentation process (A24 and A36). Rhizopus can hydrolyze starch to obtain sugar, while yeast can perform glycolysis to convert glucose into ethanol and produce alcohol, especially *W. anomalus*, *R. delemar*, and *R. microsporus*. Among them, *W. anomalus* can secrete various glycosidases such as β-D-glucosidase, β-D-xylosidase, and α-L-rhamnosidase, and it can produce high yields of ethyl acetate and 2-phenylethanol, which can significantly improve the quality of the wine [71,72,73].

Microorganisms play crucial roles in the formation of Chinese rice wine, including the synthesis of many flavors, textures, and color metabolites [65,74]. The diversity analysis of the microbial community structure and Krona analysis showed significant differences in the microbial community structure at different fermentation stages, which may be closely related to the changes in nutrients, pH, temperature, and other biotic and abiotic factors in the fermentation environment. Before fermentation (A0), the prime dominant species were *Pseudomonas* and *Enterobacter*. The large diversity of microbial structural composition at this time occurred because the SFR-making process was carried out in an open environment, which contributed to the mixing of various microorganisms from the environment. By the time the fermentation reached 24 h (A24), fungi began to dominate, especially *R*. *delemar*, which had become a dominant bacterium that could utilize the large molecular components in raw materials as a form of nutrient consumption to promote its growth and reproduction [75].

At this time, the diversity of the microbial community’s structural composition decreased due to the decrease in oxygen in the fermentation bottle and the decrease in pH in the environment over time. Some aerobic microorganisms and acid-averse microorganisms inhibit their growth and even die in this environment [76]. By 36 h (A36), a continuous increase in the activity of these dominant fungi, *R. delemar* and *R. microsporus,* was observed. The activity continued to increase, fully converting starch into fermentable sugars to prepare for the next stage of alcohol production [17]. The diversity of the microbial community’s structural composition continuously decreased. By 43 h (A43), the yeast became dominant, with *W. anomalus* exhibiting the ability to adapt to the environment and effectively convert substrates during fermentation. Its growth may have been due to the availability of fermentable sugars and certain nutrients (such as proteins and fats) in the later stages of the fermentation process, resulting in the production of alcohol [77]. The production of alcohol and the decrease in pH in the environment led to a gradual decline in microbial community structure with natural fermentation species diversity. This change reflects the adaptive strategy of the microbial community during fermentation, i.e., [78], survival and development under nutrient competition and environmental stress.

This study further analyzed the trends of changes in the enzymes and metabolic pathways that contributed the most to the gene functions among the carbohydrate enzymes during the SFR fermentation process. Carbohydrate enzymes can participate in and regulate key reactions in metabolic pathways. In addition, the major species composition of the function-specific genes in the four different periods was analyzed through carbon cycle pathway tracking. Then, based on the relative abundance information of the functional genes in all the samples (the top 30 functional genes in terms of overall abundance were used here), we calculated the Bray distance between the samples and then performed hierarchical clustering based on the Bray distance. By comparing the abundance of metabolic pathways in different fermentation stages, this study elucidated the importance and change patterns of specific metabolic pathways during fermentation. First, the abundance of the systemic metabolic pathways of organisms in the A24, A36, and A43 samples during fermentation was generally greater than that in the initial fermentation stage (A0), which may reflect the response mechanism of the fermenting microorganisms to environmental stress [79,80]. The activity of metabolic pathways may be related to the response of microorganisms to oxidative stress, nutrient limitation, and other biotic stress conditions in the fermentation environment. The activation of these pathways may be related to various active enzymes and cellular protection mechanisms. These include the activities of three functional enzymes, glycosyltransferases (GTs), glycoside hydrolases (GHs), and carbohydrate-binding modules (CBMs), the functions of antioxidants, and damage repair. Second, metabolic pathways gradually decreased as fermentation time increased [81]. Finally, it was inferred that the functional level analysis supported the significant activity of pathways such as carbohydrate transport, lipid transport and metabolism, and amino acid transport and metabolism in A0 fermentation broth, implying that flavor formation in these samples may be associated with protein and starch metabolism [62,82], thereby increasing the sugar level and detecting the alcohol content in the middle and late stages of fermentation until the end of fermentation. These results indicated that carbohydrate metabolism was the main pathway for microbial growth and energy production in the early fermentation stage. The significant activation of energy production and conversion metabolic pathways in A24 fermentation broth indicates that abnormal *Wickerhamomyces* and *Rhizopus* delbrueckii begin to be active at this stage, promoting increases in the total acid content and total sugar content of 0.17 g/100 g SFR and 10°Brix, respectively. In the A36 fermentation broth, the post-translational modifications, turnover, and role of molecular chaperones of proteins, as well as the significant activation of signal transduction mechanisms in the A43 fermentation broth, indicate an increase in microbially driven nutrient-seeking activity in the sample at this stage, which may enhance microbial signal perception ability through a complex signal network. Therefore, they can better utilize nutrients [83]. According to Bray distance, hierarchical clustering was performed, and the results revealed A24 and A43 clustering, indicating significant differences in the abundance values of functional chitinase and acetyl-CoA synthase (ACS) genes and aminotransferase gene classes I and II during these two periods. There was a significant difference in the carbohydrate-degrading enzyme-encoding gene (FdoG) in A0, and there were more ACS clusters in A36, indicating significant differences in functional genes between these two periods and the other two periods. Moreover, by tracking two steps in the carbon cycle pathway, we found that one is organic carbon oxidation and the other is the fermentation process, while the most functional genes contributing to the most superior species in A0 in these two steps were bacteria such as *P. septica*. The dominant species that contributed the most to the functional genes from A24 to A43 were *R. delemar*, *R. microsporus*, and *W. anomalus*. These results showed that, in yeast, the expression of ACS could reduce the acetate production. This yeast can be further modified to reduce glycerol and (or) increase ethanol production. This type of yeast is used for ethanol production from carbohydrate-containing substrates. Therefore, the role of ACS in carbohydrate enzymes is related to the generation of acetic acid and the glycolytic metabolic pathway [65]. By regulating the generation of acetic acid, it affects the ethanol production capacity of yeast [84]. In summary, the high expression of the above metabolic pathways may be inseparable from the increase in total acid and sugar contents and alcohol content. This finding also indicates that some differences in the functional metabolic abundance of the samples during different periods may be related to the composition of microbial communities in various periods, which might have contributed to the observed variation in volatile compound profiles between these samples.

## 5. Conclusions

This study used high-throughput sequencing technology to analyze and elucidate the dynamic changes in microbial communities, metabolic pathways, and carbohydrate enzyme functions in traditional fermentation broth from SFR. The results of the diversity analysis indicated that over time, fungi became the dominant microbial community, with *Rhizopus* and *Wickerhammyces* being the predominant fungal genera throughout the fermentation process. In the physical and chemical property tests, the total acid and sugar contents increased with increasing fermentation time. Energy production and conversion, carbohydrate transport, and amino acid transport were the most active metabolic pathways in the fermentation process, according to metabolic function analysis. The dominant microorganisms that contribute functional genes to organic carbon oxidation and fermentation processes were tracked in the carbon cycle. Among A24, A36, and A43, the dominant microorganisms that contributed the most functional genes were *R. delemar*, *R. microsporus*, and *W. anomalus*. The advantages of the dominant microorganisms *R. delemar*, *R. microsporus*, and W. anomalus were fully utilized in this study. However, inevitably, other microorganisms may also play a role. These three dominant microorganisms endow SFR with the best flavor during fermentation. It is recommended that these two microorganisms be purified and cultivated as fermentation agents for SFR. The results of this study provide only some insight into the dynamic changes in the microbial population of SFR single samples prepared under fixed conditions and provide a basis for optimizing the physicochemical properties of SFR fermentation broth and controlling the microbial community structure. Moreover, they are crucial for optimizing fermentation conditions and improving product quality and taste.

## Figures and Tables

**Table 1:** Reference standards for sensory evaluation scores of SFR.

Project	Scoring Criteria	Full Marks
Aroma	No aroma or peculiar smell, almost no wine aroma. (0~4) With aroma, no peculiar smell, and insufficient wine aroma. (5~7) Strong aroma, with glutinous rice aroma and light wine aroma. (8~10)	10
Texture	The color is dark or has a different color, and almost no wine seepage. (0~4) The color is uneven, the gloss is insufficient, and the amount of wine seepage is small. (5~7) The color is uniform, the gloss is good, and the wine seepage is normal. (8~10)	10
Taste	Poor taste, poor elasticity, undercooked or soft. (0~4) Average taste, insufficient elasticity, soft or hard. (5~7) Delicious taste, good elasticity, moderate hardness. (8~10)	10

**Table 2:** Detection results of physical and chemical indicators at different stages of SFR.

	Fermentation Cycle (h)	0	24	36	43
Physical and chemical indicators	pH	6.19 ± 0.001	3.86 ± 0.012	3.89 ± 0.010	3.75 ± 0.012
	Total acid (g/100 g SFR)	Not detected	0.17 ± 0.069	0.24 ± 0.054	0.34 ± 0.032
	Total sugar (°Brix)	Not detected	10 ± 0.1	21 ± 0.1	33 ± 0.0
	Alcohol content (% *v*/*v*)	Not detected	Not detected	Not detected	3.50 ± 0.001

## Data Availability

The original contributions presented in this study are included in this article/Supplementary Material, and further inquiries can be directed to the corresponding author.

## Supplementary Materials

The following supporting information can be downloaded at: https://www.mdpi.com/article/10.3390/foods14071121/s1, Krona plot of the species abundance information analysis results in different stages (A0, A24, A36, A43) of each sample.

## Appendix A

**Table A1:** KEGG (LEVEL B) Secondary Pathway Abundance.

LEVELB	A0	A24	A36	A43
Aging	2675.92	4147.91	4599.61	4305.97
Amino acid metabolism	36,127.18	15,742.45	15,160.08	15,219.15
Biosynthesis of other secondary metabolites	7918.37	3532.37	3006.32	3752.05
Cancer: overview	3266.71	9320.59	10626.61	10,000.54
Cancer: specific types	1757.93	3525.66	4051.72	3722.13
Carbohydrate metabolism	44,808.81	19,870.03	20,310.00	18,963.77
Cardiovascular disease	3519.72	24,856.46	15,587.31	37,271.02
Cell growth and death	3845.75	13,802.35	14,767.09	15,110.44
Cell motility	9327.21	3659.88	3987.70	3195.53
Cellular community - eukaryotes	32.51	4845.36	5791.89	5079.86
Cellular community - prokaryotes	27,492.73	5211.41	4854.55	3495.79
Circulatory system	233.32	9238.22	6541.71	13,317.83
Development and regeneration	378.22	2555.40	3315.84	2419.89
Digestive system	1117.77	2947.24	3439.31	3049.20
Drug resistance: antimicrobial	7731.89	1070.23	1025.40	586.61
Drug resistance: antineoplastic	2577.31	3777.11	4376.20	3773.51
Endocrine and metabolic disease	1089.50	11,758.62	8854.67	16,572.83
Endocrine system	2225.45	8709.26	10,016.93	9138.44
Energy metabolism	27,196.95	28,906.73	18,505.78	40,458.00
Environmental adaptation	1848.96	24,725.68	15,581.02	37,154.49
Excretory system	182.32	2618.73	3028.19	2976.12
Folding, sorting and degradation	10,113.97	15,306.08	15,680.13	17,494.11
Glycan biosynthesis and metabolism	11,811.23	6527.91	6448.22	6550.90
Immune disease	276.05	1341.12	1322.05	1965.53
Immune system	807.53	8053.24	8946.12	9502.07
Infectious disease: bacterial	5329.26	10,977.55	12,172.77	12,166.27
Infectious disease: parasitic	330.19	2392.15	2931.09	2349.06
Infectious disease: viral	258.51	15,515.05	17,403.48	18,757.58
Lipid metabolism	12,328.97	10,651.60	11,422.98	10,977.24
Membrane transport	42,630.88	6636.62	6189.63	4395.03
Metabolism of cofactors and vitamins	31,123.23	11,405.85	10,693.61	11,623.45
Metabolism of other amino acids	11,489.73	6124.54	5911.47	6325.28
Metabolism of terpenoids and polyketides	6607.23	2529.87	2571.41	2266.63
Nervous system	1007.23	14,527.07	11,521.70	19,930.87
Neurodegenerative disease	2299.48	30,618.01	21,817.52	44,002.95
Nucleotide metabolism	14,738.38	6269.23	5988.87	6694.13
Replication and repair	9893.67	6684.42	6981.63	6661.09
Sensory system	6.81	1482.42	1821.13	1466.52
Signal transduction	30,004.02	28,074.00	29,988.59	28,270.69
Signaling molecules and interaction	0.64	90.78	123.05	92.26
Substance dependence	152.68	2745.73	3428.49	3044.71
Transcription	839.55	7519.82	7282.92	9511.74
Translation	17,578.72	21,123.98	21,335.18	24,807.37
Transport and catabolism	1426.55	18,433.02	20,281.75	20,499.89
Xenobiotics biodegradation and metabolism	10,956.91	4275.83	3958.65	4369.27

**Table A2:** Six functional abundance values of CAZy carbohydrate enzymes.

Category	A0	A24	A36	A43
GT:Glycosyl Transferases	4560.21	4204.55	4017.57	4552.48
GH:Glycoside Hydrolases	2964.22	2396.13	2469.17	2358.73
CBM:Carbohydrate-Binding Modules	443.48	224.88	266.55	174.80
CE:Carbohydrate Esterases	274.82	80.65	77.281	66.236
AA:AuxiliaryActivities	56.36	6.05	4.01	2.29
PL:Polysaccharide Lyases	0.23	0	0	0.03

## References

[1] Yue F., Hao Z., Jing R., Zhang B. (2012). Research on the processing technology of new type fermented waxy Corn. Food RD FRD.

[2] Yang Y., Chen W., Ma R., Xu L. (2011). Analysis and evaluation of nutrients in sweet rice wine. China Brew..

[3] Ren F., Han Z. (2012). Study on the fermentation mechanism of rice wine. China Brew..

[4] Tian Y., Wang W., Jia C., Liu H. (2008). Study on fermentation process of sweetened glutinous rice wine and changes regularity of components during fermentation. Food Sci. Technol..

[5] Yang Y., Zhong H., Yang N., Zhu D., Li J., Yang Z., Yang T. (2022). Effects of the proteins of indica rice and indica waxy rice on the formation of volatiles of sweet rice wine. Int. J. Food Sci. Technol..

[6] Zhong J., Ye X., Fang Z., Xie G., Liao N., Shu J., Liu D. (2012). Determination of biogenic amines in semi-dry and semi-sweet Chinese rice wines from the Shaoxing region. Food Control.

[7] Zheng X., Wang H., Qiao X., Cha Y., Gao L. (2023). Development status of compound glutinous rice wine production technology. Winemaking.

[8] Jeong J.W., Nam P.W., Lee S.J., Lee K.G. (2011). Antioxidant activities of Korean rice wine concentrates. J. Agr. Food Chem..

[9] Peng L., Ai-Lati A., Ji Z., Chen S., Mao J. (2019). Polyphenols extracted from huang jiu have anti-inflammatory activity in lipopolysaccharide stimulated RAW264. 7 cells. RSC Adv..

[10] Lin H., Zhang J., Ni T., Lin N., Meng L., Gao F., Luo H., Liu X., Chi J., Guo H. (2019). Yellow wine polyphenolic compounds prevents doxorubicin-induced cardiotoxicity through activation of the Nrf2 signalling pathway. J. Cell Mol. Med..

[11] Cai H., Zhang T., Zhang Q., Luo J., Cai C., Mao J. (2018). Microbial diversity and chemical analysis of the starters used in traditional Chinese sweet rice wine. Food Microbiol..

[12] Gammacurta M., Lytra G., Marchal A., Marchand S., Barbe J.C., Moine V., de Revel G. (2018). Influence of lactic acid bacteria strains on ester concentrations in red wines: Specific impact on branched hydroxylated compounds. Food Chem..

[13] Simonen M., Palva I. (1993). Protein secretion in Bacillus species. Microbiol. Rev..

[14] Liu Z., Wang Z., Sun J., Ni L. (2020). The dynamics of volatile compounds and their correlation with the microbial succession during the traditional solid-state fermentation of Gutian Hong Qu glutinous rice wine. Food Microbiol..

[15] Lv X.C., Huang Z.Q., Zhang W., Rao P.F., Ni L. (2012). Identification and characterization of filamentous fungi isolated from fermentation starters for Hong Qu glutinous rice wine brewing. J. Gen. Appl. Microbiol..

[16] Lv X.C., Cai Q.Q., Ke X.X., Chen F., Rao P.F., Ni L. (2015). Characterization of fungal community and dynamics during the traditional brewing of Wuyi Hong Qu glutinous rice wine by means of multiple culture-independent methods. Food Control.

[17] Yang Y., Zhong H., Yang N., Xu S., Yang T. (2022). Quality improvement of sweet rice wine fermented with *Rhizopus delemar* on key aroma compounds content, phenolic composition, and antioxidant capacity compared to *Rhizopus oryzae*. J. Food Sci. Technol..

[18] Liang Z., Lin X., He Z., Su H., Li W., Ren X. (2020). Amino acid and microbial community dynamics during the fermentation of Hong Qu glutinous rice wine. Food Microbiol..

[19] Su Y., Zhao S.M. (2014). Research progress of process technology and quality characteristics of sweet rice wine. China Brew..

[20] Yang Y., Xia Y., Wang G., Yu J., Ai L. (2017). Effect of mixed yeast starter on volatile flavor compounds in Chinese rice wine during different brewing stages. LWT.

[21] Aleksandar P.V., Lisov N., Čakar U.D., Marković N., Matijašević S., Cvejić J.M., Atanacković M., Gojković-Bukarica L. (2019). The effects of *Prokupac* variety clones and vinification method on the quantity of resveratrol in wine. Food Feed. Res..

[22] Zeng Q., Song Y., Liu Z., Xie W., Chen Y., Liang S., Yang Y., Liu F., Song L. (2020). Research on the development and nutritional value of sweet wine Brewing. J. Foshan Univ. Sci. Technol..

[23] Xiong J., Xiang Q., Yan X., Wu J. (2023). Changes in flavor, taste, and quality during the fermentation process of black glutinous rice sweet wine. China Brew..

[24] Lu Y., Peng Y., Zhang Y., Liu S., Cui L. (2021). Optimization of process parameters for fermented sweet osmanthus wine. Agric. Prod. Process..

[25] Lv X.C., Weng X., Zhang W., Rao P.F., Ni L. (2012). Microbial diversity of traditional fermentation starters for Hong Qu glutinous rice wine as determined by PCR-mediated DGGE. Food Control.

[26] Tang Q., He G., Huang J., Wu C., Jin Y., Zhou R. (2019). Characterizing relationship of microbial diversity and metabolite in Sichuan xiao qu. Front. Microbiol..

[27] Lv X.C., Jiang Y.J., Liu J., Guo W.L., Liu Z.B., Zhang W., Rao P.F. (2017). Evaluation of different PCR primers for denaturing gradient gel electrophoresis (DGGE) analysis of fungal community structure in traditional fermentation starters used for Hong Qu glutinous rice wine. Int. J. Food Microbiol..

[28] Lv X.C., Chen Z.C., Jia R.B., Liu Z.B., Zhang W., Chen S.J., Rao P.F., Ni L. (2015). Microbial community structure and dynamics during the traditional brewing of Fuzhou Hong Qu glutinous rice wine as determined by culture-dependent and culture-independent techniques. Food Control.

[29] Liu M., Tang Y., Zhao K., Liu Y., Guo X., Ren D., Yao W., Tian X., Gu Y., Yi B. (2017). Determination of the fungal community of pit mud in fermentation cellars for Chinese strong-flavor liquor, using DGGE and Illumina Mi Seq sequencing. Food Res. Int..

[30] Cocolin L., Campolongo S., Alessandria V., Dolci P., Rantsiou K. (2011). Culture independent analyses and wine fermentation: An overview of achievements 10 years after first application. Ann. Microbiol..

[31] Prakitchaiwattana C.J., Fleet G.H., Heard G.M. (2004). Application and evaluation of denaturing gradient gel electrophoresis to analyse the yeast ecology of wine grapes. FEMS Yeast Res..

[32] Liu Z., Li J., Wei B., Huang T., Xiao Y., Peng Z., Xie M., Xiong T. (2019). Bacterial community and composition in Jiang-shui and Suan-cai revealed by high-throughput sequencing of 16S rRNA. Int. J. Food Microbiol..

[33] Luo F., Yang Z., Zhong K., Huang C., Yu Z., Peng Z., Wu Y., Bu Q., Gao H. (2021). Effects of *Bacillus megaterium* L222 on quality and bacterial diversity of Sichuan paocai. Food Res. Int..

[34] Zhao G., Liu C., Hadiatullah H., Yao Y., Lu F. (2021). Effect of *Hericium erinaceus* on bacterial diversity and volatile flavor changes of soy sauce. LWT.

[35] Zhang Z., Gao Y., Zhao W., Liu X., Zhang H. (2022). Analysis of fungal dynamic changes in the natural fermentation broth of *‘Hongyang’* kiwifruit. PeerJ.

[36] Zhao W., Zhang Z., Gao Y., Liu X., Du C., Ma F., Wang S., Shi W., Yang Y., Zhang H. (2022). Fungal dynamic changes in naturally fermented ‘Kyoho’ grape juice. Arch. Microbiol..

[37] Zou J., Chen X., Wang C., Liu Y., Li M., Pan X., Chang X. (2023). Microbial communities and correlation between microbiota and volatile compounds in fermentation starters of Chinese sweet rice wine from different regions. Foods.

[38] Sun H., Liu G., Zhao W., Bai W., Liang J. (2024). Effects of different enzymes and yeast combinations on the quality of rice flavor Baijiu. China Brew..

[39] Souza-Coutinho M., Brasil R., Souza C., Sousa P., Malfeito-Ferreira M. (2020). Consumers associate high-quality (fine) wines with complexity, persistence, and unpleasant emotional responses. Foods.

[40] Böhmer M., Smoľak D., Ženišová K., Čaplová Z., Pangallo D., Puškárová A., Bučková M., Cabicarová T., Budiš J., Šoltýs K. (2020). Comparison of microbial diversity during fermentation of two different wines. FEMS Microbiol. Lett..

[41] Liu A., Yang X., Guo Q., Li B., Zheng Y., Shi Y., Zhu L. (2022). Microbial communities and flavor compounds during the fermentation of traditional Hong Qu glutinous rice wine. Foods.

[42] Yuan P., Zhu S., Wang X., Wang X., Liu J., Liu S., Liu J., Liu Y., Fan G., Duan S. (2015). Application of intelligent sensory technology in engineering rice research. Food Ferment. Ind..

[43] Zhang N.N., Xu Q., Li H.Y. (2023). Comparative study of DNA extraction methods for microbial genomes in fermented bean curd. China Brew..

[44] Kumar G., Eble J.E., Gaither M.R. (2020). A practical guide to sample preservation and pre-PCR processing of aquatic environmental DNA. Mol. Ecol. Resour..

[45] Rognes T., Flouri T., Nichols B., Quince C., Mahé F. (2016). VSEARCH: A versatile open source tool for metagenomics. PeerJ.

[46] Elolimy A., Alharthi A., Zeineldin M., Parys C., Loor J. (2020). Residual feed intake divergence during the preweaning period is associated with unique hindgut microbiome and metabolome profiles in neonatal Holstein heifer calves. J. Anim. Sci. Biotechnol..

[47] Ondov B.D., Bergman N.H., Phillippy A.M. (2011). Interactive metagenomic visualization in a Web browser. BMC Bioinform..

[48] Steenwyk J.L., Rokas A. (2021). Ggpubfigs: Colorblind-friendly color palettes and ggplot2 graphic system extensions for publication-quality scientific figures. Microbiol. Resour. Ann..

[49] Chao A., Ricotta C. (2019). Quantifying evenness and linking it to diversity, beta diversity, and similarity. Ecology.

[50] Liu K., Zhang Y., Li Q., Li H., Long D., Yan S., Huang W., Long R., Huang X. (2020). Ethnic differences shape the alpha but not beta diversity of gut microbiota from school children in the absence of environmental differences. Microorganisms.

[51] Lozupone C.A., Hamady M., Kelley S.T., Knight R. (2007). Quantitative and qualitative β diversity measures lead to different insights into factors that structure microbial communities. App. Environ..

[52] Ramette A. (2007). Multivariate analyses in microbial ecology. FEMS Microbiol. Ecol..

[53] Zhang J. (2011). Quantitative Ecology.

[54] Bray J.R., Curtis J.T. (1957). An ordination of the upland forest communities of southern Wisconsin. Ecol. Monogr..

[55] Bäckhed F., Roswall J., Peng Y., Kristiansen K., Dahlgren J., Wang J. (2015). Dynamics and stabilization of the human gut microbiome during the first year of life. Cell Host Microbe.

[56] Qin J., Li Y., Cai Z., LI S., Zhu J., Zhang F., Liang S., Zhang W., Guan Y., Shen D. (2012). A metagenome-wide association study of gut microbiota in type 2 diabetes. Nature.

[57] Karlsson F.H., Fåk F., Nookaew I., Tremaroli V., Fagerberg B., Petranovic D., Bäckhed F., Nielsen J. (2012). Symptomatic atherosclerosis is associated with an altered gut metagenome. Nat. Commun..

[58] Karlsson F.H., Tremaroli V., Nookaew I., Bergström G., Behre C.J., Fagerberg B., Nielsen J., Bäckhed F. (2013). Gut metagenome in European women with normal, impaired and diabetic glucose control. Nature.

[59] Coste A., Sousa P., Malfeito-Ferreira M. (2018). Wine tasting based on emotional responses: An expedite approach to distinguish between warm and cool climate dry red wine styles. Food Res. Int..

[60] Heo J., Kwak H.S., Kim M., Kim J.H., Baek H.H., Shin H., Lee Y., Lee S., Kim S.S. (2020). Major sensory attributes and volatile compounds of Korean rice liquor (Yakju) affecting overall acceptance by young consumers. Foods.

[61] Xiao Z., Yu D., Niu Y., Ma N., Zhu J. (2016). Characterization of different aroma-types of Chinese liquors based on their aroma profile by gas chromatography-mass spectrometry and sensory evaluation. Flavour. Fragr. J..

[62] Chen L., Ren L., Li D., Ma X. (2021). Analysis of microbiomes in three traditional starters and volatile components of the Chinese rice wines. Food Sci. Biotechnol..

[63] Lee S.J., Lee K.G. (2008). Understanding consumer preferences for rice wines using sensory data. J. Sci. Food Agric..

[64] Wong B., Owens A., Phillips M., Kam R. (2023). Identifying sensory attributes of Korean rice wine (makgeolli) using sensory evaluation and chemical analysis. J. Food Sci..

[65] Li N., Li Y., Sun Y. (2024). Research progress on regulation mechanism of ethanol in Saccharomyces cerevisiae and breeding of low-yielding strains. Food Ferment. Ind..

[66] Wolfe B.E., Button J.E., Santarelli M., Dutton R.J. (2014). Cheese rind communities provide tractable systems for in situ and in vitro studies of microbial diversity. Cell.

[67] Huang Z.R., Guo W.L., Zhou W.B., Li L., Xu J.X., Hong J.L., Liu H.P., Zeng F., Bai W.D., Liu B. (2019). Microbial communities and volatile metabolites in different traditional fermentation starters used for Hong Qu glutinous rice wine. Food Res. Int..

[68] Tian S., Zeng W., Zhou J., Du G. (2022). Correlation between the microbial community and ethyl carbamate generated during Huzhou rice wine fermentation. Food Res. Int..

[69] Fugelsang K.C., Edwards C.G. (2007). Chapter: Fermentation and Post-Fermentation Processing. Wine Microbiology: Practical Applications and Procedures.

[70] Fleet G.H. (1993). The microorganisms of winemaking-isolation, enumeration and identification. Wine Microbiology and Biotechnology.

[71] Padilla B., Gil J.V., Manzanares P. (2018). Challenges of the non-conventional yeast *Wickerhamomyces anomalus* in winemaking. Fermentation.

[72] Sabel A., Martens S., Petri A., König H., Claus H. (2014). *Wickerhamomyces anomalus* AS1: A new strain with potential to improve wine aroma. Ann. Microbiol..

[73] Sun N., Gao Z., Li S., Chen X., Guo J. (2022). Assessment of chemical constitution and aroma properties of Kiwi wines obtained from pure and mixed fermentation with *Wickerhamomyces anomalus* and *Saccharomyces cerevisiae*. J. Sci. Food Agric..

[74] Englezos V., Jolly N.P., Di Gianvito P., Rantsiou K., Cocolin L. (2022). Microbial interactions in winemaking: Ecological aspects and effect on wine quality. Trends Food Sci. Tech..

[75] Wu J., Ren L., Zhao N., Wu T., Liu R., Sui W., Zhang M. (2022). Solid-state fermentation by *Rhizopus oryzae* improves flavor of wheat bran for application in food. J. Cereal Sci..

[76] Wang L., Wang Y., Wang H., Liu G., Yang F., Jiang H., Xu J., Wang D., Jin T. (2015). Microbial composition analysis of pit mud of Maotai flavor Baijiu. Brew. Technol..

[77] Li H., Wang H., Wu Y., Huang W. (2010). Isolation, identification, and evaluation of brewing performance of natural wine yeast strains. Food Ferment. Ind..

[78] Lu J., San Q.M.G., Tang P., Wang L., Meng T., Liang Q., Zhang L., Feng G., Li C. (2019). Screening of acid resistant yeast strains and their application in the brewing of Maotai flavor Baijiu. Brew. Technol..

[79] Cheng J., Guo W., Hu H. (2013). Isolation and performance determination of an acid resistant Yeast strain. China Brew..

[80] Wang Z., Zhang Y., Meng Q., Chen X., Qi H., Li H., Yan Z. (2020). Screening of high-temperature resistant and high-yield alcohol producing yeast and its application in Daqu production. Brew. Technol..

[81] Li H., Li Z., Qu J., Wang J. (2017). Bacterial diversity in traditional Jiaozi and sourdough revealed by high-throughput sequencing of 16S rRNA amplicons. LWT.

[82] Xiao C., Wang L., Zhang Y.G., Tu T.Y., Wang S.T., Shen C.H., Yuan H.W., Zhong X.Z. (2021). A comparison of microbial communities and volatile compounds in wheat Qu from different geographic locations. LWT.

[83] Zhao X., Xiang F., Tang F., Cai W., Guo Z., Hou Q., Yang X., Song W., Shan C. (2022). Bacterial communities and prediction of microbial metabolic pathway in rice wine Koji from different regions in China. Front. Microbiol..

[84] Yang D., Liu L., Li P. (2019). Analysis of correlation between acetyl coenzyme A and *Saccharomyces cerevisiae* growth, esters production in beer fermentation under top CO_2_ pressure. China Brew..

