# Supplementary material for: Analysis of the Changes in Physicochemical Properties and Microbial Communities During Fermentation of Sweet Fermented Rice
Source: Foods. 2025 Mar 24;14(7):1121. doi: 10.3390/foods14071121 (PMC11988636; doi:10.3390/foods14071121)
Supplement: Supplementary file 1 [file foods-14-01121-s001.zip › Krona plot of species abundance information analysis results in different stages (A0, A24, A36, A43) of each sample.html]

Javascript must be enabled to view this page.

magnitude
magnitudeUnassigned

A0
A24
A36
A43

826778.641562615878.989155001626943.570384999608917.394246
0000

199.208604134.942195162.12277168.822813

070.216073111.54060842.831837

04.2844257.3578822.996257

04.2844257.3578822.996257

04.2844257.3578822.996257

04.2844257.3578822.996257

04.2844257.3578822.996257

065.931648104.18272639.83558

02.257433.3431581.721033

02.257433.3431581.721033

02.257433.3431581.721033

02.257433.3431581.721033

07.01242510.8358014.301422

02.0106773.3299621.05091
05.6504448.6552543.308571

03.6397675.3252922.257661

03.6397675.3252922.257661

01.3619812.1805470.992851

01.3619812.1805470.992851

056.66179390.00376733.813125

056.66179390.00376733.813125
04.0839775.9133792.721791

05.387417.6824513.019091

05.387417.6824513.019091

07.42551916.5674185.117361

07.42551916.5674185.117361

034.83043451.25236420.03851

07.21694714.1067444.828771

02.1205922.6266681.54066

09.27779317.2119178.793198

016.21510217.3070354.875881

04.9344538.5881552.916372

04.9344538.5881552.916372

180.42715353.30846134.60071119.638981

180.42715353.30846134.60071119.638981

180.42715353.30846134.60071119.638981

97.3630848.3125069.9616296.0108

00.3548760.6403420.064894

00.3548760.6403420.064894

97.3630847.957639.3212875.945906

30.9186110.1664531.1724280.049654

64.6216826.3905748.1002265.757793

0.1132280.1718940.0486330.138459

1.709563000

0000

0000

01.22870900

9.60570834.57867211.2407584.038066

00.05705600

4.3185430.8722470.5194150

4.3185430.8722470.5194150

2.9996931.5231439.8610624.038066
0.294076.3227242.1887961.118142

2.35566822.8725717.0529592.726072

0.3499522.3278480.6193070.193852

1.9570861.4683930.8229620

1.9570860.2041360.2401140

01.2642570.5828480

00.65783300

00.65783300

0.33038900.0373190

0.429198000

0.429198000

0.429198000

4.4939342.99574.0569921.886904

0.078490.2635070.0507630.065784

01.8906782.9925991.306037

4.4154440.8415151.013630.515083

4.4154440.8415151.013630.515083

68.5352297.4215839.3413327.703211

68.5352297.4215839.3413327.703211

68.5352297.4215839.3413327.703211

0.0237563.4893533.8410191.749897

0.0237563.4893533.8410191.749897

0.0237563.4893533.8410191.749897

0.0237563.4893533.8410191.749897

0.0237563.4893533.8410191.749897

0.0237563.4893533.8410191.749897

17.9952791.0437312.9680440.529258

17.9952791.0437312.9680440.529258

17.9952791.0437312.9680440.529258

17.9952791.0437312.9680440.529258

17.9952791.0437312.9680440.529258

17.9952791.0437312.9680440.529258

0.7624164.7296777.8467633.013176

0.7624164.7296777.8467633.013176

0.7624164.7296777.8467633.013176

0.7624164.7296777.8467633.013176

0.7624164.7296777.8467633.013176

0.7624160.23877800

01.2086191.7368630.761168

03.282286.10992.252008

02.15491.3256261.059664

02.15491.3256261.059664

02.15491.3256261.059664

02.15491.3256261.059664

02.15491.3256261.059664

02.15491.3256261.059664

817307.258616125125.99813122389.57792180360.6701279998

06.15763712.9230064.609471

06.15763712.9230064.609471

00.46015900

00.46015900

00.46015900

00.46015900

03.4259678.8559522.396021

03.4259678.8559522.396021

03.4259678.8559522.396021
02.8825135.299542.396021

00.54345400

003.5564120

02.2715114.0670542.21345

02.2715114.0670542.21345

02.2715114.0670542.21345

02.2715114.0670542.21345

05.2716759.5004354.184744

05.2716759.5004354.184744

05.2716759.5004354.184744

05.2716759.5004354.184744

05.2716759.5004354.184744

05.2716759.5004354.184744

000.0721590

000.0721590

000.0721590

000.0721590

000.0721590

000.0721590

05.1737368.2942874.105673
116.87176645.52084660.74026831.314993

116.87176640.3471152.44598127.20932

39.2885677.258849.1323595.072822

00.258090.7599020.164173

00.258090.7599020.164173

00.258090.7599020.164173

39.2885677.000758.3724574.908649

39.2885674.3488853.5498853.282331

39.2885674.3488853.5498853.282331

02.6518654.8225721.626318

0.28509123.96147634.08847116.667696

0.28509123.96147634.08847116.667696

0000

0000

0.28509123.96147634.08847116.667696

0.28509123.96147634.08847116.667696

77.2981089.1267949.2251515.468802

77.2981089.1267949.2251515.468802

77.2981089.1267949.2251515.468802

77.2981089.1267949.2251515.468802

0.7781210.1141120.4453040

0.7781210.1141120.4453040

0.7781210.1141120.4453040

0.7781210.1141120.4453040

0.7781210.1141120.4453040

0.7781210.1141120.4453040

1.236948000

00.2552320.062340.249025

00.2552320.062340.249025

00.2552320.062340.249025

00.2552320.062340.249025

00.2552320.062340.249025

00.2552320.062340.249025

653.6491675547.0126625850.9887286224.833915
0.25719614.96533119.4773446.215805

560.588025222.7997045612.951586022.197405
1.61249193.781373170.31099833.575124

0.12673537.41828573.90963713.923338
508.554963781.6023584574.2586133683.368312

04.3059170.2852760.162796

04.3059170.2852760.162796
04.3059170.2852760.162796

0000

66.85619123.05631728.18753413.180074

0.535359000
66.85619123.05631728.18753413.180074

0000

01.9242595.0144661.728903

02.110444.3451730.845727

04.8382496.8309022.100326

66.3208329.8302327.0121816.30644

03.2879083.618741.712607

01.0652291.3660720.486071

0.3117715.7226958.0535141.176607

05.7226956.5941961.176607

03.1542556.5941961.176607

02.5684400

0.31177101.4593180

203.185782150.838738209.35147598.54046
1.2397537.64685417.8375924.971444

27.0492470.640712.8859241.530747

27.0492470.640712.8859241.530747

0.41926914.25812353.7586332.814709
21.3091126.283997162.45133875.243141

4.48508387.98647351.0761.585219

06.92945921.6600012.048247

000.5930280

000.1076350.260465

00.2880870.5371110

00.4133892.6259690.143115

00.0638080.7144050

1.379495000

15.02525310.28251923.8277835.473261

00.1815740.0981950

01.0172851.025250.330079

01.06621200

03.7970686.4333282.588046

152.63686214.16887815.45585513.721034

0001.037342

152.63686214.16887815.45585512.683692

000.3447970

000.3447970

0.950820.0570564.5935450.863161

0.950820.0570564.5935450.863161

0000

0000

02.0412435.7824242.210933

0000

0000

01.822880.2223860
238.0744813560.2604064254.4711773556.385037

0.08264000

0.08264000

234.9945283557.2550554253.3975653555.797079
12.521457436.623805752.955325152.407646

03.7537432.8517350.695445

00.3184160.0249360

000.2763420

0000

0000

01.4302022.1060310.839659

00.3666280.0652130

191.76019967.72094389.6735331.582296

0.082884102.949205234.65677145.788543

0.4192693.1087495.4428120.537272

1.199680.053156109.04347728.061363

000.02780

04.7070539.4588151.302909

000.463430.076976

00.730951.6036930.662413

00.3872730.1042090

001.1248620

00.6914860.4596780.305394

00.628220.4125120

03.59521520.5520581.806942

003.3245840.800015

00.7083891.1316240.103135

00.1104720.8837650.198518

04.2042217.9254750.485978

00.0975910.1444340

00.0676570.2973540.075239

03.7557631.9741310.876826

00.006960.0825220

00.2210430.1705340.092909

29.0111192840.9812523005.9316673289.097601

00.0366630.2282460

2.9973131.1824710.8512260.587958

2.9973131.1824710.8512260.587958

50.4205691347.415973868.3819692305.253969
0.0451761.8835720.7667051.525608

1.55648717.15245614.4706379.165619
7.952833206.724012230.657033769.093321

0000

0000

0000

0000

0000

000.0741290

0000

000.0741290

0000

0000

0000

00.5240541.6982040
00.5240541.6982040

0000

0000

0000

0000

0000
0000

0000

0000

4.128372189.032552213.957593756.740694
0000

0000

4.128372189.032552213.957593756.740694

0000

0000

0000

0000

0000

0000

0000

0000

1.50311700.1186050.5076
1.50311700.1186050.489878

0000.017722

0000

0000

0000

0000

0000

0000

0000

0000

0000

0000

0000

0000

0000

0000

0000

0000

0000

0000
0000

0000

0000

0000

0000

0000

0000

0000

0000

0000

0000

0002.609941
0000.874462

0000.324636

0001.410843

000.163060
0.0743780.014950.3378650.069467

0.0743780.014950.1748050.069467

0000

0000

0000

0000

0000

0000

0000
0000

0000

0000

0000

0000

0000

0000

0000

0000

0000

0000

0000

0000

0000

0.690479000

0.690479000

29.1475741125.185512619.4031411527.968905
00.8856350.1910770.121455

0.12804720.1565668.2029290.906955
26.918129112.27694149.70683410.054067

25.49488615.61831817.6552664.849711

1.09817457.22566411.1366711.361862

0000

03.0335650.1611270

01.4053740.4911420.039972

00.037520.1004610

0.0282943.3041161.7444280.13915

000.1390840.847985

00.0403500

0.0587447.0702529.0659191.781386

0.1099842.2605890.9235470.127046

00.22601800

01.5966810.086260

00.30192800

00.3637450.2938090
2.2294451012.022936569.505231517.793383

0000

2.2294451011.659191569.2114211517.793383

0000

1.6791050.7964040.7230120.376196

1.6791050.4736010.4679830.376196

1.6791050.4736010.4679830.376196

00.3228030.2550290

00.3228030.2550290

0000

0000

0000

11.59588112.82647316.8320786.289939

0000
11.59588112.82647316.8320786.289939

00.5146650.4230770.306247

11.5738282.8205162.5715421.144846

01.401742.4623460.411254

00.18637600

0.0220537.90317611.3751134.427592

06.1464999.9479863.970737

06.1464999.9479863.970737

06.1464999.9479863.970737

06.1464999.9479863.970737

06.1464999.9479863.970737

43.337464294.250028200.558301186.21012

02.8515725.1627572.110502

02.8515725.1627572.110502

02.8515725.1627572.110502

02.8515725.1627572.110502

0000

0000

0000

0000

01.555062.1499480.846881
43.337464291.398456195.395544184.099618

0000

0000

0000

19.4234414.1999245.9473573.978434

03.8299265.2234112.404455

03.8299265.2234112.404455

1.0449760.3699980.2989790.219984

1.0449760.3699980.2989790.219984

18.37846500.4249671.353995

18.37846500.4249671.353995

9.1443981.9781022.1778891.09453

9.1443981.9781022.1778891.09453

9.1443981.9781022.1778891.09453

010.38949616.0634217.723765
02.7264324.0573981.296314

04.4660996.8850024.499995

04.4660996.8850024.499995

03.1969655.1210211.927456

03.1969655.1210211.927456

12.968462269.82697164.267756167.822779

05.8573629.778014.921831
0.76244924.94808435.64244918.455875

02.7549965.144823.261649

04.1890685.2553243.672672

000.0107790

0.2434470.2211870.2198140.015639

0.5190020.95511100.657786

0000

00.6428481.705740.086386

05.5286027.4031783.19255

04.798916.1247842.647362

12.206013244.878886128.625307149.366904

12.206013244.878886128.625307149.366904

03.2546734.5250942.164346

03.2546734.5250942.164346

03.2546734.5250942.164346

1.8011630.1942310.2640790.0431

0.1813330.104990.1055770.0431

0.1813330.104990.1055770.0431

1.619830.0892410.1585020

1.619830.0892410.1585020

0000.425783

0000.425783

0000.425783

0.0977240.8563371.9962090.47214

0.097724000

0.097724000

0.097724000

0.097724000

00.8563371.9962090.47214

00.8563371.9962090.47214

00.8563371.9962090.47214

00.8563371.9962090.47214

49.3687637.9947636.0573085.767708

49.3687637.9947636.0573085.767708

49.3687637.9947636.0573085.767708

49.3687637.9947636.0573085.767708

49.3687637.9947636.0573085.767708

19.4781184.535573.7123362.350926

19.4781184.535573.7123362.350926

19.4781184.535573.7123362.350926

19.4781184.535573.7123362.350926

19.4781184.535573.7123362.350926

19.4781184.535573.7123362.350926

01.916432.7649740.79455

0.1220390.9584932.3613750.796112

0.1220390.9584932.3613750.796112

0.1220390.9584932.3613750.796112

0.1220390.9584932.3613750.796112

0.1220390.9584932.3613750.796112

0.1220390.9584932.3613750.796112

0.2980.4534460.1962650

0.2980.4534460.1962650

0.2980.4534460.1962650

0.2980.4534460.1962650

0.2980.4534460.1962650

00.0606240.1175230

0.2980.3928220.0787420

1.9297460.4558790.1978610.337235

1.9297460.4558790.1978610.337235

1.9297460.4558790.1978610.337235

1.9297460.4558790.1978610.337235

1.9297460.4558790.1978610.337235

1.9297460.4558790.1978610.337235

0.188115000

0.188115000

0.188115000

0.188115000

0.188115000

0.188115000

15939.9287891642.9220942336.340695846.09765
815435.594121118686.172718115741.78361673225.7612379999

240.87734344.7427154.79604137.01052

240.78570644.68283454.34326937.01052

03.4903426.4819793.046324

03.4903426.4819793.046324

03.4903426.4819793.046324

03.42978911.3066246.130218

03.42978911.3066246.130218

45.8664417.20043610.6958825.679317

45.8664417.20043610.6958825.679317

00.8726215.5682641.432489

45.8664416.3278155.1276184.246828

191.93666229.77174625.21112721.431035

191.93666229.77174625.21112721.431035

191.93666229.77174625.21112721.431035

2.9826030.7905210.6476570.723626

2.9826030.7905210.6476570.723626

2.9826030.7905210.6476570.723626

0.0916370.0598760.4527720

0.0916370.0598760.4527720

0.0916370.0598760.4527720

0.0916370.0598760.4527720

150.00518816.71480514.51830713.036431

149.83870116.71480514.51830713.036431

03.4463074.2900362.535

03.4463074.2900362.535

03.4463074.2900362.535

149.83870113.26849810.22827110.501431

00.13584100.10853

0000

00.13584100.10853

149.83870113.13265710.22827110.392901

0000

149.83870113.13265710.22827110.392901

0.166487000

0.166487000

0.166487000

0.166487000

29978.51085600014106.587724999994386.367073000012631.149948
793086.602619113149.961754112534.50036871717.7908489998

60195.30361400054151.7257286373.732288999983942.30079
405796.59931641408.581609999941549.910374999926606.815473

61.4073280.0703310.0224190.054646
377.86357129.99859121.25612318.827405

174.27324215.6741865.1204898.448684
34.9642462.6202680.2052182.120099

1.1733760.18157400.261413

0.4135190.5688500.800015

34.488241.8399270.6923650.399693

74.791074000

0.737140.09413700.246161

26.3554379.204083.4997980.354737

1.035022000

0.3151881.165350.7231084.266566

23.9431752.1725844.4392832.515683
18.7144551.0721751.9261221.603683

4.6407680.5463670.715230.412555

0.5879520.5540421.7979310.499445

0000

1.1682780.419490.2905470.791567

0000

1.1682780.419490.2905470.175841

0000.615726

22.4743081.1264470.5199231.087462
117.07154811.66211.3833857.016825

001.011810.750526

0.5822050.0178720.1914770.393812

3.53129900.0153390

2.2443320.3748650.2536670.110336

42.2376436.3601252.5655970.912451

14.9783960.355981.5506570.470031

3.8566880.5612060.4497950.739654

1.517645000

6.419317000

11.8978522.8655051.1041560.069546

7.33186303.7209642.483007

98.67920.84806210.7602495.400831
9.504439000

0.585241000.019513

0.585241000.019513

0.6413070.1962440.4421240.048675
26.7101299.7494547.1696853.281036

13.3537096.832112.5752041.693045

12.7151132.72114.1523571.539316

0.9622580.2933030.0760310.092353
15.3836382.3069510.0919630.400255

0000

12.2813682.0136480.0159320.307902

2.140012000

46.4955538.7916573.4986011.700027

46.4955538.7916573.4986011.700027

61333.76887199985798.475658000014809.955404000014443.12184000001
581.696443112.235244108.42180185.777718

210.05870832.41835624.05676522.328599
0.4230351.1512390.9190251.223326

1.8271380.752231.107930.552789

0.941875000

10.0910090.1795150.0897940.189451

9.7659600.8717350

4.7925394.0255171.4060532.183097

1.30298300.040620

1.0840651.7282440.76022.516906

3.9330811.9806791.3356821.000165

28.6431571.5838262.6560451.433527

13.5991260.3014910.1656450.087949

3.9861790.3719950.8001870.378755

0000

1.1511692.4328911.4596611.640764

0.5499320.1356290.0422220.008284

1.8163340.8099360.3965022.374383

0000

5.197582000

117.14153816.35919711.703198.229131

3.8120060.6059670.3022740.510072

4.7770822.9305520.9807871.639216

4.22225200.3396410.371954

0.554832.9305520.6411461.267262

27.834324.6530612.1012672.077594

3.780604000

24.0537164.6530612.1012672.077594

54972.99931499984826.580306000013970.830992000013699.04042300001
60445.71698299985636.239451000014658.929720000014320.40517700001

26.5656316.7649113.7091643.355717

419.52707483.94356762.50268250.088914

14.5191551.03830.9514451.243511

48.4910722.98933513.09041520.343349

14.70151700.4125120

0.3673890.1498690.0714950.015254

23.9400479.5934086.1256885.861426

32.7778342.7735733.8769091.944613

24.0903811.0752620.2322230.650157

5.078167000

0.6514243.2159911.1536362.705686

43.7082531.3351030.4991820.495756

25.7655352.0824211.6485330.915074

3.3772312.3110220.8923911.318833

613.7275842.6162642.2157871.165969

20.9997162.6215773.0435711.957661

1307.964834422.715071327.231202314.347441

1.9325930.1162680.0482140.074212

497.7414429.06767827.81635325.003553

15.1622341.926332.1248350.902663

46.7285471.7588894.4834151.067841

19.75203500.5806920

0000

13.4517892.3106562.6541552.056543

4.0974721.0590580.988952.43118

0.1688520.1937180.1594830.40502

103.1045551.5910253.8348690.666287

43.7650120.7772971.338161.581009

62.07755210.3656715.1915646.435593

6.5205881.17222100.369853

8.2770382.9963452.2535421.864957

0.62388400.1954960.506029

0.355245000

370.4558723.2266922.16098220.769172

37.94436.0987575.4504356.275961

3.8538870.61330900.839939

5.8697950.3248292.5508260.394683

9.9024322.0481541.6351621.573212

2.7307251.7688090.7012710.873309

87.0903226.6699214.0266133.338238

8.6172440.9335191.7776071.006608

7.5172123.0368932.1287521.889007

2.9726560.7210480.5069630.27494

27.1704252.043741.6785661.674632

4.7789871.2566032.155821.425768

5.951251.8326321.198233.006219

510.0391041.383292.4744341.783553

22.989641.0926241.0078120.978758

40.0177081.7374895.1444382.207124

9.7451860.936150.9634241.058268

6.8316480.2633470.2383020.282748

148.39533428.18015518.71688116.98334

1.7402840.2032230.3354570.100947

421.71573900000190.695649119.09397789.5641999999999

13.0256664.6913424.8149813.120642

28.7550543.9595022.5954393.511952

0.6588461.5771271.1736320.108858

16.8109142.4476142.7098733.137784

0.5940830.098760.1269770.21934

202.6360021.2515331.9101311.953491

3.4702860.536220.4037270.923117

0.52509700.045580.464561

18.6397970.0678960.2980390.255838

1.2604971.401490.7478341.594444

58.4631318.44239614.44349110.028339

58.4631318.44239614.44349110.028339

5.2222051.5565981.0215730.865197
0000

3.3305231.4157240.8148460.865197

1.8916820.11829200

00.0225820.2067270

1274.237377211.207234194.484414109.47639
6.6496692.4981120.5324320.102617

1.00144400.0633091.334
179.80945812.06907813.06187110.416715

0000

29.10064901.2012190

0.29635600.0209780.018649

123.7546946.3124096.5270345.327371

0000

0.946477000

0.7355970.8719310.2684380.207353

23.9742414.8847384.9808933.529342

15.6163065.6697782.3516220.320148
0000

15.1733155.5365772.3106290.14614

00.10782600.137085

0.4429910.0253750.0409930.036923

9.9570215.3137312.7661432.720123
13.7622435.8078683.5905963.131377

000.0473260

3.8052220.2456760.3813970

00.2484610.395730.411254

1.440879000

1.440879000

19.36823200.0403630.052379

19.36823200.0403630.052379

0000
1037.59059185.162398174.9075395.453154

1036.546338183.827212174.13248894.001713

0000

0.8175270.3005820.3307470.150846

0.2267251.0346040.4442951.300595

11.05833300.0617640.007432
1.209778000

2.37273200.0125070.007432

2.37273200.0125070.007432

3.171659000

3.171659000

4.30416400.0492570

1.326504000

2.9776600.0492570

000.1970270.064894

000.1970270.064894

000.1970270.064894

281022.72796631016.921974999930021.637993999918007.649563
67728.51960599978722.052817999968759.022027999955454.29861200001

540.22707268.4073657.5966136.433842
18.4892256.8207648.3774667.331885

233.2396820.82268315.2838589.685063

273.79949739.59666632.52608718.544308

11.9988270.9992261.0833890.550732

2.6998430.1680210.325810.052802

0000.269052

0000
5.6366270.2444980.6667650.105988

0.70264000

4.9339870.2444980.6667650.105988

68.6403988.8487311.8576868.399661

7.8555763.1541947.6522964.815674

3.591940.1172490.4990430.727096

57.1928825.5772873.7063472.856891

0000

2.7213880.1513070.8871930.200413

0000

2.602094000

0.1192940.1513070.8871930.200413

2.3720230.2604260.395950

2.3720230.2604260.395950

9.0371470.0787220.977680

9.0371470.0787220.977680

958.38425699999924.22985442.336348.872306

958.38425699999924.22985442.336348.872306

27.015926000.929504
108.8943397.0209444.6340954.099758

47.7107513.9957482.7172.141536

34.1676623.0251961.9170951.028718

0000

88.4204656.4281194.9669482.252548
167.1314115.718314.6188489.149974

1.5800130.4952510.1150350.380263

0.9084170.0160270.0262260

0000

0.6540910.1934280.2156430

0000.337423

26.8129150.8160730.5867450.706227

34.6886314.613252.5944472.266021

0000

0000

8.9229753.1561525.31833.207492

5.14390300.7955040

75.0637237.4301419.8098548.654133

75.0637237.4301419.8098548.654133

72.96444619.4102655.3249391.762275

72.96444619.4102655.3249391.762275

15.3176922.5222011.0538040.610871

15.3176922.5222011.0538040.610871

234.90450526.24297724.60591726.543984
49.3219464.2191445.6078445.633205

63.4471288.8805678.81286610.982848

8.9185190.0298340.1494540.074784

50.9016410.5486587.6619316.33083

57.049842.5647741.7718462.984293

0.5498200.0447350.070656

0.024877000.007696

4.69073500.5572410.459672

4282.261957551.882912513.177011318.01728
47.8855198.2931563.7467351.284552

0.0287320.562480.6272170.669291

3943.518354519.821697478.902185305.05166

213.7307728.64909915.9336054.485101

4.87327000

72.2253114.5564813.9672696.526676

10.9089480.0440390.8602610.924721

10.90894800.3759080.819964

00.0440390.4843530.104757

837.633723120.115289130.805861101.084551
351.81744542.45350658.48421743.724123

101.1417655.0735715.2506861.793026

3.2831320.0527010.1995610.131568

82.88594112.8145929.7946457.058216

0.1694040.0172940.0284410.017259

9.0371342.9309133.5895842.874147

207.95356238.87611545.99483839.229859

17.5935092.2882332.8248692.307315

63.75183115.6083644.639023.949038

7.08441200.98171.295446

7.08441200.98171.295446

79778.53918600015480.7182982735.4936122939.863879
63125.2628723807.492331818.2326342144.274454

40.6735061.0666431.2847221.017311

4.4731390.0184910.895870.178962

2789.395732273.204003201.893575130.136971

37.8266330.7034951.1444950.290838

106.1743798.6029129.2946954.624685

27.2267083.0816651.192660.556601

1.08818700.0118550

81.5892393.1333670.7503641.288393

3.7614970.0828820.7724850

9.1456142.3784310.2939950.311173

0000

5.5483010.29305500.242861

2.556559000

0000.162843

0.25032600.0792210

3084.740655407.620891230.888025167.434447

181.5335785.9647564.874995.257799

19.42322300.0363680.121051

8.8793410.4333790.3491530.625753

61.1676173.142643.7822812.448609

00.0646580.0779020.06317

28.4748020.2914570.3168460.413909

3.18497300.0862750

37.48164412.1571884.8214490.430936

6.6360060.2932230.4045520.704294

72.479165.6678755.0511212.358135

0.322366000

2.9867070.1631340.5442270.413126

472.57665123.29171524.99179126.485051

5.74389200.2417420

50.5437661.8265861.5980470.687939

12.0428340.649180.4455470.452985

2.8633141.0114060.2399080

62.7108121.8668781.2416182.932226

0.400115000

0.4872070.0056990.1210450.222805

33.6008963.0090171.5151971.135771

499.39248387.21739949.57935926.240954

15.672790.1519710.1099550.144505

0.026139000

4458.652318570.770204236.042336272.594092

4.0852320.0605960.3471560.690005

0000

7.9603680.279250.4271890.040167

24.7187345.0362950.743890.353114

11.3615050.2081010.3346920.233568

68.44461411.103032.9188533.682382

758.81047466.02799133.32694338.607993

2.642195000

5.7136870.0617141.1511371.461071

0.206858000

0.80359500.0924380

26.2436592.3084780.6392940.740067

0000

90.7746060.2596650.6343860.967369

18.4714992.11687200.368352

2.08838400.1158580

1.136654000

106.1841524.6891943.0581714.108801

2039.696637101.56818653.96801360.01634

570.1681715.0741735.8737975.626861

1.5451460.0465940.086260

1.738020.6885960.5621160.28662

143.7111444.1921292.7169421.943848

452.49895337.91263916.0240918.062248

35.2446896.831025.7531473.651865

2.3881270.0567820.6023251.203638

00.1123730.2205810.282408

8.2736090.0562040.0723750.150349

25.8774244.5427511.8297712.054448

10.7550691.8291350.7878831.079716

34.0182770.5542932.4983450.334761

33.7841270.5542932.4983450.334761

0.23415000

110.48866321.14802614.36355311.014138

110.48866321.14802614.36355311.014138

45722.0786228782.7883284184.3898452302.639491
37172.1213637182.811650999993401.72631866.609485

5.5595022.0566740.5805620.536375

8331.069935999991548.669562761.912376428.48087

50.4678677.7468683.7093141.723084

17.3685423.4909012.5540882.648928

145.49141238.01267213.9072052.640749

1269.063868182.449896143.56910789.99967
58.0865315.1218424.2128152.875134

1198.72882174.893001139.29998586.305596

12.2485172.4350530.0563070.81894

2033.944173197.258317177.25487882.187641
862.49453200000147.9310147.95031615.247192

4.85630.09791800

6.8619521.4489660.4883960

1.198370.1137350.1011060.031603

7.2741621.0505163.1109930.750902

0.9381320.3921280.4437870.175899

0.50434400.0532920

67.96192124.0688632.4750750.881139

23.5689674.5734862.2118650.029552

23.5563483.4243012.5421961.075653

453.46045738.62213226.956219.955601

1.1314990.20412400.379017

0000

0.7053670.0353180.0252760

6.22537601.5224372.129639

4.491970.8023871.0950250.454814

193.12406821.42224935.39539611.150635

0000

2.3552100.1070720

15.7928922.6991631.0589180.154604

4.91285900.5396741.805457

4.36883600.5062670.197749

0.490878000

31.8574174.1855163.2669210.687052

0000

49.1566773.7757673.0687440.152989

2.322035000

12.5109233.6794324.835041.291406

1.9509301.0189230.371954

249.87175138.73130638.48195925.044491

0000.220293

30.0589833.2669572.9927472.458474
7.2090820.0172940.1130480

15.8779892.4463231.7250522.138864

6.9719120.803341.1546470.31961

2.9740190.3908192.793290.691329
0.72823800.1954960.050603

0.597549000.058427

0.48739700.5768870.273562

1.1608350.3908192.0209070.308737

466.66573596.89180962.4709362.703385

0.1355121.8140870.9162540.750483

120.30845141.21725333.33189530.357086

4.8471020.4834630.8260390.213448

90.45328613.2216953.1838012.056819

11.4276836.9366013.9385352.280457

110.4083944.2005551.7607811.840924

129.08530729.01815518.51362525.204168

23552.168268184.416819437.200035185.724435
21461.835045135.775262368.846793156.485678

5.3261880.2195830.8893970.477339

76.70001415.4049459.9048371.194369

127.0481534.0197957.8768115.107942

1867.84195828.99723449.23231522.34357

13.4169100.4498820.115537

113.61542922.1122798.1448662.671444
53.58735814.9343434.9787131.437097

0000

1.2592320.3068840.2060110.230321

0.25220400.0328950.041954

4.5123160.1005560.1850750.012308

9.309819000

7.0536942.6478980.8413410.305217

14.8780763.2612551.0324420

22.762730.8613430.8683890.644547

47.9651353.858589.9102257.075784

37.8328463.858588.2583526.956255

10.13228901.6518730.119529

5767.9525171387.5492561939.530944523.304913000001
152.44548227.91766639.24102412.386946

82.75710418.22504817.362496.582541

5532.7499311341.4065421882.92743504.335426000001

32636.3159532562.421352000016052.1936033148.031133
46965.4914185078.85751510721.4130755816.526404

290.58984677.78209260.30846723.718832

363.82308187.997484199.212242100.180465

40.0682772.2754430.2974640

26.4844880.0396850.1297560.281182

61.8938810.45058910.6569658.158686

27.4527333.248510.4666463.436385

10.4965450.3719950.160220

0.65255700.1493120

1.38513400.0632890

3.03529000

7.3184030.0249030.0344150

511.56697915.58094323.9807168.994117

0.5837100.1046740

44.08026627.59450450.49365228.560472

1.5931080.0209920.2405450.021444

78.5692830.597211.9100010.570283

00.2019881.0372770.187012

130.4796460.7371961.8988030.696196

7815.027574999992018.0090843822.3488812229.470602

3671.171709133.521042177.394757108.881501

624.79684917.49606120.31981810.080652

0.4718640.05555500.112548

617.634242120.430897288.011572145.144894

1480.841123178.697021115.80669878.646086
30.464730.8085680.7056911.384979

102.4360166.4282556.0050984.777146

1.454109000

99.8062596.4282556.0050984.777146

1.175648000

0.167493000

0.167493000

83.57219316.7730355.9907071.186089
255.20489946.11990426.40579817.841732

000.0480230

1.2535840.1425310.0881540

7.3259531.6583492.5644974.311295

104.83787419.94371711.7390947.008224

4.643560.3399370.2865760.131337

1.1639970.3276040.0819350.705094

9.3865142.0949141.5108470.87278

6.8608564.0187882.5382981.073771

6.0764070.574191.1594331.283338

4.2588240.1551090.2831460.859839

6.5711640.0389430.020620.325191

18.8586060.0527870.0704990.084774

0.39536700.0239690

2.891081000
13.7646060.3785750.8614220.277139

1.395778000

0000

0.258540.05448700.013289

0000

0000

0.057518000

000.099190

00.0390410.4893610

6.7588530.0169370.0629440.023443

2.4028360.268110.2018640.230779

0000

000.0080630.009628

0000

0.9935360.1035820.3083080

0.9935360.1035820.3083080

0000

0.078942000.032797

0.078942000.032797

5.9730010.6421100.034939

5.9730010.6421100.034939

366.2650597.7869878.1857755.147874
1071.7579124.21602781.52038154.297354

560.50453490.63097555.4164333.002471

2.6483630.86750.9346370.411711

0000

17.8847984.3628731.4755581.854179

00.09791800

0.437338000

000.0226510

5.9719610.8752570.751031.907034

53.7930067.3026016.1507175.799811

1.095673000

5.340093.4858110.9672410.125579

0.798188000

2.2424390.10315200

0000

1.41543800.3858310

0000

5.54702300.010640.031534

29.4249285.2222943.4322092.866236

8.4316462.0604251.5981680.82874

6.7162020.6282200.430598

1.2475330.0457650.0814740.16979

1.3012610.2001580.6065960.295844

000.8553530.485722

0.692420.5270140.5986820.920978

00.0190770.0473890.019253

2.119460.7073412.0184131.320242

0.7384810.4578380.360210.739676

0.7384810.4578380.360210.739676

1.3809790.2495031.6582030.580566

1.3809790.2495031.6582030.580566

5.0895480.7643572.2828420

5.0895480.7643572.2828420

5.0895480.7643572.2828420

272.3307552.87810740.84962726.599148

216.72103536.0884929.60781420.595292

176.27528626.33643220.54463416.575162

176.27528626.33643220.54463416.575162

40.4457499.7520589.063184.02013
001.144190.439769

00.9955711.6799550.17313

00.16380200

0.7024510.19632800

00.09892100

39.7432988.2974366.2390353.407231

0000

0000

0000

0000

0000

0000

28.36386711.4901427.0112283.542097

0000

0000

00.32615800
28.23927110.1913736.5806663.472785

01.7261521.8160370.064027

0.0615531.5304141.1650790.034245

1.161096000

26.9427113.4091753.1428783.374513

0.0739111.3841340.4566720

01.8153400

0.1245961.2987690.4305620.069312

0.1245961.2987690.4305620.069312

0.45061.1781111.1654810.393113

0.45061.1781111.1654810.393113

0.45061.1781111.1654810.393113

26.7952484.1213643.0651042.068646

26.7952484.1213643.0651042.068646

0.793569000

26.0016794.1213643.0651042.068646

57.1796168.2781626.7837175.348607

57.0119547.9259186.5510125.330853

56.6025767.9259186.5510125.330853

56.6025767.9259186.5510125.330853

0.409378000

0.409378000

0000

0000

0.1676620.3522440.2327050.017754

0.1676620.3522440.2327050.017754

0.1676620.3522440.2327050.017754

36631.258197000214860.02418912864.2800348608.83136800001

35.8359015.5365025.7827653.071859

36595.422296000214854.48768712858.4972698605.75950900001

0.86218601.6522040.371954

0000.371954

0000

0000

000.6605460

000.9916580

0.862186000

1.4490460.0223570.4618730
0.59992200.3868580

0000

0.44710.0223570.0750150

0.106873000

0000

0.295151000

0.421829000

0.421829000

36592.689235000214854.4653312856.3831928605.38755500001
24194.012632000212028.70210310125.0488947024.955054

0000

4.5751530.53890900

44.9011091.2624231.8360250

1.450742000

36.5041213.0230121.5584290.560814

0.0417000

7.3329315.4649815.3898964.16742

14.21797912.33544510.9428535.378948

3.5026740.5862860.2157610

02.3124871.5218680.399652

0.270628000

0.2890660.3603630.0259850.065102

3.7171810.8295040.4163610.20064

1.3015853.536982.290643.612178

3.6445940.0190770.0492140.1606

7.3352450.5091271.0833860

2.5760781.2902691.3424440.236926

00.29518800.16738

3.43223700.4658740

148.726342.2767672.5411540.491651

4.56722.0346333.6844970.561406

3.0319570.2332810.2027340

5.1163445.893920.9546050.242344

1.232960.0639440.0826720

41.819012.5217092.5881150.173273

93.599932.6814885.8895681.231812

13.8016591.624081.5847850.542038

0000

00.06536100.133387

7.18339500.1101150

3.693062000

0000

9.7244060.3860220.819010.216532

1.5718470.07565400

1.9794222.6606571.912180.03862

0.3983560.1457650.1032460.01719

2.2725730.8455941.3459780.143513

4.86268400.0594710

1.50511112.1771899.7067157.768033

6.61337500.0735850

5.44121200.4608660

14.6853941.8895281.6278170.130976

0.4353431.8545280.7145960.413452

437.83754368.46912967.66219949.6903

240.962356.3386377.7548694.118079

4.2759120.3980430.6370810.235415

0.3734260.3033441.4490840.141896

1.669959000

2.844760.1877360.02780.050568

0.114958000

0000

0.62324500.0493160

268.268028409.391906439.340791336.892232

0.0331790.05223800

54.3053166.60624712.2545021.169262

0000

00.21377500

6.8525460.1635870.2142150.051182

3.8445624.5547142.5049263.470629

1.2302123.6052181.8198411.910871

2.7681740.0234610.055810.022106

0000

451.281077565.780472381.95293331.563455

0.446597000

1.067681.1506651.1055570.430046

9.2123410.0663440.0055510.006585

4.8493160.1478850.2300840

1.22197400.5384630.123706

0.1067620.2492770.1798920.110213

0000

62.9663633.289911.4188541.26407

0.0217060.0476040.0222440.031392

2.8870411.4009361.0503060.110146

7.4949952.5309481.7221190.81536

6.3820840.5332941.1395450.634834

3.4395421.1809782.070860

11.6453050.0757090.291130.038166

6.0443462.2625271.782810.924949

0.101011000

0.8900040.2032452.5152740.086386

0.09630.1067370.3150130.040037

2032.240629713.045934659.001466405.597179

0.045983000

5.5647160.1201820.3829010.142357

7.127632.3826333.8148970.853394

44.8052683.9301173.4726342.456832

28.62326924.17672117.7224838.703705

0.7235552.313541.1498040.532735

03.0532900

153.227975277.108373290.31505754.5736300000001

7.3840241.0581171.2935651.430982

1.15955600.0440870

0.7492090.2876850.9360470.375062

20.2966134.5289215.0280670.741641

0.066549000

1.8252050.0287410.7477780.008083

0.0513840.8730071.4497240.978604

0.0958280.06217600.241562

0.4683080.5929040.7061140.094011

10.045229000.329708

00.1069080.180730.06317

11.998850.0604820.1105710.008753

3.9402550.8197521.2986610.428053

5.3234640.5616821.1319861.146315

10.3873871.3376250.8710270

0000

0.9496562.6941731.2767021.609458

00.29739700

5.6145930.8970161.5108330.181054

00.5493430.1295970.963599

71.55351999.2866769.79007452.63523

1.305240.3962350.5275060.061854

0.1008820.6959550.3786390

0.762416000

1.7705870.2691770.5158190.107261

5.1277338.4336187.0771446.723116

0.42381900.0489860.044357

10.0309742.7962542.33781.427787

1.7800994.8674043.629025.696158

00.4128230.5090570

8.2269773.4976655.0047852.126851

3.6591020.0247290.0328510

2.7765210.06158600

002.1371080.67012

4.35707200.1257720

0.383128000

0.7634680.8771110.1777810

100.790565268.321753287.242332155.671108

1.08810200.078390.022917

1.2974832.6566671.375471.393346

0.92934000

6931.61273700001100.500106202.28104437.29416

3.0523430.5572910.8599160.208559

1.196115000

134.1554764.8921467.406190.944458

0.9416853.4370283.0247451.976517

0.9670450.4775550.7637920

28.6452911.4671871.6346850.252144

0.0226880.0751180.0465940.037833

1.1910630.01385700

2.3403860.12877800

000.2344090.128317

2.3917080.30254400

3.4316070.1691130.0753630.696749

6.14324500.7384220.019425

4.931514000

0.0633040.3233740.1356170.052972

2.3843062.7914061.1700481.440553

0.195023.5096462.8871350.107445

23.2123451.927883.0883511.18489

6.3444422.4234671.709391.21399

14.5057980.8606230.3317870.089043

43.2251736.8853426.7423225.41845

4.1159595.2750212.3173853.508016

0000

16.5502681.3250042.5803020.769823

0.3710620.4230620.2254350.032797

5.21061401.0286250

00.82810400

0.028759000

1.2410520.6707681.3764021.704195

01.165441.3109380.591969

31.522510.8368862.1840380.547533

40.7497021.2115633.3460020.77855

2.2913360.0199020.0605520.065122

1.6817251.1301851.1496460.879709

3.1610280.0407390.2008870

0000

25.8367455.2665137.0226925.274174

12.3527560.8358264.2111370.615726

0.289673000

00.1127830.1080420

0000

9.2797630.3841590.5841310

1.844730.0157640.0257710

4.0523750.1112690.4751170.068752

4.8252971.2764540.8652580.335118

3.163331000

5.9231113.9701345.2908722.868859

0.4107960.487330.8225720.182375

10.2237915.9378336.3715283.800373

00.0733930.6564060

53.20304267.582760.464241999999929.728077

143.4612072.0975022.82510.367469

0.08049500.0113120

2.07812500.0614670

00.0649730.0133020

2.8069411.7151311.8181120.503458

0.8259990.0354460.2994590.0156

0.7422620.3762090.2787660.448102

4.4650561.1313571.1776750.743871

117.5519427.52856722.8722936.417077

3.9867090.3255980.4462170.094487

0.0397720.1520480.4480290

766.139472111.016782108.45784876.261396

5.285111000
726.845797106.747166104.50834873.16479

1.54847000

1.54847000

469.1254275.10187972.31469455.085387
0.73642800.0200750

300.71791844.00941734.86768628.93903

79.12270213.66921317.75482310.883571

53.0746337.0465466.3167485.554592

0000

00.7370282.2883810.439769

3.7361791.1093011.1209471.672824

22.9593.5620543.3794492.649599

8.778564.968326.5665854.946002

02.58069500

02.58069500

44.4869368.8157365.9014717.096359

44.4869368.8157365.9014717.096359

122.2970041.14496511.4719370.542834
206.31811520.19752726.27812510.897769

23.2632463.2303512.4997772.065595

11.3586727.6778254.2363444.478683

0.716779000

36.8481218.1443868.0700673.810657

0000

5.747422000

6.086871000

0.0817450.0513290.0140580.085275

0.0817450.0513290.0140580.085275

39.2936754.2696163.94953.096606

39.2936754.2696163.94953.096606

0.3499910.4091350.3575610

38.9436843.8604813.5919393.096606

0.1118840.28696800

0.1118840.28696800

0.1118840.28696800

0.1118840.28696800

73.4952869.6380411.1772754.098684

0.0410190.4679660.1734140.443087
73.4952869.6380411.1772754.098684

000.8891770

000.8891770

00.1078260.5324920

00.1078260.5324920

00.9335591.8077250.083849

00.9335591.8077250.083849

00.531020.4517070.258475

00.531020.1783280.091095

000.2733790.16738

00.7434160.60290

00.74341600

000.60290

73.2729366.5777466.2309353.025474

00.0222540.1306160

000.3324170

73.2729366.5554925.7679023.025474

0000

0000

0.1813310.2765070.4889250.287799

0.1813310.2765070.4889250.287799

11.1288231.8599771.912221.255434

1.4975690.5805460.6663760.303746

0000

0000

1.4975690.5805460.6663760.303746

1.4975690.5805460.6663760.303746

9.6312541.2794311.2458440.951688

9.6312541.2794311.2458440.951688

0.018931000

9.6123231.2794311.2458440.951688

0000

00.39265600

00.39265600

00.39265600

00.39265600

332.3832983950.06334110203.790558741.505573

332.3832983950.06334110203.790558741.505573

01.3202655.9391080.523903

01.3202655.9391080.523903

246.4864983941.84149510187.136851734.935009
47.7633323378.1913448765.64431200002607.875528000001

03.3259999.0423081.102815

47.65073410.52227218.3604466.6158

010.33601622.4340981.627759

1.614410.2135070.4021630.264242

27.54089815.17177719.8915162.188158

022.38038948.3761472.184397

00.873912.2211350.085136

0.9588740.20737600

015.40455649.8497746.955642

0.2738185.18883518.6855211.198119

016.61893558.5920224.254074

10.10604311.828135.0054573.753173

0.3489871.8470264.1928050.273183

01.3072815.6734470.513359

01.4264053.6146690.203005

0.58285714.12656635.9796722.514401

02.8434963.954790.151804

07.13364418.840411.981264

62.77408868.95418146.22764225.110703

02.5130634.8016790.217255

46.872457347.583143904.96621964.859917

00.033360.2926610

00.8062027.2204961.005275

02.9253912.7612770

00.0787220.1061850

85.89686.90158110.7145996.046661

01.0721442.0321350.083308

85.89685.8294378.6824645.963353

01.0481071.0189230

01.0481071.0189230

01.0481071.0189230

01.0481071.0189230

0000

0000

318043.9515646215.031793000241730.024460000132386.3521359999
5.8772480.109240.1807120.040742

40.14565817.27282750.8892698.159257

2.642781.9442320.9204920.525761
40.14565817.27282750.8892698.159257

00.4152070.7444670.033818

09.75331744.1646593.47431

37.5028785.1600715.0596514.125368

90.6011512.06544610.5370848.678659
317953.23340646197.317393000241677.16402232376.0236169999

252242.61008736008.409773000332471.45298225232.2058699999
317582.38870946150.463385000241631.44078532340.6059989999

1.4768330.9802171.8808040.070229

3.8847620.812041.7095030.981739

192.58242726.86706730.02383324.760032

0.345040.1349910.0922950.028678

520.72679781.57449164.87346150.541731

62.38207211.21417311.1495475.761259

000.0642680.085611

342.95397338.45368634.59384130.170897

0000

63.1949457.0613598.8425186.42487

1.6091590.1931380.1632810.087952

2.0714430.5416260.2162190.177361

282.19251727.96995332.27406321.801863

72.2940326.0417286.3672575.908262

54.6230628.49756312.7991496.651886

5.2574870.6209141.1542880.398969

49.0190987.30466.0216195.929228

129.42392314.28796313.43950414.884478

14.77707830.25710833.2476127.376726

2.2453210.5360330.6117220.25023

0.0733910.0284340.2781590.030492

49.47996910.4726289.0308225.766073

000.2181380

109.33792221.56117718.18342218.610898

0.20335200.238480

0.7370912.2606532.6576550.797431

0.0095710.118750.202280.008023

0.0354650.2951490.242830

5.1623942.0840972.4393651.117002

1.3935520.3111730.8891770

385.57500751.59425444.01934335.13938

2069.53291368.142896307.825304233.765078

26.1882893.6685845.6499494.70561

0.1576340.1098760.3572460.087856

4.4810990.8152420.8314880.571427

147.5121428.47013919.5615117.139622

2.4888540.74762.2771090.343936

1475.037421202.88117181.220733150.874845

7.8063191.5309861.1941191.200154

59.84527311.12725911.3096437.890589

89.92247214.35771111.6784029.951146

38.4675955.3940783.8589013.273565

142.93512921.7580821.08695420.689565

64.3382524.439516.4594357.069123

6.8389280.6765890.5425670.62984

1.7583720.55243400.335095

1238.768945259.137004299.950360999999150.129725

1.2855763.9845822.0843631.309106

23.8768556.6880974.98724.549949

365.31362157.67301850.74304742.675123

4.3119880.9781321.2589620.696412

6.2177591.5779731.9170281.122263

28677.2924964336.183463840.9112283107.958619

1.41450403.0524410.371954

38.5215295.2933025.6694925.352456

0.958282000

41.4318426.1937245.6696025.848564

1.807620.3433660.3120830.390437

0000.426447

2.1740260.7980810.602440.237933

14.97513120.52113525.8077776.03597

22.4012233.90093.6611773.341447

0.1908630.2119080.5005280

72.6275689.3809677.7930695.650128

4.1148542.4129921.5745040.666014

108.01460211.387338.89477610.241273

11.9213753.7104975.0365381.834402

0.8445770.70317300

0.0754330.1020620.2219230

158.68691923.9374919.05038615.397912

26.0817835.7741975.6940265.463713

20.3397242.6582614.1881433.743228

46.32074422.27869515.072386.941147

31.6377815.4403214.8606383.890062

87.1736419.16361111.07750410.364206

33.5949047.6067347.3781686.971506

21.6663685.9282675.5111823.51377

0.0828940.09874600.051774

63.318867.3839765.5341225.874839

3.5826290.4882941.398120.568454

131.35122424.50268419.34969517.454208

0000

132.98356521.42587921.5324114.231327

0000

180.75105329.11960531.13253326.047029

00.1622910.037610.015147

4.6434930.15910.8362421.696988

554.61912667.14392865.1229152.22814

0.8091080.03140100.034353

4.1450362.5058010.5939260.680964

6.0698870.8126231.2528360.993157

86.6720958.7515199.69226610.903801

1591.836507249.73247208.638132165.887092

181.70959227.70321821.03581121.825433

6.7558121.7375281.3151841.182105

00.43168300

1.4151480.4343620.5040510.443799

60.8672668.5458516.9963255.287282

2.75150701.8225520.353114

478.40492117.09353674.49383652.641417

00.2424970.1175230

57.4504097.4554845.8700454.655204

120.68806217.59935914.59348711.403895

64.5305299.3211087.4465676.418746

5.2717611.3421090.3813970.879538

00.0103420.0165580

73.4794517.7805986.0459185.548003

2.3892610.4476030.4415120.473712

39.0201464.2499483.6995163.337406

0.2257060.1699040.1050560

3.1936360.6463540.8162570.591831

2.820150.9398070.6133540.286456

14.7467774.9930843.4227233.312075

11.2670583.3305035.2803212.357137

114.64787917.61583216.41023612.493532

32.2728467.5108289.7114275.591041

179.3810939.6575546.60657319.298264

7.7680060.7494041.4413791.734133

187.04623522.12586713.79539814.662264

2.7508910.1954290.487240.187887

117.1350829.19683512.40126311.746226

73.3562528.37268.7807417.59642

157.25648521.59824619.10973713.995058

1.8872470.1967670.3951250.102365

38.8774355.2175373.9940983.221487

87.0654529.9626598.7562248.428485

3.9848351.2281860.6409270.423252

3.4432591.4395381.1609880.52584

2.732161.7005050.1979750

1.8616890.4661290.6639620.358786

283.39458940.40241829.2027928.653422

9.2184462.2286872.2714081.135916

00.0403500.093737

0.4350380.3348990.895870

98.29242110.03979314.84089410.143915

3.8729960.7987871.0405861.234261

824.622294118.73196117.658473114.973906

1.4979260.5373260.7123890.242388

5.6501610.817151.6607941.697885

2.2330940.6003680.4935330.347381

49.3609657.8677299.3244768.369381

97.17058313.86519313.2348810.587905

216.15589530.95783324.81773520.367961

1632.162101203.708233205.33929173.988262

75.0966514.2964539.20436610.369127

19.4750562.4619163.8607061.505022

4.2117581.130581.2608610.55125

285.98339940.86002138.40203731.956909

139.62202112.17098213.16482211.306549

00.1281100

142.1949619.47154119.18543518.530277

12.7134425.4686213.7742313.107263

4.4817493.94281800.882455

000.5104970

8.3836510.1934540.0844670.212843

1175.699535154.326326183.52802117.942665

3.2098667.3675966.0833451.829237

16.1934614.10013920.4559534.764681

46.1168293.7841323.8087384.763049

3.6950250.9409361.8513420.662671

70.9829819.5592178.2014949.147124

1.4359440.1718481.3676310

3.3975460.337120.6300051.393021

4610.661976674.727435576.216108462.563788

52.3045339.0582397.7958.125871

1076.832502164.914015141.264763110.831297

435.77125563.00565163.83553741.971129

63.1323679.2788577.2266835.044669

0.6143650.0819140.2825580.174376

98.711514.8788310.84011411.548826

000.4806590

197.53595926.42564631.83269924.957258

33.3253815.2840465.0809014.25121

33.2139124.3783044.0439963.189019

150.4007124.79398427.21996815.027866

84.57284114.69884712.4868129.373433

1.469080.6033830.4687870.283694

695.739416115.88404193.17556170.718481

300.81356355.17792551.83923252.393364

12.056374.0204775.1013341.694986

3.6246320.2344980.8011460

0.9360440.1097990.196410.054367

16.1651683.0168232.6207411.777108

75.29185812.33490112.5260848.747464

132.3036915.84168812.75884610.122332

0.5490680.1691750.3781490.66594

40.5290496.0650784.6845854.660756

201.56766225.99383220.50480818.051459

1.5761130.4171510.4942260.168338

1.4397410.4437310.1525130.423967

1074.241811150.592699125.114538105.628917

0000

1.9711940.812041.0496812.008339

7.173041.8645661.5887071.024209

4747.884322675.917087570.982102463.590758

1.402620.5259820.2784410.387321

3.0553250.4942960.3997180.454516

13.4885072.3627652.1248981.586163

2.1136771.1529880.543440.617174

207.33694843.6174436.45946933.893177

246.76580426.8645634.64633530.729132

2.6190490.3717081.5131260.288353

3.4582150.620831.1289580.719676

104.83374622.56555512.98361215.113782

3.38470300.592670

0000

3.1248590.5439370.8373960.655933

137.36483215.72087416.20934216.226789

154.52782916.75235616.48739116.500791

9.1486041.8427951.814711.461338

00.01885300

74.08615810.5983998.2836629.746398

0.5313430.0171100.017055

00.4069110.1103780

3.1444780.8982040.7530920.384878

89.87449918.50927317.03505412.91898

0.652381000

111.419666189.184531197.06126440.699914

3.9820340.7117831.1935070.524271

0.337694000.046869

00.256220.1228720

2.0901440.2733650.5395520.359712

127.60818418.29809514.28390813.383968

46.3829149.3803929.5916656.580394

3.4784111.4041150.3501351.096774

406.9728964.99931756.4937147.674691

84.51348614.30001611.9947089.668523

3.1963620.6529750.857430.524094

2.6679351.024960.9172090.765244

0.0692210.0374350.1684850.073863

123.38231319.50488815.55955512.446747

169.96199622.63511524.83803316.007015

74.93159611.95717811.67376310.335974

1.778524000.451332

33.81320711.5749145.8108373.405839

136.14882112.5181513.48623111.692966

13.3108131.799922.0966271.864941

48.9057435.5787166.6402946.554911

4.6402720.8431580.7772750.776776

5.1530871.1619381.5018180.768855

3.5706330.4968410.730570.764242

0.5420030.3462650.8892060.091095

0000

370.35007965.10051656.23169550.44232

1.8282510.3330750.4949880.372041

347.818780.87237989.41185245.935977

0.151796000

68.16889711.0359479.4247477.49159

99.4678738.7711458.6985636.90946

1.7816680.4266880.3711670.509822

1.448980.2593840.2971350.377684

134.00154214.51954414.48241812.491875

1.8793930.4991080.9337070.333227

280.24354734.78856235.18615326.738959

280.24354734.78856235.18615326.738959

44.6952480.3323331.7904572.12852

44.6952480.3323331.7904572.12852

3.19578401.03162.014043

5.4984630.0828820.0172680

36.0010010.2494510.7415890.114477

59.57346413.14673811.0998096.740362

55.9667917.0348695.8284414.978325

0000

0000

55.9667917.0348695.8284414.978325

55.9667917.0348695.8284414.978325

3.6066736.1118695.2713681.762037

2.1586523.2551483.7602421.358246

02.5247413.1145991.096943

0.09355100.1888530

2.0651010.7304070.456790.261303

0000

0.180772000

0.180772000

1.1157852.8567211.5111260.403791

00.38138700.068572

1.1157852.4753341.5111260.335219

0.151464000

0.151464000

0.5621070.1373360.2477440.321198

0.5621070.1373360.2477440.321198

0.5621070.1373360.2477440.321198

0.5621070.1373360.2477440.321198

956.1797491429.3701441299.215167551.756358
0.4994814.6751394.3425731.733

1.3919162.4847320.9645540.824246
955.6802691414.6950051294.872594550.023358

00.14462600

00.14462600

19.2862914.2549858.8873624.46191

0000

0.530203000.059129

0.6063974.2549857.138264.321032

001.5648650

0.358228000

16.45335800.1842370.081749

0000

1.338105000

1.9161180.534240.6036310.265243

1.9161180.534240.6036310.265243

00.56186600

00.56186600

933.0859441406.7145561283.994619544.471959
24.20013452.405762999999114.0745927.143845

106.475421726.088724985.74313442.335221

1.9599169.3112995.9817722.272808

0000

0000

0.6207383.1422881.2154940.348337

0.6126667.8799761.7010480.255011

00.6421100

00.2365840.0571060

02.0017430.9066650.063205

408.94342861.09739548.2406531.809664

00.97714300

0.8500710.0536620.1568620.040664

327.4728548.01463289.15889328.535921

17.8312731.8012992.0546640.951152

00.5685340.1368010.091095

00.1276160.6233990

00.46746200

00.4679662.1670930.37492

00.2594890.1185370

0000

1.7808682.2446110.4616050

3.26451557.62480215.1798032.520921

00.39323200

0.2257130.3622420.1574350

02.6947320.512540

0.0707521.1694570.1526510.118353

32.3610310.50105910.9630873.781684

0.8771015.7416221.1675490.856933

03.8843560.667410.627872

5.3111722.4950411.3432831.907697

0.22833.2238911.0525520.128793

00.83582600.307863

000.4224280

000.4224280

46.2722616.6425834.2855023.806562

46.2722616.6425834.2855023.806562

46.2722616.6425834.2855023.806562

46.2722616.6425834.2855023.806562

1.1995744.3505842.5349932.76935
0.9581824.3505842.5349932.76935

0.241392000

0.241392000

0.241392000

54.636858969.862555310.26220164.179252
0.08113619.5250036.0155331.751061

01.5195080.5510710.184358

01.5195080.5510710.184358

01.5195080.5510710.184358

2.9167070.2642340.2497920

2.9167070.2642340.2497920

2.9167070.2642340.2497920

001.4873180
0.0987656.3937393.6111780.186768

00.90721400

00.90721400

0.0987651.465250.1085630

0.0987651.465250.1085630

00.7585540.1942830

00.7585540.1942830

00.6221280.1717710
03.2627211.8210140.186768

00.1305950.1112150

0000

01.8373880.8883840.186768

00.3094560.2431620

00.1559890.4064820

00.20716500

0.4313831.8945721.9253540.508957

00.9827030.7627940

00.9827030.7627940

00.2879080.2333650.436353
00.2879080.2333650.436353

0000

000.819660

000.819660

00.6239610.1095350.072604

00.39573600

00.2282250.1095350.072604

0.431383000

0.431383000

01.1635812.8223180.323267

01.1635812.8223180.323267

01.1635812.8223180.323267

0.72089111.08987417.2510742.040515

0.72089111.08987417.2510742.040515
0.0690171.0474390.0441350

03.4632768.5637230.61086

0.2397190.9896440.3714860

00.6052570.3375110

00.225530.1808560.209947

01.7811317.1792241.012268

00.9519430.277570

0.022009000

0000.108408

0.3901462.0256540.2965690.099032

43.213715927.518736277.77669358.918071

16.967991322.231632101.52973123.263871
43.213715927.518736277.77669358.918071

00.72169300

0.0540170.5575520.0622460.134881

00.2320470.5949270

01.7959530.2903460

0.1860030.58459700

00.4836770.2033640

00.8551880.03830.19793

01.5982050.06190.025477

00.4680870.3052310

01.3502551.185460

0.0840573.6385920.6170810.599858

01.0886840.1219950

00.9928720.3894890

0.6261216.7466732.1362240.738324

03.5478021.735180.043193

0.11382910.9945913.939371.23845

00.06298200

02.252300

0.0337691.2071210.7263070.028223

0.91482338.09068910.2232881.28896

00.4653270.1415730

1.1996860.5234520.2908650.333686

00.30734500

01.0379220.0833380

00.9229220.0492570

00.167450.5972470

0.0350328.1665731.9113510.388251

00.1513070.2260880

00.8505080.1658690

2.9351040.01452700

01.0447250.5445480

00.2976150.0833380.440585

00.8372480.4479350

0.3794111.6715750.1816630

00.41634400

00.25863200

00.4544710.0958270.083008

0.0377110.6714690.2134350.071418

0.076070.2636920.1011060.031603

00.8603270.1668030

02.3845270.0859410.776978

01.10358200.307863

0.2036830.4587160.0642680

01.28978700

00.091420.5481550

00.3445430.0862750

00.5668370.1842370

00.3610320.0486320

0.0613030.8032820.1803090.0308

19.305105498.176397144.78559828.388393

00.7343710.9780620.22755

00.4850140.0587620.07813

01.403391.0909490.200639

00.4312150.2048230

0000

0000

7.1742610.4933080.0591880.266255

7.1742610.4933080.0591880.266255

00.06961400

2.4104130.0837350.0591880

4.7638480.33995900.266255

0.7828750.0519560.0884410

0.7828750.0519560.0884410

0.7828750.0519560.0884410

0.7828750.0519560.0884410

0.7828750.0519560.0884410

3995.433305603.276427499.964706375.146861

4.1129421.8435946.3109240.958015

4.1129421.8435945.1982510.958015

1.5320780.030880.0856680

1.5320780.030880.0856680

2.5808641.8127145.1125830.958015

0.53995300.5150280

0000

2.0409111.8127144.5975550.958015

001.1126730

001.1126730

001.1126730

3599.56607530.02681436.207869332.946397
100.48271812.853949.784968.589381

70.4673729.056646.9665165.511522

00.06718500

00.06718500

69.3559818.1227036.6428195.381723

0.4117840.8667520.2701060

00.32060800

0.4117840.5461440.2701060

0.69960700.0535910.129799

0.69960700.0535910.129799

67.6643067.7761098.0201344.999761
1668.145876229.493022209.223566156.735428

9.8140210.7025822.070832.673533

1.7579460.4640950.5522470.852894

2.1097030.2384870.3728350.57839

5.94637201.1457481.242249

122.8009677.81277315.2943029.154355

0.96656000

121.8344077.81277315.2440919.154355

000.0502110

294.70548534.75908934.0310923.783321
4.3791670.0977140.0413360

230.15028126.23919827.17038419.14685

60.1760378.4221776.819374.636471

331.19075251.4095143.32121833.982143

2.7153190.42992900.664416

35.0871076.0637855.2974123.243726

0000

01.0078951.6212570.676901

293.38832643.90790136.40254929.3971

83.4824499.9035996.5151745.779136
841.970345127.032959106.48599282.142315

67.3263959.2721458.2537956.413454

522.18855784.28512471.11237554.083396

0.505469000

00.62409300

11.4152082.094811.5668541.298539

120.78727515.90415212.68261810.13037

4.5118080.8809030.8831680.410765

27.2385163.2289294.4893323.461348

4.2204180.7839680.9590130.565307

0.294250.0552360.0236630

60.7554434.9058534.7618095.022355
1416.047568210.12043165.442315128.062035

0.7162710.11168900

0.7162710.11168900

72.7666176.3889858.6698084.158634

72.7666176.3889858.6698084.158634

00.2981710.1161480.048666

00.2981710.1161480.048666

258.99229341.05875933.09451426.501297
26.8890064.0263294.0849753.17219

36.0980535.2818363.5263513.026324

39.4147346.1514834.9278223.892918

101.83436317.76331114.35340211.518247

54.7561377.83586.2019644.891618

0000

986.111496143.902099113.09343488.088824

00.7488430.9077510.513359

833.899063123.64465795.65685174.681087

46.1720877.3079366.0518684.892314

00.2825350.1821640

106.04034611.91812810.29488.002064

36.70544813.4548745.7066024.242259

36.70544813.4548745.7066024.242259

196.93588939.02992225.30849319.777512

33.3256456.9053873.8801513.433791

33.3256456.9053873.8801513.433791

00.36443100

00.36443100

7.6540481.8095272.3335660.863215

7.6540481.8095272.3335660.863215

0000
19.1988592.6634123.8679213.147344

9.8181642.6634123.7610862.699753

0.26729300.0935810.447591

9.11340200.0132540

43.7569017.2317775.0553245.134426

43.7569016.9024385.0553245.134426

00.32933900

0000

0000

93.00043620.05538810.1715317.198736

36.2690612.7945424.894022.648232

51.8997657.2608465.2775114.550504

4.831611000

0000

0000

147.48664729.47285619.48201914.270519

1.0747350.4680870.3052310

1.0747350.4680870.3052310

75.24413313.32530710.8335969.478028

75.24413313.32530710.8335969.478028

0000

52.6878198.90195.8714124.513711

14.0492932.4033511.2098970.608673

38.6385266.4985494.6615153.905038

0.971930.267880.4750710.209951

0.971930.267880.4750710.209951

000.1095350

000.1095350

17.4534386.4172181.7739620.053629

17.4534386.4172181.7739620.053629

0.0545920.0924640.1132120.0152

0.0545920.0924640.1132120.0152

0000

177.79832830.01656229.80624518.795746

177.79832830.01656229.80624518.795746

2.6309181.590780.4641750.474515

0.790709000

00.93617400

1.8402090.6546060.4641750.474515

175.1674128.42578229.3420718.321231

44.3966248.5920446.8206634.913141

01.3511343.8874160.200639

129.52145317.94190917.85225513.207451

1.217380.4648090.7387960

0.0319530.0758860.042940

212.33278940.3984327.15073422.292535

00.506190.143510
98.69496421.51176612.374798.873007

0.1648861.4534890.5182280.223373

0.1648861.4534890.5182280.223373

0.3569060.1040510.2602360.197731
0.7277780.4414670.6815980.59734

0.1704790.1823380.1950830.183833

0.2003930.1550780.2262790.215776

0.2175193.417270.049540
90.33412119.1106211.0314548.052294

35.3367994.5666553.298362.701148

00.5076270.0852860.111175

00.2305090.1376610

00.30312100

54.7798038.7908067.2234155.173879

01.2946320.2371920.066092

7.468179000

7.468179000

113.63782518.88666414.77594413.419528

107.84302516.3607814.56681412.591244
00.8867921.8211430.084711

52.3798977.8328026.1374756.06616

55.4631287.6411866.6081966.440373

3.8785391.79515200.420355

3.8785391.79515200.420355

0.331027000

0.331027000

1.5852340.7307320.209130.407929

1.5852340.7307320.209130.407929

1.6231760.9910310.4889340.154168
0.423212000

0000

0000

0000

00.3031210.1752570

00.3031210.1752570

00.3031210.1752570

1.1999640.687910.3136770.154168

00.45029600

00.45029600

1.1999640.2376140.2274170.154168

1.1999640.2376140.2274170.154168

000.086260

000.086260

0.1575130.278760.1697550.152196

0.1575130.278760.1697550.152196

0.1575130.278760.1697550.152196

0.1575130.278760.1697550.152196

0.1575130.278760.1697550.152196

79.50903111.57332212.453769.228005

79.50903111.57332212.453769.228005

79.50903111.57332212.453769.228005

79.50903111.57332212.453769.228005

00.2718093.5235610.684496

79.50903111.3015138.9301998.543509

0.08740285.5393121.4178480.415659
1942.2974583216.65089288.951543227.298726

0.0529470.34576300

0.0529470.34576300

0.0529470.34576300

0.0529470.34576300

62.08783821.93805811.9540717.402253

60.80941410.5360857.1526474.597191

00.93224100

00.93224100

0.9472220.9410340.5062670

0.9472220.9410340.5062670

1.4078490.9321460.4608880.253387

1.4078490.9321460.4608880.253387

58.4543437.5542586.1854924.343804

58.4543437.5542586.1854924.343804

00.17640600

00.17640600

1.27842411.4019734.8014242.805062
00.87419800

01.27260400

01.27260400

03.2241483.7922491.421515

02.6982343.7922491.421515

00.52591400

00.33328100

00.33328100

00.2589650.2467840

00.2589650.2467840

000.4195880

000.4195880

1.278424000

1.278424000

01.39712600

01.39712600

02.8429650.3428031.383547

00.4899680.3428031.383547

01.29175400

00.42484400

00.63639900

01.19868600

01.19868600

0.44188700.0212240.029512

0.44188700.0212240.029512

0.44188700.0212240.029512

0.44188700.0212240.029512

1257.897262158.810505163.09613150.251513

70.99480611.4481549.95279510.950827

70.99480611.4481549.95279510.950827

70.99480611.4481549.95279510.950827

0.5292556.459120.6039220

06.4591200

06.4591200

0.52925500.6039220

000.6039220

0.529255000

1085.850492123.071241137.91856126.030961

0.1211570.03079900.033556

0.1211570.03079900.033556

1085.729335123.040442137.91856125.997405

1085.729335123.040442137.91856125.997405

62.15575410.7959958.0559546.807985
00.1082650.1086730.02341

60.65297210.3312727.8380396.298993

60.65297210.3312727.8380396.298993

1.5027820.3564580.1092420.485582

00.05208800

1.5027820.304370.1092420.485582

38.3669557.0359956.5648996.46174

0.1071530.1870750.2031290.232385

35.711326.848925.1505885.075459

000.0624720.119021

00.5328300

35.711326.316095.0881164.956438

1.156715000
0000

1.156715000

1.39176701.2111821.153896

1.39176701.2111821.153896

000.1144490

000.1144490

000.1144490

000.1144490

14.6922177.6817121.3144461.066662

14.6922177.6817121.3144461.066662

0.0441220.46382100

0.0441220.46382100

14.6480957.2178911.3144461.066662

14.6480953.6813921.2988671.066662

00.5197920.0155790

00.46418500

02.55252200

484.7030492891.9969686.65766457.290462
1.170343544.6894560000017.6844751.769187

0.2046721159.4405154.3662622.295849
292.2848052155.03725945.49375931.313858

01.3356700
0.34096615.5013331.2400160.129315

011.46358400.057758

01.97203800

0.3409660.7300411.2400160.071557

087.7929080.6454670.092534
113.584126498.00974916.29602412.704294

0.01886418.9392880.0462780

0.32673251.016360.0093510.00897

01.280910.0191810

071.90156999999990.0470730.020981

02.29552300

01.12789900

011.47644700.128981

09.3839660.0167230

01.61832700

01.1375300

00.46405100

02.91023400

33.37670327.4616054.3621583.399757

0154.8074370.1294680

00.65283100

02.15040800

0.38703200.3396410

01.66830700

05.50903400

79.47479524.50998610.6502169.053071

08.03203300

03.2075520.0304680

01.0368800

07.62866300

0116.5136490.215140.054356
178.155041454.4344822.9359816.1844

05.77592900

00.48476500

00.82401500

033.6356100

02.36959800

00.57606500

11.3755441.8861321.5199421.780571

09.5180200

01.5689200

05.92429100.007835

01.53555200

02.70175600

00.41200700

00.47237300

01.03289400

01.08576300

02.32884100

00.39484300

01.22629700

01.89032300

024.23345900.050554

00.46185600

00.75159300

00.3232900

01.26425100

03.43946200

01.20782600

013.18631300

00.58381200

00.80433100

0.1847570.2682720.2211480.019253

00.83724800

60.81267410.6491737.7024324.811706

00.88511300

01.74612300

00.97775500

0000

00.45377500

06.10744400

01.32397900

02.39353600

02.90268800

03.04327500

02.92003600

02.77714200

03.08931700

00.41118400

00.55158700

01.22973200

01.0647020.0714680

04.13556400

03.50769900

03.5301020.0251580

00.28091500

105.78206617.83949612.0044648.804049

01.74568100

00.92931700

00.38039300

02.47013300

0135.6817581.1762280.656076

01.42707700

00.46042800

01.4926700

01.4926700

08.3877500
00.3641700

02.98068200

01.35959900

02.52361500

01.15968400

03.9335900

03.01935200

00.41089600

00.50334200

013.8371720.6554770

01.41771300

05.44279600

00.56713700

05.17384800

00.6712300

000.6554770

00.56444800

0.7413217.8454350.0201840

0.7413217.8454350.0201840

00.59037600

0.74132100.0201840

07.25505900

2.7783590.3276560.4537660.460756

2.7783590.3276560.4537660.460756

2.7783590.3276560.4537660.460756

00.49627400
04.24520600

00.79114600
03.74893200

02.95778600

0000

0000

52.66120248.8143575.88755.095763
00.56123100

00.18819400

00.18819400

02.71735300

02.71735300

02.52615700

02.52615700

52.66120242.8214225.88755.095763

035.332220.1941560

52.6612027.4892025.6933445.095763

9.2004630.6968650.6872950.199465

9.2004630.6968650.6872950.199465
0.9984180.4980840.542110.162796

8.2020450.1987810.1451850.036669

00.5328300

00.5328300

00.5328300

03.87416900

03.05247100

00.93799400

02.11447700

00.82169800

00.82169800

04.08862600

03.71707900

03.71707900

00.37154700

02.3637600.206726
35.03926467.2205744.2458364.758069

05.42615200

05.42615200

00.56876500
02.8801500

02.31138500

00.52021800

00.52021800

35.03926456.0302944.2458364.551343
00.5308330.0479380

35.0392645.597884.1978984.551343

01.48431800

042.99297200

01.2115100

04.21278100

00.33995900
0.3523480.4750310.4674870

000.0462780
0.3523480.1350720.4674870

000.0417010

0.3523480.1350720.3795080

01.78454800

00.53791300

00.53791300

01.24663500

01.24663500

36.8783353.2342194.3313513.58501

36.8783353.2342194.3313513.58501

36.8783353.2342194.3313513.58501

2.9182133.4964181.1121780.747936

2.9182133.4964181.1121780.747936

2.9182131.6374851.1121780.747936

01.85893300

50.67839644.14989316.2509299.360418

00.49176400

00.49176400

01.19388900
50.67839635.45073716.1631519.360418

0.0214580.39319400

00.53190100

00.55105100

00.49460900

46.06806713.7947235.1351974.894441

1.1912780.2395060.5213590.680227

3.03974418.1005579.9272183.685544

0.3578490.1513070.5793770.100206

04.4793290.0877780

02.8371760.0877780

01.64215300

02.07928200

00.9213700

01.15791200

01.64878100

01.64878100

01.4844180.0229040

01.4844180.0229040

01.4844180.0229040

0.13152300.2586330

0.13152300.2586330

0.13152300.2586330

000.2586330

0.131523000

00.4996130.1666750

00.4996130.1666750

00.4996130.1666750

00.4996130.1666750

0000

0000

121.58520349.39448123.51226910.777321
09.5995180.1858040.256031

11.99305323.59626713.6333963.40933
0.53884.1829528.3227140.835647

0.0980833.9939692.3836160.552455
00.0989211.1101830.201227

0000

02.68888900

00.2201190.3252710.13915

00.27420900

0.0980830.2527890.4641090.133948

0000

000.4840530

00.06062400.07813

0000

00.39841800

0.2759092.6671980.3323590.016791

00.54761400

0.1149580.04653100

0000

01.93809500

0.1609510.1349580.3323590.016791

10.4628351.3644051.8658491.654625

00.28254400

10.4628351.0818611.8658491.654625

0.6174269.0178690.7288580.349812
01.87629900

0000

03.30197900

0.5575793.5057560.2449370.136589

0.0598470.0717380.0201840

0000.213223

000.4637370

00.26209700

02.36987400

00.47488800

01.89498600

109.5921512.075579.6266927.11196

109.5921512.075579.6266927.11196

109.5921512.075579.6266927.11196

01.27176100
03.3555670.0663770

01.2385890.0663770

00.37789100

00.1861480.0663770

00.41341500

00.26113500

00.84521700

00.84521700

00.76755900

00.76755900

00.76755900

0.618130.4444860.4381340.065344

0.618130.4444860.4381340.065344

0.618130.4444860.4381340.065344

0.618130.4444860.4381340.065344

278.91077332.28433932.38676422.345211

278.91077332.28433932.38676422.345211

278.91077332.28433932.38676422.345211

278.91077332.28433932.38676422.345211

278.91077332.28433932.38676422.345211

0.0481380.1967330.0851250.060163

0000

00.20311600

43.2364646.8515955.3722894.426515

00.62149400

235.62617124.41140126.9293517.858533

04.8743389.0408593.368151

04.8743389.0408593.368151

04.8743389.0408593.368151

04.8743389.0408593.368151

04.8743389.0408593.368151

04.8743389.0408593.368151

10.668524251.114986104.102016418.038309

01.8318453.7938392.50994

01.8318453.7938392.50994

01.8318453.7938392.50994

01.8318453.7938392.50994

01.8318453.7938392.50994

10.668524249.283141100.308177415.528369

10.668524249.283141100.308177415.528369

10.668524249.283141100.308177415.528369

10.668524249.283141100.308177415.528369

10.668524249.283141100.308177415.528369

000.3222540.213448

000.3222540.213448

000.3222540.213448

000.3222540.213448

000.3222540.213448

000.3222540.213448

15.844423258.338575318.29605220.549079

1.16705125.5403246.0568941.811028

1.16705125.5403246.0568941.811028

00.8529690.5243420

00.8529690.5243420

00.8529690.5243420

0000

0000

0000

0000

0000

0000

1.16705124.4017845.3426861.811028

1.16705124.4017845.3426861.811028

1.16705124.4017845.3426861.811028

00.2855710.1898660

00.2855710.1898660

0000

00.2855710.1898660

2.718117.69270726.8942739.467948

2.718117.69270726.8942739.467948

2.1814753.956797.1444232.248233

0.011923.9412257.0086812.248233

0.011923.9412257.0086812.248233

2.105180.0155650.1357420

2.105180.0155650.1357420

0.064375000

0.064375000

0.11328000
0.53662513.73591719.749857.219715

03.1357714.9872132.480767

03.1357714.9872132.480767

0000

0000

0.4233455.7315835.7923641.125265

0.4233455.7315835.7923641.125265

02.9192485.6055092.594338

02.9192485.6055092.594338

01.9493152.9707421.019345

01.9493152.9707421.019345

000.3940220

000.3940220

4.7096150.4315780.1574210.025559

4.7096150.4315780.1574210.025559

3.408030.4315780.1574210.025559

3.408030.4315780.1574210.025559
00.3790260.1148990

3.408030.0525520.0425220.025559

1.301585000

1.301585000

1.301585000

02.9446174.7548761.294712

02.9446174.7548761.294712

02.9446174.7548761.294712

02.9446174.7548761.294712

02.9446174.7548761.294712

7.249657211.729349280.432586207.949832

7.249657211.729349280.432586207.949832

6.547669211.729349280.369297207.949832

6.547669211.729349280.369297207.949832

6.547669211.729349280.369297207.949832

0.70198800.0632890

0.70198800.0632890

0.70198800.0632890

00.3149090.1842370

00.3149090.1842370

00.3149090.1842370

00.3149090.1842370

00.3149090.1842370

00.3149090.1842370

0.02700716.71464630.67109211.38336

0.02700716.71464630.67109211.38336

0.02700716.71464630.67109211.38336

0.02700716.71464630.67109211.38336

0.02700716.71464630.67109211.38336

0.02700716.71464630.67109211.38336

01.764060.3093964.046782

01.764060.3093964.046782

01.764060.3093964.046782

01.764060.3093964.046782

01.764060.3093964.046782

01.764060.3093964.046782

771.299647217.02859147.025938163.472443

1.5127934.7913761.018380.116872
718.461424210.200744141.57143488.851511

0.49637711.9362371.9893570.419893

0.49637711.9362371.9893570.419893

0.49637711.9362371.9893570.419893

0.49637711.9362371.9893570.419893

487.49117971.85014164.97861743.975252

156.79503416.21290511.87657111.81528

156.79503416.21290511.87657111.81528

1.8627840.6364650.6858610.359662

154.9322515.5764411.1907111.455618

2.0189380.2602320.6076350.291794

2.0189380.2602320.6076350.291794

2.0189380.2095580.3671130.291794

00.0506740.2405220

0000

328.67720755.37700452.49441131.868178
02.2637681.0028470

48.99728721.94776725.2181658.651444

48.99728721.94776725.2181658.651444

00.3556940.0353950

279.6799230.80977526.23800423.216734

00.5794640.2251870.048406

0000

01.30997500

70.0825318.8090737.6957165.736699

209.59738920.11126318.31710117.431629

01.3897250.3213390.085611

01.3897250.3213390.085611

00.53782300

00.53782300

00.8519020.3213390.085611

00.8519020.3213390.085611

5.25103532.14822237.19977715.973341

5.25103532.14822237.19977715.973341

0.2313437.0392361.9820551.905548

0.2313437.0392361.9820551.905548

0.31728211.37241114.0532834.764802
5.01969225.10898635.21772214.067793

0.20452910.16245514.3672427.007261

0.10333100.2463020

4.269330.7601871.4332530.576922

0.125220.2149050.1736390.026177

00.2212770.3829650.125021

02.3777514.5610381.56761

43.6605276.358945.2463044.585301

43.6605276.358945.2463044.585301

0.2796970.2390040.228330.068766

0.2796970.2390040.228330.068766

43.380836.1199365.0179744.516535

43.380836.1199365.0179744.516535

0.1471252.958210.564390.266989

0.1471252.958210.564390.266989

0.1471252.958210.564390.266989

0.1471252.958210.564390.266989

8.2490660.1764060.6156240

8.2490660.1764060.6156240

8.2490660.1764060.6156240

8.2490660.1764060.6156240

47.2162018.3120654.8984275.692181

47.2162018.3120654.8984275.692181

47.2162017.7452284.8984275.692181

00.56683700

0.8043633.8509290.25790
124.43712170.27942224.73921917.736071

45.55211410.1278477.1000435.235556

00.27701200

00.27701200

000.5029650.119887

000.5029650.119887

45.3736056.5673985.6134265.04142

45.3736056.5673985.6134265.04142

0.1785093.2834370.9836520.074249

0.1785093.2834370.9836520.074249

0.0673530.6057230.043130
78.08064455.96619617.38127612.500515

01.4540850.1840820

00.928190.1840820

00.52589500

01.650990.6956050

01.650990.6956050

00.0557420.1065840

00.0557420.1065840

63.07286110.4856098.4952417.514861

63.07286110.4856098.4952417.514861

0.0916377.1275650.7752660.537909
14.22354835.0170175.7612824.161055

00.5639150.1080420

01.2842200

0.5790770.2327130.119240.079311

0.2996840.575370.1914830

00.1439540.1786140.17528

00.3668720.0399340.430469

11.1578492.47533401.061545

00.0390410.0701250

00.2755290.0525790.278184

00.39978600

00.20198800

00.18112100

03.698570.6798630.363135

01.00876100.197749

02.886770.7403530.83001

00.51046500

02.9305520.7175670

00.58381200

00.7542190.048250

00.28890100.040785

2.0953016.1654371.6488320.166678

02.3221220.3911340

00.2889010.1281790

0.2313430.57326800

0.2313430.57326800

0.2672522.5113610.8540560.431883

00.3683450.119240.237933

0.2672522.1430160.7348160.19395

0.2182873.1460621.1131170.392716

0.2182873.1460621.1131170.392716

00.17743800

00.17743800

00.3344500

00.3344500

52.8382236.8278465.45450474.620932

52.8382236.8278465.45450474.620932

52.8382236.8278465.45450474.620932

52.8382236.8278464.5835395.609178

52.8382236.8278464.5835395.609178

000.87096569.011754

000.87096569.011754

044.73898761.49064822.021136

044.73898761.49064822.021136

021.58362930.28480710.526704

021.58362930.28480710.526704
011.09026314.8958915.468609

02.9460444.8744481.826629

02.9460444.8744481.826629

03.2845744.5113631.444877

03.2845744.5113631.444877

04.2627486.0031051.786589

04.2627486.0031051.786589

023.15535831.20584111.494432

013.03419415.2858435.519955

013.03419415.2858435.519955

013.03419415.2858435.519955

010.12116415.9199985.974477

010.12116415.9199985.974477

03.049495.0387881.660009

07.07167410.881214.314468

0.362101000

0.362101000

0.362101000

0.362101000

0.362101000

0.362101000

82.45642413.80549819.5535828.250353

82.45642413.80549819.5535828.250353

82.45642413.80549819.5535828.250353

82.45642413.80549819.5535828.250353

82.45642413.80549819.5535828.250353

82.45642413.80549819.5535828.250353

00.42992900

01.4048112.6013790

11.4404910.1399080.4711770.950323

63.7206427.424445.8739656.094532

0000

7.2952914.4064110.6070611.205498

9189.223509490583.667994001504344.587520999528465.843228
12.230056604.718818917.831779536.264929

2607.451839251801.324450001380122.817387999172905.820202
2.030784135.97064211.2421384.554547

2602.210871251230.978596001379230.038755999172508.425228

2580.511888249864.868715001377249.930322999171541.929881
37.7638074006.5592136304.3363482754.332834

6.9041871097.4185581660.842531675.563751

6.9041871097.4185581660.842531675.563751

6.9041871097.4185581660.842531675.563751

1.398224109.133714200.58326473.082563

1.398224109.133714200.58326473.082563

1.398224109.133714200.58326473.082563

2426.753273234350.836833001353236.606844999160956.749354
021.47276722.86412713.089935

548.80804155538.577652999984886.998046000138613.002967
2426.753273234329.364066001353213.742717999160943.659419

0.10190618.37007622.25198610.870472

232.94309725201.17768338729.333331999917542.853967

0.0595783.5275024.1348842.08428

54.001551723.2688391812.1557281352.582689

100.1537219276.30388514349.7163436664.555581

41.3742462803.3491024129.9329012060.610391

1449.311134139764.789326001209279.21949799994697.0990719996

16.5485751359.1274122101.375525962.133281

16.5485751359.1274122101.375525962.133281

16.5485751359.1274122101.375525962.133281

4.653543380.251881597.938281247.358166

4.653543380.251881597.938281247.358166
0.43103861.90304592.17073129.539012

1.876527184.156929272.253787121.05771

2.345978134.191907233.51376396.761444

0000

0000

0.1415035.6326237.3196572.223391
8.0984341011.4349831536.160419658.854864

1.997457284.799412430.913802195.152399

1.997457284.799412430.913802195.152399

0.37061526.90480558.88682119.88991
5.959474721.0029481097.92696461.479074

0.566662189.079106274.102593127.842033

5.022197505.019037764.937546313.747131

78.3918457550.10612111612.087115213.855068
2.571987250.734396392.928247176.82504

48.6336354715.817617090.6531183191.15565

48.6336354715.817617090.6531183191.15565

27.1862232583.5541154128.5057451845.874378
4.232638428.05502718.578982293.419696

5.183344401.747624605.266974275.644046

9.703294865.1528811368.899243606.090636

8.066947888.598591435.760546670.72

21.6989831366.1098811980.108433966.495347

1.727045181.29009282.167234128.86181
02.1474213.0236341.323308

0.01050961.682008101.48544737.778241

036.4790562.11860322.598446

036.4790562.11860322.598446

036.4790562.11860322.598446

0.01050925.20295839.36684415.179795

0.01050925.20295839.36684415.179795

0.01050925.20295839.36684415.179795

1.716536117.460661177.65815389.760261

1.716536117.460661177.65815389.760261

016.82000729.23046411.296534
1.716536113.136723169.14497785.528911

0.02654334.40008354.11725721.848428

01.4942522.5023680.853273

1.68999360.42238183.29488851.530676

04.3239388.5131764.23135

04.3239388.5131764.23135

0.862311187.151151297.073763134.545772

0.862311187.151151297.073763134.545772

012.29131722.69978410.181288

012.29131722.69978410.181288

012.29131722.69978410.181288

0.862311174.859834274.373979124.364484
016.07921226.04040411.135648

050.6427579.18923139.870397

050.6427579.18923139.870397

0.862311108.137872169.14434473.358439

0.862311108.137872169.14434473.358439

0.62082865.933973102.29550549.432845

0.62082865.933973102.29550549.432845

0.62082865.933973102.29550549.432845

0.33573728.91349141.76358319.12487

0.33573728.91349141.76358319.12487

027.41671239.89014619.30827

027.41671239.89014619.30827

0.2850919.6037720.64177610.999705

0.2850919.6037720.64177610.999705

0.277242114.172488105.990081201.849659
689.487304000002195606.313455106451.515134332400.294827001

043.64558336.19513424.037465

043.64558336.19513424.037465

043.64558336.19513424.037465

043.64558336.19513424.037465

043.64558336.19513424.037465

0000.088752
33.443785118.1862063701.3296796669.611713

010.76599616.7877246.748455

010.76599616.7877246.748455

010.76599616.7877246.748455

010.76599616.7877246.748455

0.22853856.72217482.59198130.816642

0.22853856.69591282.59198130.761121
0.2285386.74627413.9322784.288462

049.94963868.65970326.472659

049.94963868.65970326.472659

00.02626200.055521

00.02626200.055521

00.02626200.055521

33.2152425050.6980363601.9499746631.957864

0000

0000

0000

33.2152425050.6062273601.9499746631.824666

0.1034295.0542689.1516284.59888
30.6358953756.907173163.0968894768.269578

4.427805985.905029534.7041251726.813201

0000

03.2621314.4758472.916965

25.7091712751.5881742594.052623024.089031

0000

04.7209129.1739983.697074

0.395490.1202400.424469

06.25641611.5386715.729958

2.5793471293.699057438.8530851863.555088

00.05103100.126339

2.5793471293.538037438.8530851863.428749

00.10998900

00.09180900.133198

00.09180900.133198

00.09180900.133198

0.621411109.144345164.6300471.591718

0.621411109.144345164.6300471.591718

0.621411109.144345164.6300471.591718

0.621411109.144345164.6300471.591718

0.621411109.144345164.6300471.591718

2.6646962.1601780.7913411.595043

2.6646962.1601780.7913411.595043

00.06536100

00.06536100

00.06536100

2.6646962.0948170.7913411.595043

2.5544910.9392570.7913410.787272

2.5544910.9392570.7913410.787272

0.1102051.1555600.807771

0.1102051.1555600.807771

549.400526000002186113.13172100464.850552318488.475720001

549.400526000002186113.13172100464.850552318488.475720001

0.02738693.64692445.269101160.948036

0.0273860.21748900.468347

0.0273860.21748900.468347

093.42943545.269101160.479689

093.42943545.269101160.479689

6.4092593460.7837241292.2441294720.797934
474.677622000002179041.2307996521.7785639997280852.464682001

1.2641871593.59286567.3796172293.13479

0.4460321247.525602478.4114131792.465029

0.818155346.06725888.968204500.669761

462.643685000002168574.48155492344.6358219997265943.036675001
3.3764941008.46081565.5679181607.351009

31.83096811512.502846304.93392418219.521925

424.890995000002153214.11711284353.0338249998241660.954002001

2.5452282839.4007921121.1001554455.209739

4.27024692.1427822054.2041876946.681034
0.7740731959.332412735.1179622615.924608

0001.467259

0.683363896.817098443.0905171391.866193

0.4135191104.708558481.8473641739.738433

2.399245731.284714394.1483441197.684541

0.090291720.22987263.314809948.814249

0664.064439294.039878931.116783
0.490868873.282649547.3260735055.989548

0.11285280.55416643.424181124.822228

034.4240720.53248348.891798

0.11285246.13009622.89169875.154116

0000

0000.776314

000.4074965.653926
0.37801686.43796187.1957223933.779393

0.37801686.38523185.6416983872.678802

000.74338437.369598

00.052730.40314418.077067

042.22608422.66629266.271144

0000

042.22608422.66629266.271144

8.840811706.256041222.6497464214.554584

8.840811706.256041222.6497464214.554584

8.840811706.256041222.6497464214.554584

01.346490.39927681.546192
8.0993061819.719563705.07107223203.06297

05.2602731.1567628.145744

05.2602731.1567628.145744

00.33017801.948105

00.33017801.948105

00.6574170.1351981.348749
2.27787961.97807369.16307440.215359

2.27787960.32498169.01318838.58204

00.9956750.0146880.28457

01.88623205.586285

01.88623205.586285

0000

0000

5.6353771710.634376617.11915822925.605964
0.74083165.0022621.4719411327.039188

00.38510906.199255

1.341943320.894357150.474344714.033967

1.062349479.98923499999968.19791318970.787526

01.3638280.10842442.324509

03.4797970.641515141.643172

0000.588303

0000.881139

1.465085822.803089373.5845311540.755772

1.02516912.7858872.25909326.702488

03.9308140.381397153.831705

0000.81894

0.1860519.1149089.832611100.137872

0.1860519.1149089.832611100.137872

00.222923013.124678
00.0749402.166289

00.14798306.486622

0004.471767

0000
018.946117.40019125.850108

00.0390840.1296870.296698

0000.031854

018.9070267.27050425.521556

0000.902663

0000.902663

0000

0000

0000

0000

34.017121819.762823216.4367911102.944008
0.2486826.4485790.1577377.142137

0.440052001.291406

0.440052001.291406

32.067396292.9716649.808213339.386920000001

32.067396292.9716649.808213339.386920000001

1.260991520.34258206.470841755.123545
015.90737114.23694338.823865

0.167493491.133425191.920494694.480827

1.09349813.3017840.31340421.818853

0000

0000

0000

0000

4.125611017.581192706.6142872670.854923
0.10184118.6763059.44735334.843511

033.57115926.13131989.53709

033.57115926.13131989.53709

062.57643839.03561390.442962

010.67056.34157522.993052

051.90593832.69403867.44991

033.61193220.27457253.018675

032.83923320.27457252.370938

00.77269900.647737

022.58839211.37995543.86845

022.58839211.37995543.86845

4.003494790.14252558.9385052218.427058

4.003494790.14252558.9385052218.427058

0.0202755.5213852.8602785.393738

0.0202755.5213852.8602785.393738

0000.685423

0000.685423

00.166363027.660926
0004.449293

00.078722023.138774

00.08764100.072859

050.72669838.546692106.97709

050.72669838.546692106.97709

3.50566827.43109724.17368626.765089

00.19632800.584616

00.19632800.584616

3.50566827.23476924.17368626.180473

0.0455720.12410600.114343

3.46009627.11066324.17368626.06613

049.81219918.61932354.77541

049.81219918.61932354.77541

049.81219918.61932354.77541

0000.316642
15.616135664.408443456.9119091146.11647

3.11487349.895947332.688888677.983514

3.11487349.895947332.688888677.983514

12.501265314.512496124.223021467.816314
00.4420880.0174310.278704

0.0352836.69444916.53633353.560256

0000

12.433634190.21879469.162035267.487879

0.03235139.29927315.7897178.2264

047.85789222.71751267.955212

0000.307863

96.457768207.096468159.36584434.501842
00.01422600.05572

0000.137085
43.99798332.9515638.60054522.823562

0.0209350.44931800.098962

0.0209350.44931800.098962

0.0209350.44931800.098962

0000

0000

00.65285800.353114

00.65285800.353114

00.65285800.353114

25.69306228.36836736.42735120.940845

00.67266200
25.69306228.36836736.42735120.940845

017.42605228.64908314.071217

0.0998361.51920200.520747

25.1197964.1337473.8794464.21717

02.6240353.8988221.541266

0.473431.99266900.590445

18.2839863.4810172.1731941.293556

18.2839863.4810172.1731941.293556

18.2839863.4810172.1731941.293556

0000.685423

0000.685423

0000.685423

0000.685423

50.6276237.0924446.0343484.872008

50.6276237.0924446.0343484.872008

50.6276237.0924446.0343484.872008

50.6276237.0924446.0343484.872008

1.43277150.31366690.336053391.650824

1.43277149.19555186.505723390.82666

1.356344148.70370386.408458390.517204

0.0764260.4918480.0972650.309456

00.3371200.141204

0.0764260.1547280.0972650.168252

01.1181153.830330.824164

01.1181153.830330.824164

01.1181153.830330.824164

03.1606495.1387924.291387

02.8161065.1387922.315481

02.8161065.1387922.315481

02.8161065.1387922.315481

00.34454301.975906

00.34454301.975906

00.34454301.975906

00.07277100.041167

0000

0000

0000

00.07277100.041167

00.07277100.041167

00.07277100.041167

0.39939213.49115219.25610210.081751

0.37192810.39640214.4759767.78466

0.37192810.39640214.4759767.78466

0.37192810.39640214.4759767.78466

0.0274643.094754.7801262.297091

0.0274643.094754.7801262.297091

0.0274643.094754.7801262.297091

0000

0000

0000

0000

00.03989500.12145

00.03989500.12145

00.03989500.12145

00.03989500.12145

00.03989500.12145

6.6218813840.460071727.8146376458.13319

6.6218813840.460071727.8146376458.13319

019.88101427.93915226.171658

012.74248219.9300613.847748

012.74248219.9300613.847748

012.74248219.9300613.847748

012.74248219.9300613.847748

01.45980402.77991
06.9621268.0090926.032354

02.7790974.0472611.473363

02.7790974.0472611.473363

02.7790974.0472611.473363

02.1965513.9618311.779081

02.1965513.9618311.779081

02.1965513.9618311.779081

00.49506700

00.49506700

00.49506700

00.03160700

00.03160700

00.03160700

00.17640606.291556

00.17640606.291556

00.17640606.291556

00.17640606.291556

024.99905348.1407317.778361

0000.389367

0000.389367

000.4448080.022678

000.4448080

000.4448080

000.4448080

0000.022678

0000.022678

0000.022678

024.99905347.69592217.366316
011.26376719.9540178.27143

0000

0000

0000

09.15987117.5192863.626372

09.15987117.5192863.626372

09.15987117.5192863.626372

04.57541510.2226195.468514

04.57541510.2226195.468514

04.57541510.2226195.468514

013.39643514.4679486.04213

013.39643514.4679486.04213

013.39643514.4679486.04213

013.39643514.4679486.04213

013.39643514.4679486.04213

0000.384878

0000.384878

0000.384878

0000.384878

0000.384878

04.49492912.0323766.777795

04.49492912.0323766.777795

04.49492912.0323766.777795

04.49492912.0323766.777795

04.49492912.0323766.777795

04.49492912.0323766.777795

11.39738752.23917871.52268330.27419

0.2541399.31233110.817175.902319

0.2541399.31233110.817175.902319

0.2541399.31233110.817175.902319

0.2541399.31233110.817175.902319

0.2541399.31233110.817175.902319

11.1221441.8722191.5992191.61645

11.1221441.8722191.5992191.61645

11.1221441.8722191.5992191.61645

11.1221441.8722191.5992191.61645

11.1221441.8722191.5992191.61645

03.9742857.2740253.379793

03.9742857.2740253.379793

03.9742857.2740253.379793

03.9742857.2740253.379793

03.9742857.2740253.379793

0.02110415.93807919.2469236.616008

0.02110415.93807919.2469236.616008

0.02110415.93807919.2469236.616008

0.02110415.93807919.2469236.616008

0.02110415.93807919.2469236.616008

021.14226432.58534612.75962

021.14226432.58534612.75962

021.14226432.58534612.75962

021.14226432.58534612.75962

021.14226432.58534612.75962

1.08312212.71343322.1981439.361066

010.61297817.3497338.899269

010.61297817.3497338.899269

06.1774379.2970424.79756

06.1774379.2970424.79756

06.1774379.2970424.79756

04.4355418.0526914.101709

04.4355418.0526914.101709

04.4355418.0526914.101709

1.0831222.1004554.848410.461797

1.0831222.1004554.848410.461797

1.0831222.1004554.848410.461797

1.0831222.1004554.848410.461797

1.0831222.1004554.848410.461797

04.0460086.729663.344309

00.04430300.045594

00.04430300.045594

00.04430300.045594

00.04430300.045594

00.04430300.045594

00.37199500

00.37199500

00.37199500

00.37199500

00.37199500

03.629716.729663.298715

03.629716.729663.298715

03.629716.729662.829113

03.629716.729662.829113

03.629716.729662.829113

0000.469602

0000.469602

0000.469602

4914.02183340509.72110414864.93429921005.133274

1.31311342.26414273.50595823.854942

1.31311342.26414273.50595823.854942

1.31311342.26414273.50595823.854942

1.31311342.26414273.50595823.854942

1.31311342.26414273.50595823.854942

06.88832310.9558034.921099

06.88832310.9558034.921099

06.88832310.9558034.921099

04.1272796.2573283.049238
06.88832310.9558034.921099

02.7610444.6984751.871861

4912.7087240450.90713214770.96031220973.359762
9.60458990.5240575.1367540.023324

14.472225346.271324193.537309538.742445

14.472225346.271324193.537309538.742445

12.68778323.770609168.004379527.018937

12.68778323.709218168.004379526.860315

00.06139100.158622

011.09990315.557975.389026

1.78444511.4008129.974966.334482

0.5951026.621025.4573224.16205

1.1893434.7797924.5176382.172432

9.74807944.91210741.4138517.7404

9.74807944.91210741.4138517.7404

8.37747737.99942135.38166515.53732

8.37747737.99942135.38166515.53732

1.3706026.9126866.0321852.20308

1.3706026.9126866.0321852.20308

38.38458617.27279813.7058610.859115

37.8330995.5247695.2078434.67013

37.8330995.5247695.2078434.67013

37.8330995.5247695.2078434.67013

0.1932423.4927870.7538892.638224
00.0910730.1148990.049482

0.1932423.4017140.638992.588742

0.1932423.4017140.638992.588742

0000

0000

0.3582458.2552427.7441283.550761

0.3582458.2552427.7441283.550761

0.3582458.2552427.7441283.550761

2.92600218.5461216.9234887.731151

2.92600218.5461216.9234887.731151

0000.261081

0000.261081

2.55339410.929984.8254161.985305

2.55339410.929984.8254161.985305

0.3726087.6161412.0980725.484765

0.3726087.6161412.0980725.484765

0.57498829.25773921.21556922.43605

0.10137712.4428086.38202317.51413

0.10137712.4428086.38202317.51413

0.10137712.4428086.38202317.51413

0.47361116.81493114.8335464.92192

0.47361116.72456214.8335464.903138

0.47361116.72456214.8335464.903138

00.09036900.018782

00.09036900.018782

1.5502614.36491215.4904135.672774

1.5502614.36491215.4904135.672774

1.5502614.36491215.4904135.672774

1.5502614.36491215.4904135.672774

1.87834942.06138820.17545143.0553

1.87834942.06138820.17545143.0553

1.87834942.06138820.17545143.0553

1.87834942.06138820.17545143.0553

04.90647219.0489755.596092

04.90647219.0489755.596092

04.90647219.0489755.596092

04.90647219.0489755.596092

1.99628821.78043215.4433256.629156

04.892485.5282491.346811

04.892485.5282491.346811

04.892485.5282491.346811

1.99628816.8879529.9150765.282345

1.99628816.8879529.9150765.282345

1.99628816.8879529.9150765.282345

2.70967920.30046720.1142047.50684

2.70967920.30046720.1142047.50684

1.56356215.64509815.7472965.483574

1.56356215.64509815.7472965.483574

1.1461174.6553694.3669082.023266

1.1461174.6553694.3669082.023266

8.79605865.28099455.63151729.074182

01.8933340.9793082.170959

01.8933340.9793082.170959

01.8933340.9793082.170959

8.79605863.11162554.24069526.903223

8.79605863.11162554.24069526.903223

8.79605863.11162554.24069526.903223

00.2760350.4115140

00.2760350.4115140

00.2760350.4115140

37.873993281.709909210.302941136.15332

37.873993281.709909210.302941136.15332
0.52894512.52572717.98285512.25477

0.30678316.59892814.0667053.043369

0.30678316.59892814.0667053.043369

1.4887036.4592144.5004330.882082

1.4887036.4592144.5004330.882082

1.2984721.98262915.74356810.562713

1.2984721.98262915.74356810.562713

0000

1.03079510.2865056.8151213.731134

1.03079510.2865056.8151213.731134

14.75759410.5849557.383462.550188

14.75759410.5849557.383462.550188

0.694739.3429915.535215.836707

0.694739.3429915.535215.836707

6.72908150.66409244.40643824.293666

6.72908150.66409244.40643824.293666

11.038892143.26486893.86915172.998691

11.038892143.26486893.86915172.998691

1057.089668267.645032181.675633174.779114

1057.089668267.645032181.675633174.779114

2.22383129.22671811.8782820.46835

1.0167584.8706034.2373712.510707

1.20707324.3561157.64090917.957643

4.14054578.17036130.6732345.151722

4.14054578.17036130.6732345.151722

1050.725292160.247953139.124123109.159042

00.8033620.4496930.14331

1050.725292159.444591138.67443109.015732

7.80951240.20338237.20367215.368886

7.80951240.20338237.20367215.368886

7.80951240.20338237.20367215.368886

7.80951240.20338237.20367215.368886

20.64988977.92990651.18260228.154531

20.4437370.36629945.06290126.288964

1.58470119.999689.5623246.532031

1.58470119.999689.5623246.532031

17.96050237.88783230.28957216.672948

17.96050237.88783230.28957216.672948

0.89852712.4787875.2110053.083985

0.89852712.4787875.2110053.083985

0.2061597.5636076.1197011.865567

0.2061597.5636076.1197011.865567

0.2061597.5636076.1197011.865567

3033.0612538931.63770613656.74251619802.878176

98.1943875.283874728.085336349.819707
3031.19050638910.55033113644.68280919795.147274

4.29866751.20403441.10282218.572045

0.82495115.2101427.2473153.312962

1.396814.19868412.253067.774676

2.07691621.79520821.6024477.484407

2.62126830.38503322.99407611.963814

2.62126830.38503322.99407611.963814

2.13616327.3547617.7985766.328262

2.13616327.3547617.7985766.328262

0.72919719.21412314.1926834.996575

0.72919719.21412314.1926834.996575

15.566868157.448526146.16017965.35217

02.4774020.6231833.073876

15.566868154.971124145.53699662.278294

01.0778170.3492980.381286

01.0778170.3492980.381286

2862.25376736744.29194312028.99531618835.056531
64.145413610.795868532.775292249.240591

5.42194239.87600935.16700218.825917

00.5205910.395950

00.42648500

0.62354227.1346734.7915657.336385

2784.49591235970.23151711404.44940718525.334268

1.37641910.2219225.9863433.516815

00.332120.1240290.086951

0.3957979.9645712.2775756.023919

1.0186678.1448091.9123243.822178

4.77607566.64337841.11582920.869507

1.94816227.1556717.7597359.727378

1.94816227.1556717.7597359.727378

0.467515.2919613.9679622.182117

0.467515.2919613.9679622.182117

16.415771603.259796413.401438303.391935

16.415771603.259796413.401438303.391935

1.81386127.66848821.63032812.908781

1.81386127.66848821.63032812.908781

24.744972340.914306188.24506174.466673

24.744972340.914306188.24506174.466673

1.87074421.08737512.0597077.730902

1.87074421.08737512.0597077.730902

1.87074421.08737512.0597077.730902

05.09382611.3143063.111906

05.09382611.3143063.111906

05.09382611.3143063.111906

05.09382611.3143063.111906

2.2706290.985010.9278910.514918

2.2706290.985010.9278910.514918

2.2706290.985010.9278910.514918

2.2706290.985010.9278910.514918

660.634031105.14349886.08752570.538708

660.634031105.14349886.08752570.538708

660.230296101.27633583.32759869.337347

660.230296101.27633583.32759869.337347

0.4037353.8671632.7599271.201361

0.4037353.8671632.7599271.201361

08.24709417.8324924.774965

08.24709417.8324924.774965

08.24709417.8324924.774965

08.24709417.8324924.774965

0.67864516.8329669.8540232.018409

0.67864516.8329669.8540232.018409

0.67864516.8329669.8540232.018409

09.6615079.5122262.997471

09.6615079.5122262.997471

09.6615079.5122262.997471

09.6615079.5122262.997471

09.6615079.5122262.997471

0.80923813.35863715.4350648.553102

0.80923813.35863715.4350648.553102

0.80923813.35863715.4350648.553102

0.80923813.35863715.4350648.553102

0.80923813.35863715.4350648.553102

0.80923813.35863715.4350648.553102

02.5992263.8331051.616727

02.5992263.8331051.616727

02.5992263.8331051.616727

02.5992263.8331051.616727

02.5992263.8331051.616727

010.3611916.0870896.498248

010.3611916.0870896.498248

010.3611916.0870896.498248

010.3611916.0870896.498248

010.3611916.0870896.498248

010.3611916.0870896.498248

0.04298212.78719718.9085619.032215

0.04298212.78719718.9085619.032215

0.0429820.4671670.1699340.222969

0.0429820.4671670.1699340.222969

0.0429820.4671670.1699340.222969

0.0429820.4671670.1699340.222969

012.3200318.7386278.217189

012.3200318.7386278.217189

04.0426265.6087442.89118

03.1570625.6087442.389039

00.88556400.502141

08.27740413.1298835.326009

08.27740413.1298835.326009

0000.592057

0000.592057

0000.592057

0000.592057

0.37097929.56407648.09291824.165261

0.0434552.8587414.0158261.6394

0.0434552.8587414.0158261.6394

0.0434552.8587414.0158261.6394

0.0434552.8587414.0158261.6394

0.0434552.8587414.0158261.6394

010.04073719.7415749.183036

010.04073719.7415749.183036

010.04073719.7415749.183036

010.04073719.7415749.183036

010.04073719.7415749.183036

0.32752416.66459824.33551813.342825

0.32752416.66459824.33551813.342825

05.3564213.149364.293376
0.32752416.66459824.33551813.342825

0.32752411.30817811.1861589.049449

0.32752411.30817811.1861589.049449

026.33762241.42091520.556401

026.33762241.42091520.556401

026.33762241.42091520.556401

02.5143543.2154581.702254

02.5143543.2154581.702254

02.5143543.2154581.702254

023.82326838.20545718.854147

023.82326838.20545718.854147

023.82326838.20545718.854147

0.12873925.81550938.76255319.015309

0.12873925.81550938.76255319.015309

0.12873925.81550938.76255319.015309

0.1287395.6171918.3528784.243819

0.1287395.6171918.3528784.243819

0.1287395.6171918.3528784.243819

020.19831830.40967514.77149

020.19831830.40967514.77149

020.19831830.40967514.77149

05.98764510.9771235.558028

05.98764510.9771233.302941

05.98764510.9771233.302941

05.98764510.9771233.302941

05.98764510.9771233.302941

05.98764510.9771233.302941

0002.255087

0002.255087

0002.255087

0002.255087

0002.255087

48.82830216.43784523.27304112.578003

01.375861.8118791.303941

01.375861.8118791.303941

01.375861.8118791.303941

01.375861.8118791.303941

01.375861.8118791.303941

07.11209614.0075216.580658

07.11209614.0075216.580658

05.22046410.7281485.223928

05.22046410.7281485.223928

05.22046410.7281485.223928

01.8916323.2793731.35673

01.8916323.2793731.35673

01.8916323.2793731.35673

48.8283027.9498897.4536414.693404

48.8283027.9498897.4536414.693404

48.8283027.9498897.4536414.693404

48.8283027.9498897.4536414.693404

48.8283027.9498897.4536414.693404

0000

0000

0000

0000

0000

0000

04.662766.8926762.667903

04.662766.8926762.667903

04.662766.8926762.667903

04.662766.8926762.667903

04.662766.8926762.667903

04.662766.8926762.667903

07.35047511.85294811.008797

07.35047511.85294811.008797

07.35047511.85294811.008797

07.35047511.85294811.008797

07.35047511.85294811.008797

07.35047511.85294811.008797

016.41964125.27111914.609719

016.41964125.27111914.609719

016.41964125.27111914.609719

01.7553672.0551031.052944

01.7553672.0551031.052944

01.7553672.0551031.052944

02.3489413.4115291.49807

02.3489413.4115291.49807

02.3489413.4115291.49807

012.31533319.80448712.058705

012.31533319.80448712.058705

012.31533319.80448712.058705

03.9244016.0350892.096237

03.9244016.0350892.096237

03.9244016.0350892.096237

03.9244016.0350892.096237

03.9244016.0350892.096237

03.9244016.0350892.096237

06.22346114.4491253.023802

06.22346114.4491253.023802

06.22346114.4491253.023802

06.22346114.4491253.023802

06.22346114.4491253.023802

06.22346114.4491253.023802

0.47486811.07328218.4975169.162014

0.47486811.07328218.4975169.162014

0.47486811.07328218.4975169.162014

0.47486811.07328218.4975169.162014

0.33157711.02432318.4975169.033498

0.33157711.02432318.4975169.033498

0000.068572

0000.068572

0.1432910.04895900.059944

0.1432910.04895900.059944

17.88924731.58691459.70990227.923816

06.01977212.9753566.104638

06.01977212.9753566.104638

06.01977212.9753566.104638

06.01977212.9753566.104638

06.01977212.9753566.104638

0000

0000

0000

0000

17.88924725.56714246.73454621.819178

16.3128774.1168476.2047851.390612

0000

0000

0000

16.3128774.1168476.2047851.390612

16.3128774.1168476.2047851.390612

16.3128774.1168476.2047851.390612

1.03199516.37984733.37945317.354099

1.03199516.37984733.37945317.354099

1.03199516.37984733.37945317.354099

1.03199516.37984733.37945317.354099

04.0083486.9270413.023524
0.5443755.0704487.1503083.074467

0.5443751.06210.2232670.050943

0.5443751.06210.2232670.050943

0.5443751.06210.2232670.050943

011.11480114.5031918.227402

08.46582110.8251336.14595

08.46582110.8251336.14595

08.46582110.8251336.14595

08.46582110.8251336.14595

08.46582110.8251336.14595

02.648983.6780582.081452

02.648983.6780582.081452

02.648983.6780582.081452

02.648983.6780582.081452

02.648983.6780582.081452

03.2530278.679993.71665

03.2530278.679993.71665

03.2530278.679993.71665

03.2530278.679993.71665

03.2530278.679993.71665

03.2530278.679993.71665

0.1474282.491683.5390121.667666

0.1474282.491683.5390121.667666

0.1474282.491683.5390121.667666

0.1474282.491683.5390121.667666

0.1474282.491683.5390121.667666

0.1474282.491683.5390121.667666

835.491092167.434337151.879557110.800788

0000

0000

0000

0000

0000

835.491092167.434337151.879557110.800788

835.491092167.434337151.879557110.800788

716.318242112.12461692.91219476.60549

716.318242107.83258690.42618970.65323

716.318242107.83258690.42618970.65323

04.292032.4860055.95226

0.12940634.5755440.5150621.522558

0.12940634.5755440.5150621.522558

0.12940634.5755440.5150621.522558

96.37457910.1956677.4155337.282765

96.37457910.1956677.4155337.282765

96.37457910.1956677.4155337.282765

22.66886510.53851411.036775.389975

22.66886510.53851411.036775.389975

22.66886510.53851411.036775.389975

0000

0000

11.6476181071.48251029.083743967.578038
010.5133113.7481585.413914

10.5307581005.398368948.679229921.945853
011.22976314.1120726.715813

0.16469813.23241121.79690414.523701

0.16469813.23241121.79690414.523701

0.0528144.5065565.9605662.729262

0.0528144.5065565.9605662.729262

0.1118848.72585515.83633811.794439

0.1118848.72585515.83633811.794439

09.41828116.1521278.520239

09.41828116.1521278.520239

09.41828116.1521278.520239

09.41828116.1521278.520239

026.75822956.66363718.745481

01.137701.600029

01.137701.600029

01.137701.600029

01.7248813.2493111.261307

01.7248813.2493111.261307

01.7248813.2493111.261307

01.865423.5714931.806183

0000

0000

01.865423.5714931.806183

01.865423.5714931.806183

06.91042119.8005972.643416

06.91042119.8005972.643416

06.91042119.8005972.643416

03.1167925.8524452.436412

03.1167925.8524452.436412

03.1167925.8524452.436412

012.00301524.1897918.998134

012.00301524.1897918.998134

0000

08.97575918.7744287.146037

03.0272565.4153631.852097

0000

0000

0000

9.925099926.15141814.255838859.436884

013.08011710.4748596.220566

013.08011710.4748596.220566

013.08011710.4748596.220566

0.0095232.1972621.3811123.490255

0.0095232.1972621.3811123.490255

0.0095232.1972621.3811123.490255

03.130244.8140221.476378
6.985321661.810093487.612231680.781773

6.829402315.608367359.275583286.300316

6.829402315.608367359.275583286.300316

0.155919336.409775112.851584386.538567

06.66171110.6710426.466512

06.66171110.6710426.466512

2.930255249.063938314.787636168.94429

2.930255249.063938314.787636168.94429

2.930255249.063938314.787636168.94429

0.1887895.6324438.7146744.379344

0.1887895.6324438.7146744.379344

0.1887895.6324438.7146744.379344

0.1887895.6324438.7146744.379344

06.208228.2440865.039348

06.208228.2440865.039348

06.208228.2440865.039348

06.208228.2440865.039348

0.2521726.7676118.7398914.585043

0.2521726.7676118.7398914.585043

0.2521726.7676118.7398914.585043

0.2521726.7676118.7398914.585043

018.42125324.20442411.639801

0003.896462

0003.896462

0003.896462

0003.896462

0003.896462

0000

0000

0000

0000.626282

0000.626282

0000.626282

0000.626282

0000.195813

0000.430469

1.1168617.23210316.747756.887488

1.1168616.41249216.747754.613526

1.1168616.41249216.747754.613526

1.1168616.41249216.747754.613526

0.23863000

0.40461916.41249216.747754.613526

0.473611000

00.81961102.273962

00.81961102.273962

00.81961102.273962

00.72169302.214018

00.09791800.059944

012.56440215.4119212.776508

012.56440215.4119212.776508
0000

0000

0000

0000

012.56440215.4119212.776508

012.56440215.4119212.776508

012.56440215.4119212.776508

07.35306410.2922624.39173

07.35306410.2922624.39173

07.35306410.2922624.39173

07.35306410.2922624.39173

07.35306410.2922624.39173

37.721475504.429843303.156131296.61856
0.2146466.52858314.6686268.651283

00.9544561.0554690.574895

00.9544561.0554690.574895

00.9544561.0554690.574895

00.9544561.0554690.574895

00.9544561.0554690.574895

6.46586247.78281376.11462340.393259

00.35487600.034547

0000.034547

0000.034547

0000.034547

00.35487600

00.35487600

00.35487600

02.0682441.4978723.464429

00.01729400.034518

00.01729400.034518

00.01729400.034518

02.050951.4978723.429911

02.050951.4978723.429911

02.050951.4978723.429911

04.1518246.2824853.16295

04.1518246.2824853.16295

04.1518246.2824853.16295

04.1518246.2824853.16295

6.29314823.32319341.1544522.261791

5.94804322.50593840.41853622.050577

012.13645322.24406610.46383

012.13645322.24406610.46383

07.87966416.1189589.819454

07.87966416.1189589.819454

5.9480430.7994290.1131421.110621

5.9480430.7994290.1131421.110621

01.6903921.942370.656672

01.6903921.942370.656672

0000

0000

0000

0.3451050.8172550.7359140.211214

0.3451050.8172550.7359140.211214

0.3451050.8172550.7359140.211214

0.17271417.88467627.17981611.469542

0.1727148.41114913.1035843.65469

0.1727148.41114913.1035843.65469

0.1727148.41114913.1035843.65469

09.47352714.0762327.814852

09.47352714.0762327.814852

09.47352714.0762327.814852

14.0716598.37768210.2540055.688199

05.2635378.0805483.335115

05.2635378.0805483.335115

05.2635378.0805483.335115

05.2635378.0805483.335115

14.0716593.1141452.1734572.353084

14.0716593.1141452.1734572.353084

14.0716593.1141452.1734572.353084

14.0716593.1141452.1734572.353084

16.822862434.052313193.481391233.754344

16.822862434.052313193.481391233.754344

16.822862434.052313193.481391233.754344

16.822862434.052313193.481391233.754344

16.822862434.052313193.481391233.754344

0.1464466.7339967.5820177.55658

0.1464466.7339967.5820177.55658

0.1464466.7339967.5820177.55658

0.1464466.7339967.5820177.55658

0.1464466.7339967.5820177.55658

03.400554.6656911.89795

03.400554.6656911.89795

03.400554.6656911.89795

03.400554.6656911.89795

03.400554.6656911.89795

03.400554.6656911.89795

0000

0000

0000

0000

0000

0.49440920.57533827.7285913.807724
04.9088556.0377172.660355

03.8339495.0385092.476561

03.8339495.0385092.476561

03.8339495.0385092.476561

03.8339495.0385092.476561

03.8339495.0385092.476561

03.8339495.0385092.476561

04.1094375.0755044.482413

04.1094375.0755044.482413

04.1094375.0755044.482413

04.1094375.0755044.482413

04.1094375.0755044.482413

04.1094375.0755044.482413

02.148853.4142241.18004

02.148853.4142241.18004

02.148853.4142241.18004

02.148853.4142241.18004

02.148853.4142241.18004

02.148853.4142241.18004

0.4944095.5742478.1626363.008355

05.5742478.1626363.008355

05.5742478.1626363.008355

05.5742478.1626363.008355

05.5742478.1626363.008355

05.5742478.1626363.008355

0.494409000

0.494409000

0.494409000

0.494409000

0.494409000
